# 11^th^ National Meeting of Organic Chemistry and 4^th^ Meeting of Therapeutic Chemistry

**DOI:** 10.3390/ph9010015

**Published:** 2016-03-17

**Authors:** Maria Emília Sousa, Maria João Araújo, Maria Luísa do Vale, Paula B. Andrade, Paula Branco, Paula Gomes, Rui Moreira, Teresa M.V.D. Pinho e Melo, Victor Freitas

**Affiliations:** 1 Laboratório de Química Orgânica e Farmacêutica, Departamento de Ciências Químicas, Faculdade de Farmácia, Universidade do Porto, Rua Jorge Viterbo Ferreira 228, 4050-313 Porto, Portugal; esousa@ff.up.pt; 2 REQUIMTE/UCIBIO, Departamento de Química e Bioquímica, Faculdade de Ciências da Universidade do Porto, Rua do Campo Alegre, s/n, 4169-007 Porto, Portugal; mjaraujo@fc.up.pt; 3 REQUIMTE/UCIBIO, Departamento de Química e Bioquímica, Faculdade de Ciências da Universidade do Porto, Rua do Campo Alegre, s/n, 4169-007 Porto, Portugal; mcvale@fc.up.pt; 4 REQUIMTE/LAQV, Laboratório de Farmacognosia, Departamento de Química, Faculdade de Farmácia, Universidade do Porto, Rua de Jorge Viterbo Ferreira, 228, 4050-313 Porto, Portugal; pandrade@ff.up.pt; 5 REQUIMTE/CQFB, Departamento de Química, Faculdade de Ciências e Tecnologia, Universidade Nova de Lisboa, Monte da Caparica, 2829-516 Caparica, Portugal; p420@fct.unl.pt; 6 UCIBIO/REQUIMTE, Department of Chemistry and Biochemistry, Faculty of Sciences, University of Porto, Rua do Campo Alegre, 687, 4169-007 Porto, Portugal; pgomes@fc.up.pt; 7 Faculdade de Farmácia, Universidade de Lisboa, Av. Gama Pinto, 1649-003 Lisboa, Portugal; rmoreira@ff.ulisboa.pt; 8 Centro de Química de Coimbra, Department of Chemistry, University of Coimbra, 3004-535 Coimbra, Portugal; tmelo@ci.uc.pt; 9 REQUIMTE/LAQV, Departamento de Química e Bioquímica, Faculdade de Ciências da Universidade do Porto, Rua do Campo Alegre, s/n, 4169-007 Porto, Portugal

**Keywords:** organic synthesis, spectroscopic methods, natural compounds, drug metabolism and disposition, beyond small molecules, drug design, antitumor and anti-infective drugs, industrial applications

## Abstract

For the first time under the auspices of Sociedade Portuguesa de Química, the competences of two important fields of Chemistry are brought together into a single event, the 11st National Organic Chemistry Meeting and the the 4th National Medicinal Chemistry Meeting, to highlight complementarities and to promote new synergies. Abstracts of plenary lectures, oral communications, and posters presented during the meeting are collected in this report.

## 1. Aim and Scope of the Meeting

The Scientific Committee has put high expectations on the excellence of the scientific program, which includes plenary/keynote lectures from renowned scientists whose work has been an inspiration for researchers in Organic and Medicinal Chemistry. Oral communications focused on topics from the following main research fields: organic synthesis, spectroscopic methods, organic natural compounds, drug metabolism and disposition, beyond small molecules, computational methods and drug design, antitumor and anti-infective drugs, industrial applications.

This meeting is expected to bring together researchers with different expertise and perspectives, from senior to young scientists, to discuss and share their latest achievements in a stimulating environment, taking advantage of the inspiring atmosphere of “Teatro Municipal Campo Alegre”, a theatre that is a cultural landmark of Porto.

## 2. Lectures

### 2.1. Functionalized Nanoparticles for Alzheimer’s Disease Treatment (L3)

Barbara La Ferla, Cristina Airoldi, Francesca Re, Massimo Masserini and Francesco Nicotra *

Centre of Nanomedicine, University of Milano-Bicocca, Piazza della Scienza 2, 20126 Milano, Italy

* Correspondence: francesco.nicoyra@unimib.it

Alzheimer’s disease is associated with the formation of fibrils and plaques in the neuronal network, resulting in widespread synapsis loss and neurodegeneration. The fibrils and plaques are formed by aggregation of the strongly fibrillogenic Aβ-peptides generated by abnormal cleavage of a protein defined amyloid precursor protein. In a large integrated project, founded by the FP7 program, we generated nanoparticles properly functionalized at the surface, exploiting different chemoselective approaches. Ligands of Aβ peptides (Mourtas, S., *et al.* *Biomaterials* 2011, *32*, 1635–1645) and molecules favouring the transport through the blood brain barrier, have been selected or designed, synthesised and conjugated to the nanoparticles (Le Droumaguet, B., *et al.* *ACS Nano* 2012, *24*, 5866–5879; Sancini, G., *et al.* *J. Nanomed. Nanotechol.* 2013, *4*, 1–8).

The interaction with Aβ-peptides and the capacity to perform defibrillation of the ligands and the ligand-functionalized nanoparticles was studied with different methods including SPR and NMR.

*In vivo* studies showed the capacity of liposomes bi-functionalized with a peptide favouring the transport through the blood brain barrier and a ligand of Aβ-peptides, to ameliorate memory impairment in Alzheimer’s disease mouse models.

### 2.2. Synthesis of Porphyrin Derivatives with Antitumoral and Antimicrobial Activity (L5)

Mariana Q. Mesquita, José C.J.M.D.S. Menezes, Joana F.B. Barata, Eliana Alves and Maria A.F. Faustino *

QOPNA and Department of Chemistry, University of Aveiro, 3810-193 Aveiro, Portugal

* Correspondence: faustino@ua.pt

Porphyrins and analogues due to their unique physico-chemical features are finding applications in different fields like artificial photosynthesis, catalysis, sensors, nanomaterials and medicine. In medicine, these compounds are being used with high success as photosensitizers (PS) in Photodynamic Therapy to treat oncological and non-oncological situations like infections caused by microorganisms (Alves, E., *et al.* *J. Photochem. Photobiol. C Photochem. Rev.* 2015, *22*, 34–57.). In this therapy, the photoactivation of the PS by visible light in the presence of molecular oxygen affords highly cytotoxic reactive oxygen species (ROS) that are responsible by the death of target cells (e.g., tumoral or microbial). Although the ability of a PS to generate ROS, namely singlet oxygen (^1^O_2_) is important for an efficient PDT effect, the structural feature of PS is another crucial aspect that is dependent on the target. Herein will be discussed some recent synthetic strategies developed in the group to obtain PS with adequate solubility in physiological media, to improve their selectivity to target tumoral cells or to photoinactivate microorganisms, to have better penetration on the tissue and also to allow their immobilization in solid supports (Mesquita, M.Q., *et al. Dyes Pigment.* 2014, *110*, 123–133; Alves, E., *et al. Bioorg. Med. Chem*. 2013, *21,* 4311–4318; Barata, J.F.B., *et al.* *Eur. J. Med. Chem.* 2015, *92*, 135–144).

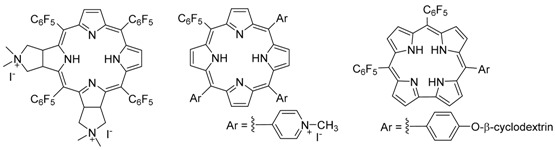


**Acknowledgments:** Thanks are due to the University of Aveiro, Fundação para a Ciência e a Tecnologia (FCT, Portugal), European Union, QREN, COMPETE and FEDER for funding the QOPNA research unit (project PEst-C/QUI/UI0062/2013, FCOMP-01-0124-FEDER-037296).

### 2.3. Chemical Biology Approaches to Target Validation in the Ubiquitin and Chromatin Systems (L7)

Alessio Ciulli

School of Life Sciences, Division of Biological Chemistry and Drug Discovery, University of Dundee, Dow Street, DD1 5EH, DD15EH Dundee, UK; E-Mail: a.ciulli@dundee.ac.uk

This lecture will outline my laboratory’s recent progress and current efforts at developing chemical tools to study and target complex molecular players in the ubiquitin-proteasome system (UPS) and epigenetics. These fundamental biological pathways are rich in potential drug targets identified by genetic or cell biology studies as important to human physiological and pathophysiology states, including cancer. However, most of these targets are still perceived as “undruggable” to conventional medicinal chemistry and remain to be fully validated chemically e.g., by means of high-quality and selective chemical probes, to truly enable the burgeoning opportunities for new therapeutics.

One such class of attractive yet challenging targets are cullin RING E3 ubiquitin ligase (CRL), multisubunit enzymes that impart substrate specificity to ubiquitination in the UPS (Bulatov, E., *et al.* *Biochem. J.* 2015, *467*, 365–386). CRL-targeting chemical tools can be used alone as E3 ligase inhibitors that modulate the pathway in which the specific CRL is involved. First, I will describe our discovery of potent inhibitors of the protein-protein interaction between the von Hippel-Lindau (VHL) CRL and its substrate protein HIF-1α using structure-guided design (Galdeano, C., *et al. J. Med. Chem.* 2014, *57*, 8657–8863; Van Molle, I., *et al. Chem. Biol.* 2012, *19*, 1300–1312). Mechanistic and cellular characterization of our VHL inhibitors provide the foundation for them to be widely used as new selective probes in the hypoxic signaling pathway. In addition, CRL-targeting ligands can be suitably conjugated with any protein ligands, yielding bifunctional proteolysis targeting chimeras (PROTACs) to hijack the UPS and induce the intracellular degradation of the target protein. I will illustrate how selective destruction of the epigenetic transcriptional regulator Brd4 was achieved by tethering the pan-selective BET bromodomain inhibitor JQ1 to a VHL ligand.3 Our PROTAC molecule MZ1 induces rapid, time-dependent, long-lasting, and dose-dependent preferential removal of Brd4 over its homologous BET-family members Brd2 and Brd3 in cancer cells, leading to a more limited transcriptional response of MZ1 compared to JQ1, consistent with that of Brd4 RNAi (Zengerle, M., *et al.* *ACS Chem. Biol.* 2015, *10*, 1770–1777). This study provides proof-of-concept for inducing the selective degradation of any protein of interest using PROTACs.

In a separate study, we reported a general strategy to introduce controlled selectivity of BET bromodomain inhibitors via a bump-and-hole approach (Baud, M.G., *et al.* *Science* 2014, *346*, 638-641). We developed compound ET, an ethyl derivative of JQ1, so that it can bind potently and with high selectivity (up to 540-fold) to a specifically-designed mutant bromodomain. We applied this method inside cancer cells to show that blockade of the first bromodomain alone is sufficient to displace Brd4 from chromatin (Baud, M.G., *et al. Science* 2014, *346*, 638–641). Finally, I will outline how we are developing and optimizing this technology and applying it to address specific questions for BET-protein target validation.

### 2.4. Multi-Target-Directed-Ligands for Therapy of Alzheimer’s Disease (L8)

José Marco-Contelles

Laboratory of Medicinal Chemistry (Institute of Organic Chemistry, CSIC); 3, Juan de la Cierva; 28006-Madrid, Spain; E-Mail: iqoc21@iqog.csic.es

[*N*-((5-(3-(1-benzylpiperidin-4-yl)propoxy)-1-methyl-1*H*-indol-2-yl)methyl)-*N*-methylprop-2-yn-1-amine], **ASS234** (Bolea, I., *et al.* *J. Med. Chem*. 2011, *54*, 8251–8270), is a new multipotent molecule derived from **donepezil** (Aricept^®^), a currently acetylcholinesterase inhibitor prescreibed to Alzheimer’s disease (AD) patients, and **PF9601N**, a potent and selective MAO B inhibitor (Pérez, V., *et al.* *Brit. J. Pharmacol.* 1999, *127*, 869–876), for the potential treatment of AD.

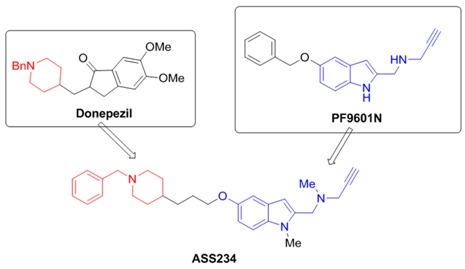


We have found that **ASS234** is able to inhibit ChE and MAO enzymes, showing also neuroprotection against diverse toxic insults, and antioxidant properties (Bautista-Aguilera, O.M., *et al.* *J. Med. Chem*. 2014, *57*, 10455–10463; Esteban, G., *et al. BBA Proteins and Proteomics* 2014, *1844*, 1104–1110; del Pino, J., *et al.* *CNS Neurosci. Ther.* 2014, *20*, 568–570; Stasiak, A., *et al.* *Curr. Pharm. Des.* 2014, *20*, 161–171; Bolea, I., *et al.* *Curr. Alzheimer Res.* 2013, *9*, 797–808).

### 2.5. Targeted Nanomedicines for Cancer Therapy (L9)

Hélder A. Santos

Division of Pharmaceutical Chemistry and Technology, Faculty of Pharmacy, University of Helsinki, FI-00014 Helsinki, Finland; E-Mail: helder.santos@helsinki.fi

Novel biomedical engineering technologies have been underlined as very promising means for the advance in medical research (Santos, H.A., *et al.* *J. Nanomedicine* (London) 2012, *7*, 1281–1284; Shrestha, N., *et al.* *Biomaterials* 2014, *35*, 7172–7179; Shrestha, N., *et al.* *Biomaterials* 2015, *68*, 9–20). Personalized medicine allows for the identification of the right therapy, reaching the right therapeutic target in the body at the right time in an efficient manner, with reduced undesired collateral effects (Santos, H.A., *et al.* *Nanomedicine* (London) 2014, *9*, 535–554). In this context, target nanomedicines are of great interest towards the development of personalized medicines and envisaged for their large-scale implementation.

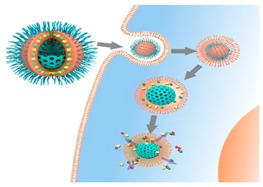


Recently, we have developed prominent biomaterials, such as porous silicon and polymer-based micro- and nanoparticles as potential platforms for the individualization of medical intervention (Araújo, F., *et al.* *ACS Nano* 2015, *9*, 8291; Kong, F., *et al.* *Adv. Funct. Mater.* 2015, *25*, 3330–3340; Zhang, H., *et al.* *Adv. Mater.* 2014, *26*, 4497–4503, Liu, D., *et al.* *Adv. Mater.* 2015, *27*, 2298–2304; Herranz-Blanco B., *et al.* *Adv. Funct.* *Mater.* 2015, *25*, 1448–1497; Liu, D., *et al.* *Biomaterials* 2015, *39*, 249–259), including intracellular targeting of chemical-modified nanomaterials for cancer therapy. All these biomaterials are promising advanced drug delivery technologies for biomedical applications. The results of the efficient surface biofunctionalization, targeting, encapsulation of drug molecules and other bioactive compounds, of these biomaterials using advanced technologies, such the microfluidics technique are presented and discussed in detail. Examples on how these materials can be used to enhance the bioavailability of drug/peptide molecules, demonstrating their cytocompatibility, and *in vivo* biocompatibility and intracellular cancer targeting, are also presented. Overall, the recent cutting-edge advances on nanomaterials are anticipated to overcome some of the therapeutic window and clinical applicability of many drug/peptide molecules, and can also act as innovative theranostic platforms and tools for the clinic, because they offer a less invasive alternative compared to the conventional therapeutic strategies and, thereby, enhancing the expectancy and quality of life of the patients.

**Acknowledgments:** The Academy of Finland (decisions No. 252215 and 281300), the University of Helsinki Research Funds, the Biocentrum Helsinki, and the European Research Council under the European Union’s Seventh Framework Programme (FP/2007–2013, grant No. 310892) are highly acknowledge for financial support.

### 2.6. Looking for a Needle in a Haystack with the Right Tools: The Discovery of Potent Transthyretin Amyloid Inhibitors (L11)

Rui M.M. Brito ^1,2,3,^*, Carlos J.V. Simões ^2^, Bruno L. Victor ^1,2^, Zaida L. Almeida ^1,3^, Dora S. Costa ^1^, Bruno O. Nascimento ^1,3^, Ana L. Cardoso ^1^, Teresa M.V.D. Pinho e Melo ^1^, Maria R. Almeida ^4^ and Maria J.M. Saraiva ^4^

^1^ Coimbra Chemistry Centre, Department of Chemistry, University of Coimbra, 3004-535 Coimbra, Portugal

^2^ BSIM^2^—Biomolecular Simulations Lda, Biocant Park, 3060-197 Cantanhede, Portugal

^3^ Center for Neuroscience and Cell Biology, University of Coimbra, 3004-504 Coimbra, Portugal

^4^ Institute for Molecular and Cell Biology, University of Porto, 4150-180 Porto, Portugal

* Correspondence: brito@ci.uc.pt

The aggregation of proteins into insoluble amyloid fibrils is the hallmark of many, highly debilitating, human pathologies such as Alzheimer’s and Parkinson’s diseases, or rare neurodegenerative diseases like Familial Amyloid Polyneuropathy (FAP). FAP is an amyloid disease caused by mutations in the protein transthyretin (TTR) and characterized by progressive peripheral and autonomic polyneuropathy. It manifests by early impairment of pain and heat sensations in the lower limbs and progresses to a general lowering of the state of health and severe autonomic dysfunction with fatal consequences in many patients, if untreated, 10–15 years after the onset of the first symptoms.

TTR is a homotetrameric protein mainly synthesized in the liver, the choroid plexus, and the retina, forming independent TTR pools in the plasma, cerebrospinal fluid and the eye, respectively. Liver transplantation (LT) has been the standard treatment option for FAP for nearly two decades. LT halts progression of clinical symptoms by replacing the main organ producing the disease-associated mutant TTR. More recently, tafamidis meglumine (brand name Vyndaqel) reached the European and Japanese drug markets as the first drug therapy directed to the treatment of FAP. Tafamidis has shown that stabilization of the native tetrameric form of TTR by molecules endowed with chaperone-like activity is a viable approach to prevent (or at least stall) the formation of amyloid aggregates and fibrils, thus delaying disease progression. However, tafamidis demonstrated improvement of symptoms in only approximately 60% of the FAP patients.

Here, we report on successful efforts to discover new chemical entities (NCEs) with better activity profiles for TTR amyloid inhibition *in vitro* and TTR stabilization in human plasma. Lead discovery and optimization was carried out *in silico* followed by experimental *in vitro*, *ex vivo* and *in vivo* validation. Three novel lead compound series (AT09, AT40 and AT50) have been identified, characterized and expanded.

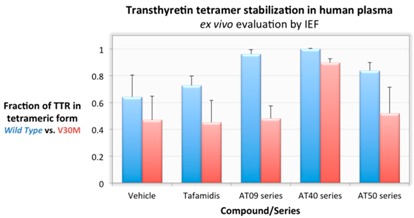


The best compounds in each series display *in vitro* inhibitory activity unmatched by any known inhibitor of transthyretin-related amyloid. Some of the compounds are almost insensitive to TTR mutations and display very high TTR stabilization activity *ex vivo* (in plasma of V30M-TTR carriers). Additionally, several compounds in each series display very favorable solubility and toxicity profiles.

**Acknowledgments:** This work was funded in part by the European Regional Development Fund (ERDF) through the COMPETE Programme and Mais Centro—Programa Operacional do Centro (QREN/SI-IDT/21622) and by National Funds through FCT—Fundação para a Ciência e a Tecnologia (PTDC/QUIQUI/122900/2010).

### 2.7. Chemical Mechanisms of Drug Toxicity—Lessons from Nevirapine (L12)

Maria Matilde Marques

Centro de Química Estrutural, Instituto Superior Técnico, Universidade de Lisboa, P1049-001 Lisboa, Portugal; E-Mail: matilde.marques@tecnico.ulisboa.pt

Nevirapine (**NVP**, **1**) is a non-nucleoside reverse transcriptase inhibitor (NNRTI) widely used in combined antiretroviral therapy and to prevent mother-to-child transmission of the human immunodeficiency virus type 1. NVP’s clinical efficacy, along with low cost, favourable lipid profile, and suitability for use during pregnancy largely account for the widespread use of the drug (Lockman, S., *et al.* *N. Engl. J. Med*. 2007, *356,* 135). Despite its benefits, NVP is associated with immune-mediated hepatotoxicity and skin rash, which can be fatal and are major causes of drug discontinuation. In addition, although direct correlation between NVP-based therapies and human cancer has yet to be demonstrated, long-term administration of the drug to rodent models resulted in increased incidences of hepatocellular adenomas and carcinomas (PDR staff, VIRAMUNE^®^ (nevirapine), *In* *Physicians’ Desk Reference*, 63rd ed., Physicians’ Desk Reference, Inc., Montvale, NJ, USA, 2009; 873); moreover, epidemiological data indicated an association between the chronic use of NNRTIs and the occurrence of non-AIDS-defining cancers in HIV-positive patients (Powles, T., *et al.* *J. Clin. Oncol.* 2009, *27*, 884).

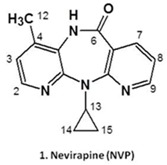


While the exact mechanisms underlying NVP-induced toxic events are still not fully understood, a considerable amount of work on the development of mass spectrometry-based tools to assess covalent NVP-protein and NVP-DNA adducts as biomarkers of metabolic activation has been conducted in recent years. The rationale for using these biomarkers is based on evidence that NVP is bioactivated to reactive metabolites able to modify biomacromolecules by forming covalent adducts that may trigger carcinogenic and immune-mediated processes (Marinho, A.T., *et al.* *J. Antimicrob. Chemother*. 2014, *69*, 476).

The current presentation addresses our efforts to assess the contribution of benzylic (at C12) *versus* aromatic (at C2/C3) hydroxylation of NVP to the generation of electrophilic metabolites prone to bind biomacromolecules *in vitro* and *in vivo*. The ensuing implications to NVP’s toxicity are discussed.

**Acknowledgments:** Thanks are due to the Portuguese NMR and MS Networks (IST-UL nodes) for providing access to the facilities. This work was supported in part by Fundação para a Ciência e a Tecnologia (FCT), Portugal, through grants UID/QUI/00100/2013, RECI/QEQ-QIN/0189/2012 and RECI/QEQ-MED/0330/2012.

## 3. Oral Presentations

### 3.1. Antitumor Activity of TXA1, an Autophagy Inducer which Affects Cholesterol Localization (MC1)

Raquel T. Lima ^1,2,3^, Diana Sousa ^1,2,4^, Ana Sara Gomes ^5,6^, Nuno Mendes ^1^, Rune Matthiesen ^7^, Madalena Pedro ^8^, Madalena M. Pinto ^6,9^, Emília Sousa ^6,9^ and M. Helena Vasconcelos ^1,2,4,^*

^1^ i3S-Instituto de Investigação e Inovação em Saúde, Universidade do Porto, Rua Alfredo Allen 208, 4200-135 Porto, Portugal

^2^ Cancer Drug Resistance Group, IPATIMUP—Institute of Molecular Pathology and Immunology of the University of Porto, Rua Júlio Amaral de Carvalho 45, 4200-135 Porto, Portugal

^3^ Department of Pathology and Oncology, Faculty of Medicine of the University of Porto, Alameda Hernâni Monteiro, 4200-319 Porto, Portugal

^4^ Laboratory of Microbiology, Department of Biological Sciences, Faculty of Pharmacy of the University of Porto, Rua de Jorge Viterbo Ferreira 228, 4050-313 Porto, Portugal

^5^ UCIBIO/REQUIMTE, Universidade do Porto, Rua Jorge Viterbo Ferreira 228, 4050-313 Porto, Portugal

^6^ Laboratório de Química Orgânica e Farmacêutica, Departamento de Ciências Químicas, Faculdade de Farmácia, Universidade do Porto, Rua Jorge Viterbo Ferreira 228, 4050-313 Porto, Portugal

^7^ National Health Institute, Ricardo Jorge, IP, Av. Padre Cruz, 1649-016 Lisbon, Portugal

^8^ Cooperativa de Ensino Superior, Politécnico e Universitário (CESPU), Centro de Investigação em Ciências da Saúde (CICS), Instituto Superior de Ciências da Saúde—Norte (ISCS-N), Rua Central de Gandra, 1317, 4585-116 Gandra, Portugal

^9^ Centro Interdisciplinar de Investigação Marinha e Ambiental (CIIMAR/CIMAR), Universidade do Porto, Rua dos Bragas 289, 4050-123 Porto, Portugal

* Correspondence: hvasconcelos@ipatimup.pt

Some of us have previously identified TXA1 as a hit thioxanthone with antitumor potential (Palmeira, A., *et al.* *Biochem. Pharmacol.* 2012, *83*, 57–68). The present study aimed to investigate its mechanism of action *in vitro* and in human non-small cell lung cancer (NSCLC) cells xenografted in nude mice.

TXA1 presented antitumor activity associated with the induction of autophagy and apoptosis, in melanoma and NSCLC cell lines. Interestingly, this molecule (soluble salt) affected lipid biosynthesis and resulted in an abnormal cellular cholesterol localization in NSCLC cells. The soluble salt of TXA1 was not toxic to nude mice, significantly reducing the growth of human NSCLC cells xenografts. Overall this study provides new insights into the mechanism of action of a novel small molecule, which may be relevant for the development of anticancer strategies.

**Acknowledgments:** Fundação para a Ciência e Tecnologia-FCT for post-doc grant: SFRH/BPD/68787/2010. This research was partially supported by the Strategic Funding UID/Multi/04423/2013 through national funds provided by FCT—Foundation for Science and Technology and European Regional Development Fund (ERDF), in the framework of the programme PT2020.

### 3.2. Spirooxadiazoline Oxindoles with Promising in Vitro Antitumor Activities (MC3)

Carlos J. A. Ribeiro, Joana D. Amaral, Cecilia M. P. Rodrigues, Rui. Moreira and Maria M. M. Santos *

Research Institute for Medicines (iMed.ULisboa), Faculty of Pharmacy, Universidade de Lisboa, Av. Gama Pinto, 1649-003 Lisboa, Portugal

* Correspondence: mariasantos@ff.ulisboa.pt

Tumor suppressor p53 is a transcription factor widely regarded as the “guardian of the genome” that plays an important role in the regulation of several biological processes. So, it is not surprising that the p53-signaling pathway is inactivated in all types of cancers and that restoring p53 function in cancer cells represents a valuable anticancer approach (Hoe, K.K., *et al.* *Nat. Rev. Drug Discov.* 2014, *13*, 217). In tumors that retain wild-type p53 but have defects in p53 regulatory pathways, the main goal is to inhibit the function of its negative regulators, such as MDM2. Generally, p53-MDM2 interaction inhibitors contain three lipophilic groups attached to a rigid heterocyclic scaffold to mimic the three most important p53 amino acids (Phe19, Trp23 and Leu26) that interact with MDM2. Furthermore, all the interactions are primarily hydrophobic, with potency increasing essentially by introduction of halide-substituted aromatic groups. Based on this information, we developed several novel chemical scaffolds with potential anticancer activity (Ribeiro, C.J.A., *et al. Bioorg. Med. Chem.* 2014, *22*, 577–584; Soares, J., *et al. Eur. J. Pharm.* *Sci.* 2015, *66*, 138–147; Soares, J., *et al. Pharmacol.* *Res.* 2015, *95–96*, 42–52; Monteiro, A., *et al.* *Eur. J. Med. Chem.* 2014, *79*, 266–272). Herein, we report the synthesis of a library of spirooxadiazoline oxindoles and the biological evaluation as p53-MDM2 interaction inhibitors. The most active compound showed a GI_50_ value of 1.7 µM in HCT 116 p53^(+/+)^ cell line, representing a 15.4-fold increase in potency when compared to the most active spiroisoxazoline oxindole obtained previously (Ribeiro, C.J.A., *et al. Bioorg. Med. Chem.* 2014, *22*, 577–584). Together, our results indicate that spirooxadiazoline oxindoles reduce the p53 inhibition by MDM2, subsequently increasing the expression levels of p53 target genes, representing a promising scaffold for the development of novel anticancer agents (Ribeiro, C.J.A., *et al.* *MedChemComm.* 2016, doi:10.1039/C5MD00450K).

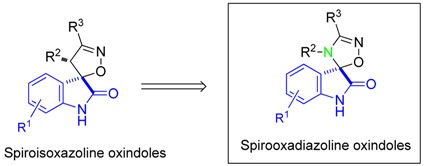


### 3.3. Surpassing Multidrug Resistance in Cancer: A Study on Jolkinol D Derivatives (MC4)

Mariana A. Reis ^1^, Omar B. Ahmed ^2^, Gabriella Spengler ^3^, Hermann Lage ^2^ and Maria-José U. Ferreira ^1,^*

^1^ Research Institute for Medicines (iMed.ULisboa), Faculty of Pharmacy, Universidade de Lisboa, Avenue Gama Pinto, 1649-003 Lisbon, Portugal

^2^ Institute of Pathology, University Hospital Charité, 10117 Berlin, Germany

^3^ Department of Medical Microbiology and Immunobiology, Faculty of Medicine, University of Szeged, Dómtér 10, H-6720 Szeged, Hungary

* Correspondence: mjuferreira@ff.ulisboa.pt

Cancer multidrug resistance (MDR) accounts for 90% of chemotherapy failure. The development of P-glycoprotein (Pgp) modulators and collateral sensitivity (CS) agents are promising strategies to surpass MDR (Kathawala, R., *et al.* *Drug Resist. Updat.* 2015, *18*, 1–17). Macrocyclic lathyranes have an anti-MDR potential, however optimization of these molecules is in progress (Ferreira, M.J.U., *et al.* *Phytochem. Rev.* 2014, *13*, 915–935). Hence, our goal is to optimize macrocyclic lathyranes as new leads for MDR reversal.

Twenty seven lathyranes were achieved by molecular derivatization of jolkinol D with acylating reagents. The MDR reversal activity was evaluated using mouse T-lymphoma MDR1-transfected cells. Promising Pgp modulators should be lipophilic and have an aroyl moiety as highlighted in structure-activity relationships studies. Drug combination experiments also corroborated their anti-MDR potential.

CS effect was evaluated against gastric (EPG85-257) and pancreatic (EPP85-181) human cancer cells and their drug-selected counterparts (EPG85-257RDB and EPP85-181RDB), using a proliferation assay. Two derivatives decreased the resistance of EPG85-257RDB cells in 65%. In pancreatic cells, levels of resistance reduced to 32%–65%. This selective cytotoxicity occurred through caspase-dependent apoptosis.

**Acknowledgments:** Fundação para a Ciência e a Tecnologia (FCT), Portugal (project PTDC/QEQ-MED/0905/2012 and Ph.D. grant SFRH/BD/72915/2010). German Egyptian Research Long-term Scholarship (GERLS) Programme 2014 (57076387) provided by the German Academic Exchange Service (DAAD).

### 3.4. Pharmaceutical Ionic Liquids and Salts as Antitumor Agents (MC9)

Miguel M. Santos ^1,^*, Ricardo Ferraz ^2^, Sónia Teixeira ^1,3^, João Costa-Rodrigues ^3^, João P. Noronha ^1^, Zeljko Petrovski ^1^ and Luis C. Branco ^1^

^1^ LAQV-REQUIMTE, Faculdade de Ciências e Tecnologia da Universidade Nova de Lisboa, 2829-516 Caparica, Portugal

^2^ Ciências Químicas e das Biomoléculas, Escola Superior de Tecnologia da Saúde do Porto do Instituto Politécnico do Porto, Rua Valente Perfeito 322, 4400-330 Vila Nova de Gaia, Portugal

^3^ Laboratório de Farmacologia e Biocompatibilidade Celular, Faculdade de Medicina Dentária, Universidade do Porto, Rua Manuel Pereira da Silva, 4200-393 Porto, Portugal

* Correspondence: miguelmms@gmail.com

Synthesis of Ionic Liquids from Active Principle Ingredients (API-ILs) has been the main focus of our group for the last years. The combination of APIs as anions or cations with appropriate organic counter ions can be an innovative solution to the polymorphism behavior of several drugs as well as to improve their water solubility, permeability and corresponding bioavailability and biological activity (Ferraz, R., *et al.* *ChemMedChem* 2011, *6*, 975–985; Marrucho, I.M., *et al. Annu. Rev. Chem. Biomol. Eng.* 2014, *5*, 527–546). Within this context, novel ionic liquids with anti-cancer properties and decreased toxicity have recently been investigated.

In this communication we present the anti-proliferative effect against diverse tumor cell lines of novel Ionic Liquids based on anionic Ampicillin (**1**) and Bisphosphonates (**2**) combined with appropriate biocompatible organic cations, e.g., choline, cetylpyridinium and alkylimidazolium.

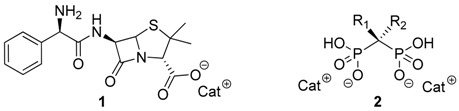


This approach has conferred antitumor activity against five different human cancer cell lines with IC50 values in the low micromolar/nanomolar ranges to Ampicillin while showing enhanced antibacterial properties against sensitive and Gram-negative resistant bacteria (Ferraz, R., *et al. ChemMedChem* 2015, *10*, 1480–1483; Ferraz, R., *et al. RSC Adv.* 2014, *4*, 4301–4307; Florindo, C., *et al. Int. J. Pharm.* 2013, *456*, 553–559; Ferraz, R., *et al. Med. Chem. Commun.* 2012, *3*, 494–497). On the other hand, by taking advantage of the high affinity of bisphosphonates towards bone tissue (Ben-Aharon, I., *et al. PLoS ONE* 2013, *8*, e70044), the new API-ILs based on these compounds have also been tested against related cancer cell lines. The discussion will be complemented with the study of different physicochemical properties.

### 3.5. Hybrid Compounds for the Treatment of Glioma: A New Approach (MC10)

Cláudia Braga ^1,^*, Maria Perry ^1^, Rui Moreira ^1^, Dora Brites ^2^, Rui Pinheiro ^2^, Mario Varasi ^3^ and Ana Falcão ^2^

^1^ Medicinal Chemistry Group, Research Institute for Medicines (iMed.ULisboa), Faculty of Pharmacy, Universidade de Lisboa, Av. Gama Pinto, 1649-003 Lisbon, Portugal

^2^ Neuron-Glia Biology in Health and Disease Group, Research Institute for Medicines (iMed.ULisboa), Faculty of Pharmacy, Universidade de Lisboa, Av. Gama Pinto, 1649-003 Lisbon, Portugal

^3^ Drug Discovery Unit, Department of Experimental Oncology, European Institute of Oncology, Via Adamello 16-20139 Milan, Italy

* Correspondence: claudiabraga@campus.ul.pt

Glioma is a type of primary brain tumour that arises from glial cells. The most common of malignant gliomas, glioblastoma multiforme, has a median survival of approximately 14 months after diagnosis (Sathornsumetee, S., *et al.* *Ann*. *N.Y. Acad. Sci*. 2000, *1142*, 108–132).

Temozolomide is a triazene alkylating agent used for the treatment of gliomas. However, its therapeutic effectiveness is often disappointing, largely in consequence of the lack of selectivity for tumor cells, insufficient drug concentration in the tumor and notorious resistance (Agarwala, S.S., *et al.* *Oncologist* 2000*,* *5*, 144–151).

Valproic acid is an anticonvulsant used in the treatment of epilepsy. Recently, it was shown to inhibit a subset of histone deacetylases (HDAC), that consequently leads to the inhibition of DNA repair, thereby potentiating cytotoxic treatments such as chemotherapy or radiation therapy (Kazantsev, A.G., *et al.* *Nat. Rev. Drug Discov*. 2008, *7*, 854–68).

Our team has been involved in the design of novel hybrid compounds with two units, a triazene as an alkylating agent and a carboxylic acid with known HDAC inhibitory activity. Preliminary results towards glioma cell line (GL261), demonstrated that the first hybrid compound tested has improved efficacy compared to Temozolomide. In this research work we report the evaluation of hybrid compounds. The stability of the compounds in phosphate buffer pH 7.4 and human plasma (80% *v*/*v*) were evaluated by HPLC. Our first results demonstrated a high stability in physiological conditions. The lipophilicity of the hybrid compounds is described. The screening for their inhibitory activity against HDAC showed improved activity comparing to the parental HDAC inhibitor, however, the exact mechanism of action is yet to be fully determined.

### 3.6. Development of Brain Permeant Peptidomimetic β-Secretase Inhibitors for Alzheimer’s Disease (MC12)

Helder Vila-Real ^1,2,^*, Helena Coelho ^1,2^, João Rocha ^3^, Adelaide Fernandes ^3^, Maria Rita Ventura ^2^, Christopher D. Maycock ^2,4^, Olga Iranzo ^2^ and Ana L. Simplício ^1,2^

^1^ Instituto de Biologia Experimental e Tecnológica, Av. da República, Quinta do Marquês, 2780-157 Oeiras, Portugal

^2^ Instituto de Tecnologia Química e Biológica, Av. da República, Quinta do Marquês, 2780-157 Oeiras, Portugal

^3^ Faculdade de Farmácia, Universidade de Lisboa, Av. Prof. Gama Pinto, 1649-003 Lisboa, Portugal

^4^ Faculdade de Ciências, Universidade de Lisboa, Campo Grande, 1749-016 Lisboa, Portugal

* Correspondence: hvreal@itqb.unl.pt

β-secretase (BACE-1) inhibitors are potential useful drugs for the management of Alzheimer’s disease (AD), but their incapacity to cross the blood-brain barrier and reach the Central Nervous System (CNS) is still a major reason for failure (Butini, S., *et al.* *Curr. Top. Med. Chem*. 2013, *13*, 1787–1807). In this work we have tested the hypothesis of whether the conjugation of a peptidomimetic inhibitor, OM00-3, with a β-amyloid peptide sequence, Aβ18-23, facilitates its delivery into the brain.

Inhibitors were synthesized by Solid Phase Peptide Synthesis. Their potency against BACE-1/2, cytotoxicity in Caco-2 cells, metabolization in serum and mice brain were determined. A pharmacokinetic assay was performed in mice.

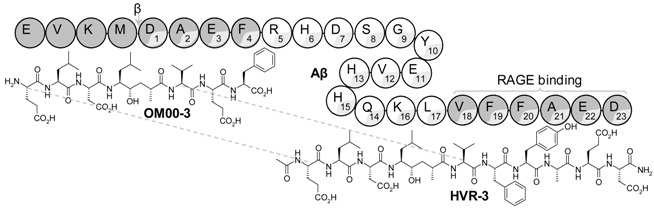


HVR-3 was found to be as potent as OM00-3 but 4-fold more selective toward BACE-1 in relation to BACE-2 and also 3-fold more stable against *in vitro* metabolization in Human serum. Intravenous administration to mice generated an active metabolite recovered from the rodent’s brain. The success of this conjugation strategy to target the CNS corroborates the potential of HVR-3 as new anti-AD drug (Vila-Real, H., *et al. J. Med. Chem*. 2015, *58*, 5408–5418).

**Acknowledgments:** We acknowledge Fundação para a Ciência e a Tecnologia for financial support: SFRH/BPD/82097/2011.

### 3.7. Fatty Acids from Edible Sea Hares: Anti-Inflammatory Capacity in LPS-Stimulated RAW 264.7 Cells Involves iNOS Modulation (MC14)

Renato B. Pereira, Andreia P. Oliveira, Patrícia Valentão and Paula B. Andrade *

REQUIMTE/LAQV, Laboratório de Farmacognosia, Departamento de Química, Faculdade de Farmácia, Universidade do Porto, R. Jorge Viterbo Ferreira, 228, 4050-313 Porto, Portugal

* Correspondence: pandrade@ff.up.pt

In recent years, marine macroinvertebrates gained great importance by their fatty acid composition. Sea hares of *Aplysia* genus are known to be consumed, in oriental countries (Titcomb, M., *et al.* *Pac. Sci.* 1978, *32*, 325–386); however, their nutritional composition and potential health effects are nearly unknown. In the present study we intended to characterize the fatty acids composition and evaluate the anti-inflammatory potential of lipophilic extracts of two sea hares, *Aplysia fasciata* Poiret and *Aplysia punctata* Cuvier.

Twenty-five fatty acids were identified, nine of them not yet reported in these species. Both extracts revealed similar anti-inflammatory properties in the culture medium of LPS-stimulated macrophages, as proved by the decreased ^•^NO levels. A similar decrease was also observed in L-citrulline levels, indicating a possible modulation of inducible nitric oxide synthase (iNOS) by the action of the compounds found in the extracts (A). Regarding lipoxygenase (LOX) inhibition, *A. punctata* extract was more effective (B), probably because it contains more polyunsaturated fatty acids (PUFA) that can compete with linoleic acid for the active site.

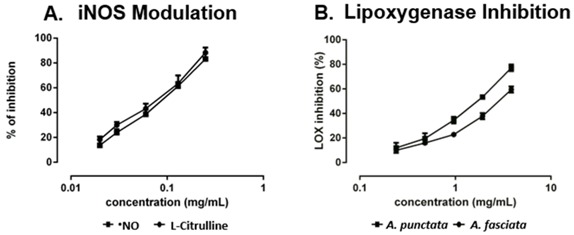

Effect in ^•^NO and l-citrulline levels of RAW 264.7 cells pre-treated with *Aplysia fasciata* extract (**A**) and inhibition of soybean lipoxygenase by extracts of *Aplysia* spp. (**B**).

Overall, the results indicate that, in addition to their direct ingestion, *A. fasciata* and *A. punctata* may be good sources of nutraceuticals providing beneficial health effects, by reducing the levels of inflammatory mediators involved in the genesis of several diseases.

**Acknowledgments:** This work received financial support from the European Union (FEDER funds through COMPETE) and National Funds (FCT, Fundação para a Ciência e Tecnologia) through project UID/QUI/50006/2013. R. Pereira is grateful to i3DU program for Ph.D. fellowship (PD/BD/113565/2015).

### 3.8. Design, Synthesis and Biological Evaluation of Novel Anti-Bacterial Agents (MC15)

Filipa Ramilo-Gomes ^1,^*, Pedro Adão ^1^, Sílvia A. Sousa ^2^, Jorge H. Leitão ^2^, João Costa Pessoa ^1^ and Maria Matilde Marques ^1^

^1^ Centro de Química Estrutural, Instituto Superior Técnico, Universidade de Lisboa, 1049-001 Lisboa, Portugal

^2^ Institute for Bioengineering and Biosciences, Instituto Superior Técnico, Universidade de Lisboa, 1049-001 Lisboa, Portugal

* Correspondence: filipa.ramilo.gomes@tecnico.ulisboa.pt

Aromatic Schiff bases represent versatile pharmacophores with potential antimicrobial properties. As part of a program aimed at identifying new drug candidates with multi-target antibacterial activity, we have been exploring several classes of bis-hydrazone compounds and their iron complexes.

The antimicrobial activities of the synthesized compounds against the Gram-positive *S. aureus* Newman and the Gram-negative *P. aeruginosa* 477 were assessed using the Disk Diffusion Test. The Minimum Inhibition Concentrations (MICs) were also determined. Although the results are still preliminary, moderate activities were observed for compounds **1** and **7**. The results obtained will be discussed on the basis of structure-activity relationships.

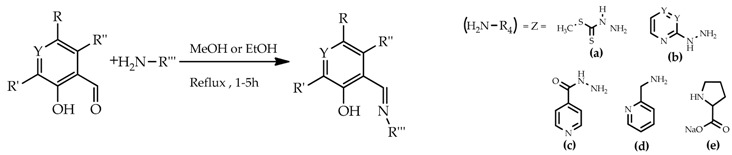

**Compound****1****2****3****4****5****6****7****8****9****10****11****R_1_**CH_3_C(CH_3_)_3_CH_3_
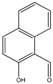
CH_3_HHHCH_3_HCH_3_**R_2_**(a)CHOCHOCHOHOCH_3_CH_3_CHOHCHO**R_3_**HHHHHHCH2OHHHH**Y**CCCCCCNCCC**Z**(b)(e) (b)(d)(a)(a)(a)(a)(c)(c)(c)**complex**ironiron---------

**Acknowledgments:** We thank FCT for financial support (UID/QUI/00100/2013 and Incentivo-UI100-2014).

### 3.9. Herbal Medicines: A Source of Phenolic Monoamine Oxidase A Inhibitors (MC16)

Clara Grosso, Patrícia Valentão and Paula B. Andrade *

REQUIMTE/LAQV, Laboratório de Farmacognosia, Departamento de Química, Faculdade de Farmácia, Universidade do Porto, Rua de Jorge Viterbo Ferreira, 228, 4050-313 Porto, Portugal

* Correspondence: pandrade@ff.up.pt

The use of herbal drugs for improving health and for treating several disorders, including depression, is becoming increasingly popular in western societies. The enzyme monoamine oxidase A (MAO-A) is one of the targets of the antidepressant drugs available in the market. Since almost 30% of the patients do not respond to the current treatments it is urgent to find new antidepressants (Caraci, F., *et al.* *Eur. J. Pharmacol*. 2010, *626*, 64–71). Herbal teas prepared from several plant species with claimed antidepressant properties, as well as from other species non-related with depression treatment, have been screened for the first time against MAO-A by our group, with promising results. The following IC_50_ values were obtained: 699.8 μg/mL for *Jasminum officinalis* L. (Ferreres, F., *et al.* *J. Pharm. Biomed. Anal.* 2014, *88*, 157–161), 19.3 μg/mL for *Annona muricata* L., 40.5 μg/mL for *Hyssopus officinalis* L., 428.1 μg/mL for *Cereus grandiflorum* L. (Grosso, C., *et al.* *Microchem. J.* 2015, *119*, 176–182), 38.5 μg/mL for *Cochlospermum angolensis* Welw. (Ferreres, F., *et al.* *Phytochem.* *Anal*. 2013, *24*, 534–540), 17.4 μg/mL for *Jacaranda caroba* (Vell.) A. DC. (Ferreres, F*., et al.* *Food Chem. Toxicol.* 2013, *57*, 91–98) and 99.5 μg/mL for *Grindelia robusta* Nutt. (Ferreres, F., *et al.* *J. Pharm. Biomed. Anal*. 2014, *94*, 163–172).

The herbal teas were also analysed by HPLC-DAD-ESI-MS^n^ and HPLC-DAD, revealing the presence of different hydroxybenzoic acids, hydroxycinnamic acids and flavonoids.

Since *A. muricata* displayed a strong anti-MAO-A activity and is traditionally used against depression, its herbal tea was selected to be further incorporated in liposomes functionalized with ApoE. From the chemical point of view, the extract is composed by 5-*O*-caffeoylquinic acid, quercetin-3-*O*-galactoside, quercetin-3-*O*-glucoside, quercetin-3-*O*-rutinoside and kaempferol-3-*O*-rutinoside.

Further studies will include the assessment of the effect of these herbal teas on serotonin and/or noradrenaline reuptake transporters, which are also extremely important targets for antidepressant drug design.

**Acknowledgments:** This work received financial support from the European Union (FEDER funds through COMPETE) and National Funds (Fundação para a Ciência e a Tecnologia, FCT) through the project UID/QUI/50006/2013. C. Grosso thanks FCT for the FCT Investigator (IF/01332/2014).

### 3.10. SLMP53-1: A New Reactivator of Mutant p53 with Potent in Vivo Antitumor Activity (MC17)

Joana Soares ^1^, Liliana Raimundo ^1^, Nuno A.L. Pereira ^2^, Ângelo Monteiro ^2^, Sara Gomes ^1^, Cláudia Bessa ^1^, Ana S. Gomes ^1^, Célia Gomes ^3^, Flávio Reis ^3^, Clara Pereira ^1^, Maria M.M. Santos ^2,^* and Lucília Saraiva ^1,^*

^1^ UCIBIO/REQUIMTE, Laboratório de Microbiologia, Departamento de Ciências Biológicas, Faculdade de Farmácia, R. Jorge Viterbo Ferreira, 228, 4050-313 Porto, Portugal

^2^ Research Institute for Medicines (iMed.ULisboa), Faculdade de Farmácia, Universidade de Lisboa, Portugal

^3^ Laboratório de Farmacologia e Terapêutica Experimental, IBILI, Faculdade de Medicina, Universidade de Coimbra, 3004-548 Coimbra, Portugal

* Correspondence: lucilia.saraiva@ff.up.pt (M.M.M.S.); mariasantos@ff.ulisboa.pt (L.S.)

The p53 tumor suppressor is a transcription factor inactivated in all human cancers due to mutations in the p53 protein or to the overexpression of endogenous negative regulators of wild-type (wt) p53. The high prevalence, and the often observed increased drug resistance of mutant p53-expressing tumors, make mutant p53 a highly appealing target for novel anticancer therapies (Bykov, V.J.N., *et al.* *FEBS Lett.* 2014, *588*, 2622–2627). In the present work, yeast assays consisting of *Saccharomyces cerevisiae* cells expressing human wt p53 or the most prevalent human mutant p53 forms were used to screen for reactivators of these inactive forms of p53. Using this approach, a chemical library of enantiopure tryptophanol-derived oxazoloisoindolinones was tested, and **SLMP53-1** was selected as a potential activator of wt p53 and reactivator of mutant p53R280K (Soares, J., *et al.* 2014, WO2014/207688A1).

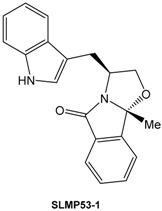


The molecular mechanism of **SLMP53-1** was further validated in human colon carcinoma tumor cells with wt p53 (HCT116 p53^+/+^) and in its p53-null isogenic derivative (HCT116 p53^−/−^), as well as in breast adenocarcinoma MDA-MB-231 cells expressing the mutant p53R280K. In these cells, **SLMP53-1** exhibited a p53-dependent growth inhibitory effect associated with G1-phase cell cycle arrest (in HCT116 p53^+/+^ cells) and apoptosis (in HCT116 p53^+/+^ and MDA-MB-231 cells), and increased the expression levels of several p53 target genes in HCT116 p53^+/+^ and MDA-MB-231 cells, but not in HCT116 p53^−/−^ cells. In MDA-MB-231 cells, **SLMP53-1** reestablished the wt-like DNA binding ability to mutant p53R280K. Additionally, **SLMP53-1** potently triggered a mitochondrial apoptotic pathway in HCT116 p53^+/+^ and MDA-MB-231 cells, involving Bax and wt/mutant p53 translocation to mitochondria. Besides this, **SLMP53-1** sensitized HCT116 p53^+/+^ and MDA-MB-231 cells to the effects of conventional chemotherapeutic agents and inhibited cell migration. Contrary to the majority of known p53 activators, no genotoxicity and *in vivo* toxicity were observed with **SLMP53-1**. Finally, the p53-dependent antitumor activity of **SLMP53-1** were validated *in vivo* using xenograft mouse models (Soares, J., *et al.* *Oncotarget* 2015, *7,* 4326–4343).

Collectively, besides its potential as anticancer drug, **SLMP53-1** belongs to a new chemical family, and its scaffold is a starting point for the development of effective drugs targeting mutant p53 forms.

### 3.11. Antimalarial Activity of s-Triazine Based Hybrids in Both Erythrocytic and Liver Stages (MC19)

Catarina A.B. Rodrigues ^1,^*, Raquel F.M. Frade ^1^, Inês S. Albuquerque ^2^, Maria J. Perry ^1^, Jiri Gut ^3^, Marta Machado ^2^, Philip J. Rosenthal ^3^, Miguel Prudêncio ^2^, Carlos A.M. Afonso ^1^ and Rui Moreira ^1^

^1^ iMed.ULisboa, Faculty of Pharmacy, University of Lisbon, Av. Professor Gama Pinto, 1649-003 Lisboa, Portugal

^2^ Unidade de Malária, Instituto de Medicinal Molecular, Faculty of Medicine, University of Lisbon, Av. Egas Moniz, 1649-028,Lisbon, Portugal

^3^ Department of Medicine, San Francisco General Hospital, University of California, P.O. Box 0811, San Francisco, CA 94143, USA

* Correspondence: catarinarodrigues@ff.ulisboa.pt

Malaria is a deadly disease that, despite being preventable and curable, is threatening the world wide health. Due to the fast resistance acquired by the Plasmodium parasite to the new developed drugs, an efficient molecule in both liver and blood stages was not yet established. (Dechy-Cabaret, O., *et al.* *J. Med. Chem.* 2012, *55*, 10328–10344). The combination of active structures that act by different mechanisms in one single molecule (Meunier, B., *Acc. Chem. Res.* 2008, *41*, 69–77) is a commonly used strategy to circumvent this inefficiency drawback. *s-*Triazine is a versatile core widely applied in the synthesis of hybrids with antimalarial activity, namely 4-aminoquinoline-s-triazine (Kumar, A., *et al.* *Eur. J. Med. Chem.* 2011, *46*, 676–690).

Primaquine presents the highest activity in liver stage and is also the only registered drug for radical cure of blood and liver stages malaria caused by *P. vivax* and *P.ovale* infection (Vale, N., *et al.* *Eur. J. Med. Chem.* 2009, *44*, 937–953).

Herein is presented the study of *s-*triazine hybrids with the liver stage active primaquine, aiming to find a molecule active in both liver and blood stage of malaria disease. *In vitro* tests in blood stage against *P. Falciparum W2* strain have shown encouraging results, with IC_50_ ranging from 0.2 to 8.3 microM. One primaquine-*s*-triazine hybrid showed very promising results in *in vitro* human hepatoma Huh-7 cells infected with a *P. berghei* line at a 1 microM dose.

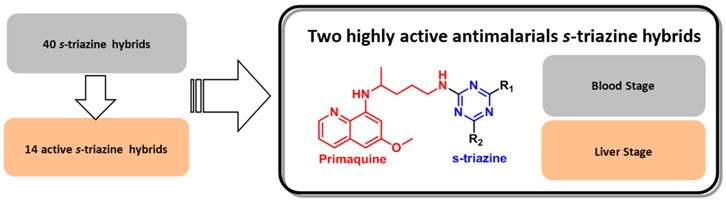


### 3.12. Wrapping it All Around: in Silico Approaches to Improve the MDR-Reversal Properties of the Macrocyclic Diterpenic Core (MC22)

Ricardo J. Ferreira ^1,^*, Rafael Baptista ^1^, Daniel J.V.A. dos Santos ^1,2,^*, Miguel X. Fernandes ^3^ and Maria-José U. Ferreira ^1^

^1^ Research Institute for Medicines (iMed.ULisboa), Faculty of Pharmacy, Universidade de Lisboa, Av. Gama Pinto, 1649-003 Lisboa, Portugal

^2^ REQUIMTE, Department of Chemistry and Biochemistry, Faculty of Sciences, University of Porto, Rua do Campo Alegre, 4169-007 Porto, Portugal

^3^ Instituto Universitario de Tecnologías Biomédicas (ITB), Universidad de La Laguna, 38200 La Laguna, España

* Correspondence: rjdgferreira@ff.ulisboa.pt, dsantos@ff.ulisboa.pt

The increasing number of chemotherapy failures reported worldwide identifies multidrug resistance (MDR) to anticancer drugs as a serious health concern. Since the over-expression of ABC transporters in cancer cell lines is one of the most reported MDR mechanisms, the inhibition of MDR-related efflux pumps as P-glycoprotein (Pgp) remains a promising approach for overcoming MDR (Eckford, P.D.W., *et al.* *Chem. Rev.* 2009, *109*, 2989).

Macrocyclic diterpenes isolated from *Euphorbia* species have been characterized as potent Pgp efflux modulators. However, their potency may be further improved by molecular manipulation of the diterpenic core. Thus, *in silico* approaches can be valuable tools for the identification of the most suitable modifications for MDR modulation (Ferreira, M.J.U., *et al.* *Phytochem. Rev.* 2014, *13*, 915).

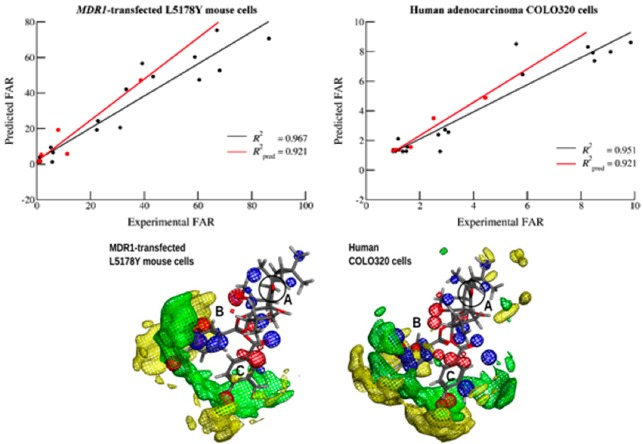


Several computational approaches were applied to a small diterpenic library (*n* = 25) obtained by chemical derivatization of compounds isolated from *Euphorbia boetica* (Matos, A.M., *et al.* *J. Nat. Prod.* 2015, *78*, 2215). A virtual screening procedure involving pharmacophoric identification followed by molecular docking was used to select and predict experimentally active MDR-reversal molecules (Ferreira, R.J., *et al. J. Chem. Theory Comput.* 2012, *8*, 1853; Ferreira, R.J., *et al. J. Chem. Inf. Model.* 2013, *53*, 1747), while ligand-based drug discovery techniques as quantitative structure-activity relationship (QSAR, Weka software) and pharmacophore modeling (calculated from molecular interaction fields with Open3DQSAR) were used to characterize the relationship between chemical modifications and the respective modulation capabilities (Baptista R., *et al.,* *Future Med. Chem.* 2015, *submitted*). From these procedures, a thorough characterization of the groups involved in the MDR-reversal activity was obtained, which can be further used to guide chemical derivatization, hopefully avoiding the synthesis of low-activity compounds.

### 3.13. The Anti-HIV Drug Rilpivirine: Covalent Adducts with Amino Acids and Proteins (MC23)

João P. Nunes ^1^, João Morais ^1^, Sofia A. Pereira ^2^, Cristina C. Jacob ^1^, Maria Matilde Marques ^1^ and Alexandra M.M. Antunes ^1,^*

^1^ CQE-IST, Centro de Química Estrutural, Instituto Superior Técnico, Universidade de Lisboa, 1049-001 Lisboa, Portugal

^2^ Centro de Estudos de Doenças Crónicas (CEDOC), NOVA Medical School, Universidade NOVA, 1169-056 Lisboa, Portugal

* Correspondence: alexandra.antunes@tecnico.ulisboa.pt

Rilpivirine (RPV) is a 2nd-generation non-nucleoside reverse transcriptase inhibitor (NNRTI) that was added to the available therapeutic options with the aim to overcome the most common adverse effects of the 1st-generation NNRTIs as well as their viral cross-resistance. However, post-marketing reports of RPV-associated depressive disorders are a cause for concern when contemplating chronic administration regimens. Therefore the development of reliable prognostic tools for pharmacovigilance procedures is urgent.

Covalent adduct formation with cysteine residues of synaptic proteins is considered a major mechanism of neurotoxicity induced by chemical toxicants such as acrylamide (LoPachin, R.M., *et al.* *Environ. Health Perspect*. 2012, *120*, 1650–1657). The identification of urinary Phase II conjugates stemming from initial 1,4 Michael addition of glutathione (GSH) to the α,β-unsaturated system of RPV supports the likelihood of reaction with proteins *in vivo (*Pereira, S.A., *et al. Adv. Mol. Toxicol.* 2012, *6*, 1–40).

With the ultimate goals of disclosing the mechanisms underlying RPV-induced neurotoxicity and developing suitable biomarkers of toxicity, the reactivity of RPV towards amino acids (*N*-acetyl-lysine and *N*-acetyl-cysteine) and model proteins such as Human Serum Albumin (HSA) was investigated by liquid chromatography—mass spectrometry (LC-MS) methodologies. The results obtained suggest the key role of RPV-derived covalent adduct formation in the onset of the adverse effects induced by this 2nd generation NNRTI.

**Acknowledgments:** This work was supported in part by Fundação para a Ciência e a Tecnologia (FCT), Portugal (RECI/QEQ-MED/0330/2012, UID/QUI/00100/2013 and IF/01091/2013/CP1163/CT0001). AMM also acknowledges Programa Operacional Potencial Humano from FCT and the European Social Fund (IF/01091/2013), and the LRI Innovative Science Award. We thank the Portuguese MS network (IST node) for providing access to the MS facility.

### 3.14. Interaction of Xanthone with Double Stranded DNA—A Contribution for Xanthone Derivative Drugs (MC24)

José Caetano * and M. J. Sottomayor

Department of Chemistry and Biochemistry, Faculty of Sciences, University of Porto, Rua do Campo Alegre, 4169-007 Porto, Portugal

* Correspondence: up201202547@fc.up.pt

Xanthones are an important group of oxygenated heterocyclic compounds, which are known to exhibit interesting pharmacological properties, such as anti-tumoral activity. Numerous studies have revealed that DNA is a primary intracellular target of anticancer drugs, due to the interaction of small molecules with DNA. Hence, characterization of the interaction of xanthones with DNA can be an important contribution for the development of a new class of anti-cancer agents.

In this communication we report the study of the interaction of xanthone with double stranded DNA, using UV-vis spectroscopy, including UV melting experiments, and viscosity measurements. The denaturation temperature and the thermodynamic parameters of DNA thermal denaturation were obtained from the curves of melted base pairs as a function of temperature. The binding constant of the xanthone–DNA complex, at 293 K, was calculated from the UV spectra.

The results indicate a strong binding affinity of xanthone with DNA, affecting the stability of the double helix, and suggest the binding of xanthone to DNA mainly by intercalation.

### 3.15. Synthesis of Phenolic Compounds Sulfate Metabolites (MC25)

Fátima Paiva-Martins *, Vânia Gomes, Carmen Torres, Susana Calçada and José Enrique Rodrígues-Borges

REQUIMTE, Departamento de Química e Bioquímica, Faculdade de Ciências, Universidade do Porto, Rua do Campo Alegre, s/n, 4169-007 Porto, Portugal

* Correspondence: mpmartin@fc.up.pt

The biological properties of olive oil polyphenols *in vivo* depend on the extent to which they are absorbed and metabolized. In a recent work, the metabolites hydroxytyrosol (3,4-dihydroxyphenylethanol) sulfate and hydroxytyrosol acetate sulfate were found to be the most useful metabolites for monitoring the intake compliance of extra virgin olive oil (Rubió, L., *et al.* *Food Res. Int.* 2014, *65*, 59–68).

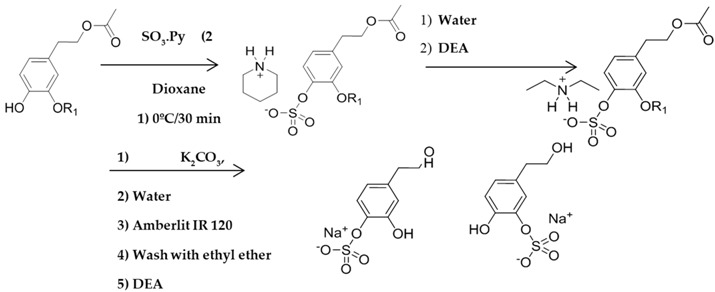


The growing interest in the bioactivity of natural polyphenols requires their metabolites to be used in bioassays and as standards in research protocols. Therefore, we report here the synthesis of several polyphenol sulfates namely hydroxytyrosol, hydroxytyrosol acetate, homovanillyl alcohol, homovanillyl alcohol acetate, homovanillilic acid, ferulic acid, and 3,4-dihydroxyphenylethanoic acid sulfates. A relatively fast and cheap synthetic solution based on avoidance of high temperature conditions during the synthesis and of low pressure conditions during purification has been established. Compounds were efficiently synthesized in 1–2 steps in a good yield (>75%).

### 3.16. Flavonoids Effects in Proinflammatory Signaling Systems: In Vitro Structure/Activity Studies (MC27)

Daniela Ribeiro ^1,^*, Marisa Freitas ^1^, Sara Tomé ^2^, Artur Silva ^2^ and Eduarda Fernandes ^1^

^1^ UCIBIO-REQUIMTE, Department of Chemical Sciences, Faculty of Pharmacy, University of Porto, 4050-313 Oporto, Portugal

^2^ Department of Chemistry & QOPNA, University of Aveiro, 3810-193 Aveiro, Portugal

* Correspondence: dsribeiro@ff.up.pt

Flavonoids have been associated with various health benefits, in which their anti-inflammatory effects play an important role. These properties associated with their ubiquitous distribution in nature, and their presence in the great majority of foods, as part of our daily diet, confer flavonoids great value-added molecules. Taking into account the potential anti-inflammatory properties, flavonoids started to be considered a valuable alternative to modulate and prevent inflammatory processes; and moreover, to be the base for the synthesis of more potent and efficient anti-inflammatory drugs (Ribeiro, D., *et al.* *Med. Res. Rev.* 2015, *35*, 877). The work herein developed intended to extend and rationalize the current knowledge on the alleged anti-inflammatory properties of flavonoids by elucidating the mechanism of action related with their structure (structure-activity relationship). For this purpose, a group of 24 flavonoids were selected, belonging to three flavonoid classes, flavones, flavanones and flavonols.

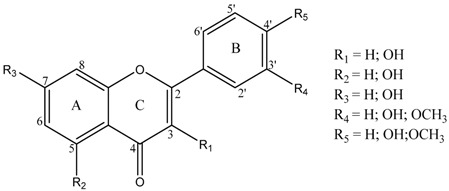


These flavonoids’ ability to modulate various proinflammatory signaling systems was assessed using various approaches: modulation of human neutrophils’ oxidative burst; inhibition of leukotriene B_4_, via 5-lipoxygenase, and prostaglandin _E2_, via cyclooxygenases -1 and -2, production; and apoptosis induction. The experimental studies performed in the scope of this work allowed the conclusion that among the tested flavonoids, the ones with a catechol group in B-ring revealed overall best anti-inflammatory activities. Indeed, flavonoids 3′,4′-dihydroxyflavone, 5,3′,4′-trihydroxyflavone, 7,3′,4′-trihydroxyflavone, and luteolin are undoubtedly good modulators of all the proinflammatory mediators evaluated, constituting promising alternatives for the resolution of inflammatory processes.

**Acknowledgments:** We would like to thank FCT (Fundação para a Ciência e a Tecnologia) for the financial support to UCIBIO-REQUIMTE (project: UID/Multi/04378/2013), trough National and European Union Funds, and to thank University of Aveiro and FCT/MEC for the financial support to the QOPNA research Unit (FCT UID/QUI/00062/2013), through national founds and where applicable co-financed by the FEDER, within the PT2020 Partnership Agreement. Daniela Ribeiro and Marisa Freitas acknowledge FCT for the financial support to their Ph.D. grant (SFRH/BD/72966/2010) and Pos-doc grant (SFRH/BPD/76909/2011), respectively, in the ambit of “QREN—POPH—Tipologia 4.1—Formação Avançada”, co-sponsored by FSE and by national funds of MCTES.

### 3.17. Analysis of Characteristics Mannitol (C_6_H_14_O_6_) Used in Bags of Red Cell Concentrates (MC28)

Diego M. Ferreira *, Joel A. Rocha Filho, Estela R. Ramos Figueira and Cristiany Barros Ludwig

LIM/37—Laboratório de Transplante e Cirurgia de Fígado da Faculdade de Medicina da Universidade de São Paulo—FMUSP, Av. Arnaldo, 455—Cerqueira César, CEP: 01246903 São Paulo, Brasil

* Correspondence: diegoimeil@hotmail.com

The oxygen supply to the cells need meet the metabolic demand, and have a critical effect in energy requirement in the cardiorespiratory system, this offer can be seriously affected in the case a person suffering a injury because of a surgery trauma or car accident causing blood loss, in the cases it is normal doctors recommend the transfusion of concentrates of red blood cells to help O_2_ supply maintenance in the human organism. The red blood cells (CH) have a complex system (Razouk, F.H., *et al.* *Rev. Bras. Hematol. Hemoter*. 2004, *26*, 126–134) having three different stages of C = 3 substances (plasma, proteins and oxygen) being multiphase P + 3 (solid, liquid and gas). For -CH be stored is necessary that it be carried out at temperatures 2 °C to 6 °C, depending on how this occurs can lead to formation of ice crystals, causing damage to the red blood cells. Depending on the severity of the damage caused during the cryopreservation process can lead to cell death. To reduce effect caused because formation of ice crystals in the blood component, in the storage process is added cryoprotectant solutions such as mannitol (C_6_H_14_O_6_), in the bags blood derived bags (Drozdov, A. D., *Mech. Res. Commun.* 1996, *23*, 543–548). Besides the natural toxicity caused by the use of cryoprotectants agents in the cryopreservation process, also observed the formation of reactive oxygen species (ROS), and in the case of mannitol (C_6_H_14_O_6_) subject to the following reaction:

2 C_6_H_14_O_6_ + 13 O_2_ ⇌ 12 CO_2_ + 14 H_2_O

The use of mannitol (C_6_H_14_O_6_), leads to the consumption of oxygen released by molecules of hemoglobin contained in the concentrated bags of red cell—CH, leading to decreased efficacy of oxygen transport, since only 50% of Hemogloina-Hb to P50 is covers carry molecules of O_2_, also taking into account that the human body at room temperature is covers to extract only 30% of O_2_ Hb, and this forms an efficiency of only 15% extraction of the total of all a bag CH hemacia- concentrate^3,4^. Thus the addition of mannitol (C_6_H_14_O_6_) as cryoprotectants ultimately further reduce the effectiveness of blood transfusion and its blood products.

### 3.18. Synthesis and Reactivity of 2-Methyl-Azolium Derivatives (OC1)

Margarida Figueiredo *, Luísa M. Ferreira and Paula S. Branco

LAQV@REQUIMTE, Departamento de Química, Faculdade de Ciências e Tecnologia, Universidade Nova de Lisboa, 2829-516 Caparica, Portugal

* Correspondence: mg.figueiredo@campus.fct.unl.pt

The reactivity of imidazolium salts has been thoroughly studied since its derivatives have shown to have numerous applications (Sowmiah, S., *et al.* *Molecules* 2009, *14*, 3780–3813; Feroci, M., *Electrochim. Acta* 2015, *153*, 122–129). Nevertheless, very little has been published concerning the reactivity when functionalized at the C2 position (Bhatnagar, A., *et al.* *Int. J. PharmTech Res*. 2011, 268–282), although C2-derivatives of azoles has shown to be associated with some relevant biological properties of azoles. Therefore, the reactivity of 2-methyl-imidazole derived salts was studied, in the presence of imines and aromatic aldehydes.

When imidazolium chloride (**1)** reacted with *N*-arylsulfonilimines (**2a**–**d)** in the presence of a base, arylethyl-2-imidazolium-1-tosilamides (**3a**–**d)** were obtained. Although the transient identification of the addition product **4c** was detected on the reaction with aromatic aldehydes, the major product in the reaction was benzoic acid (**4a**) and 3-ethyl-1,2-dimethyl-1H-imidazol-3-ium benzoate (**4b**). This work is still ongoing, for further mechanism elucidation.

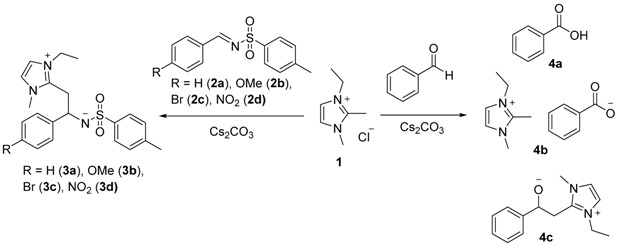


### 3.19. Recent Developments in the Synthesis of Novel Xanthone-1,2,3-triazole Dyads (OC2)

Hélio Albuquerque ^1,^*, Clementina Santos ^1,2^, José Cavaleiro ^1^ and Artur Silva ^1^

^1^ Department of Chemistry & QOPNA, University of Aveiro, 3810-193 Aveiro, Portugal

^2^ School of Agriculture, Polytechnic Institute of Bragança, Campus de Santa Apolónia, 5301-855 Bragança, Portugal

* Correspondence: helio.albuquerque@ua.pt

The development of multi-target drugs for treating complex multifactorial diseases constitutes an active research field. This kind of drugs has gained much importance as alternative strategy to combination therapy (“cocktail drugs”) (Nepali, K., *et al. Eur. J. Med. Chem.* 2014, *77*, 422–487). A common way to design them brings together two different pharmacophores in one single molecule (so-called dyads). Following this idea and being aware that xanthones (Pinto, M. M., *et al.* *Curr. Med. Chem.*. 2005, *12*, 2517–2538) and 1,2,3-triazoles (Lauria, A., *et al. Eur. J. Org. Chem*. 2014, 3289–3306) possess important pharmacological properties, we combined these two heterocycles in one molecule to create new dyads with improved therapeutic potential. In this work, new xanthone-1,2,3-triazole dyads were prepared from novel (*E*)-2-(4-arylbut-1-en-3-yn-1-yl)chromones by two different approaches to evaluate their efficiency and sustainability. Both methodologies involved Diels-Alder reactions to build the xanthone core, which were optimized using microwave irradiation as alternative heating method, and 1,3-dipolar cycloadditions to insert the 1,2,3-triazole moiety (Albuquerque, H.M.T., *et al. Eur. J. Org. Chem*. 2015, 4732–4743). All final and intermediate compounds were fully characterized by 1D and 2D NMR techniques.

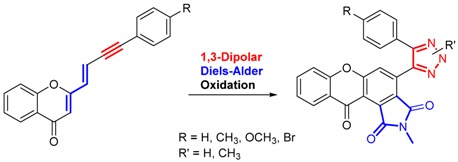


### 3.20. Sustainable Synthesis of Dihydropyrimidine-2(1H)-thiones under Mechanical Action (OC4)

Marta Pineiro ^1,^*, Cristina M. Chaves ^1^, Carla Gomes ^1^, Joana Quaresma ^2^ and José Campos ^2^

^1^ Coimbra Chemsitry Center, Department of Chemsitry, FCT-University of Coimbra, Rua Larga, 3004-535 Coimbra, Portugal

^2^ LDAP, Mechanical Engineering Department, FCT-University of Coimbra, Polo II, 3030-790 Coimbra, Portugal

* Correspondence: mpineiro@qui.uc.pt

Chalcones and dihydropyrimidine-2(1*H*)-thiones are biologically active compounds that have shown rather diverse pharmacological properties, such as antiviral, antiparasitic and anticancer activities (Kappe, C.O., *Eur. J. Med. Chem.* 2000, *35*, 1043–1052). Beyond the classic Biginelli reaction, within the last decade, there are very few examples in the literature describing the synthesis of 3,4-dihydropyrimidin-2(1*H*)-ones or thiones carrying no substituents at the 5 or 6 positions of the heterocycle. Organic chemists have been looking for more sustainable methodologies and ways to improve de greenness of organic synthesis. Exclude solvent from the reaction medium, generally the main source of waste in a synthetic process, is probably the most efficient way to attain this objective. Mechanical activation (MA), which is normally carried out in the absence of, or with minimal use of solvents, could be and alternative to improve the sustainabiliaty of organic synthesis (Friscic, T., *Chem. Soc. Rev.*. 2012, *41*, 3493–3510; James, S.L., *et al* *Chem. Soc. Rev.* 2012, *41*, 413–447). Herein we present the synthesis of chalcones and 4,6-diaryldihydropyrimidine-2(1*H*)-thiones in short reaction times and high yields using automatized mechanical action. The improvement of the sustainability of this method, compared with the use of conventional methodologies, was assessed by E-factor values under 10 and EcoScale values above 70.

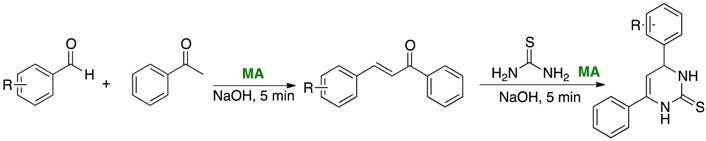


**Acknowledgments:** The authors thank FCT (Coimbra Chemistry Centre, UID/QUI/00313/2013) for financial support and the UC-NMR facility for NMR spectroscopic data (www.nmrccc.uc.pt).

### 3.21. Exploring the Reactivity of Novel Tetrazol-5-yl-Allenes for the Synthesis of Tetrazolyl-Heterocycles (OC5)

Ana L. Cardoso * and Teresa M. V. D. Pinho e Melo

Department of Chemistry, University of Coimbra, 3004-535 Coimbra, Portugal

* Correspondence: analcclopes@gmail.com

We have previously reported the reactivity of allenoates towards aziridines in organic solvents (Pinho e Melo, T.M.V.D., *et al.* *Tetrahedron* 2010, *66*, 8815–8822) and in supercritical carbon dioxide (Pinho e Melo, T.M.V.D., *et al.* *Synthesis* 2011, *21*, 3516–3522). Recently, we become interested in developing new synthetic routes to 5-substituted-1*H*-tetrazoles which are used in medicinal chemistry as bioisosteres of carboxylic acids^3^ (Pinho e Melo, T.M.V.D., *et al.* *Eur.* *J. Org. Chem.* 2014, *24*, 5159–5165).

In this communication, we describe the synthesis of novel tetrazol-5-yl-allenes **2** and their reactivity towards aziridines **3** leading to the synthesis of functionalized methylenepyrrolidines **4** and pyrroles **5**. The Wittig reaction between the ylide, formed from the phosphonium chloride **1**, and ketenes gave the target allenes in high yields.

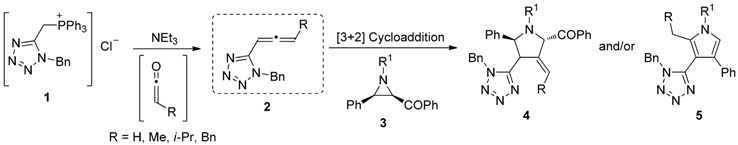


### 3.22. Chitobiose Modification: A Fast Forward Approach to Attain Relevant Disaccharides (OC6)

Luísa C.R. Carvalho * and Maria Manuel B. Marques

LAQV@REQUIMTE, Departamento de Química, Faculdade de Ciências e Tecnologia, Universidade Nova de Lisboa, Campus de Caparica, 2829-516 Caparica, Portugal

* Correspondence: luisa_carvalho@campus.fct.unl.pt

The biological importance of glycostructures made them popular targets in synthetic chemistry, in particular those incorporating *N*-acetyl-d-glucosamine (NAG) units. The urgent need of these compounds in pure form and in significant amount has implied vast synthetic efforts. Usually oligosaccharides are constructed through sugar monomers manipulation, which implies time-consuming protection/deprotection steps and wild glycosylations. Several approaches have been developed to attain complex glycostructures (Boltje, T.J., *et al.* *Nat. Chem*. 2009, 611–622). However, it has been demonstrated that glycosylation using NAG derivatives as glycosyl donors is still a difficult task (Zhu, X., *et al.* *Angew. Chem. Int. Ed*. 2009, *48*, 1900–1934). Our group has been involved in the synthesis of glycans containing NAG, specifically on the assembly of small fragments of bacterial peptidoglycan (Enugala, R., *et al. Synlett* 2010, *18*, 2711–2716; Enugala, R., *et al. Chem. Asian J*. 2012, *7*, 2482–2501; Enugala, R., *et al. Carbohydr. Res*. 2014, *384*, 112–118). Due to our interest in glycostructures involved in important biological mechanisms, it was envisaged the direct modification of chitobiose to attain the desired compounds.

**Acknowledgments:** The authors acknowledge to FCT for funding PTDC/QEQ-QOR/2132/2012.

### 3.23. The Search for New Antipsychotic Compounds Incorporating the N-methylpiperazine Nucleus (OC8)

Elina Marinho * and M. Fernanda Proença

Centre of Chemistry, Department of Chemistry, University of Minho, Campus de Gualtar, 4710-057 Braga, Portugal

* Correspondence: ElinaMarinho@sapo.pt

Clozapine, a molecule incorporating the *N*-methyl piperazine nucleus, is a gold standard among antipsychotic medications for schizophrenia. Its therapeutic use is restricted by agranulocytosis, a fatal blood disorder associated with the use of the drug (Lieberman, J.A., *et al.* *J. Clin. Psychiatry* 1988, *49*, 271–277). It is crucial to develop new molecules inspired in the clozapine scaffold, with potential antipsychotic activity and reduced side effects, capable of interacting with different receptors associated to this type of disorders, namely the 5-HT serotonine receptors (Ye, N., *et al.* *Chem. Rev*. 2013, *113*, 123–178).

In this work we describe the synthesis of a selection of compounds incorporating the piperazine unit in an amide linker associated with the aromatic nucleus. Different arylamines were reacted with maleic anhydride to generate amides. The alkene moiety selectively incorporated *N*-methyl piperazine, 5-bonds away from the aromatic fragment (compound **3**). Details on the synthesis of these compounds will be presented and also the biological activity at serotonine 5-HT_2A_ receptors.

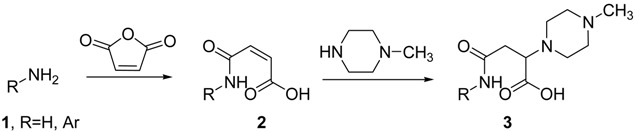


### 3.24. Strategies towards the Synthesis of New (E)-2-Aryl-3-styryl-4H-chromen-4-ones and (E)-2-Aryl-1-methyl-3-styrylquinolin-4(1H)-ones (OC9)

Diana C.G.A. Pinto *, Djenisa H.A. Rocha and Artur M.S. Silva

Department of Chemistry & QOPNA, University of Aveiro, 3810-193 Aveiro, Portugal

* Correspondence: diana@ua.pt

Flavones (2-aryl-4*H*-chromen-4-ones) and quinolones [2-arylquinolin-4(1*H*)-ones] are important classes of bioactive drug targets in the pharmaceutical industry, as they are the core structure of numerous biologically active compounds (Verma, A.K., *et al.* *Nat. Prod. Rep.* 2010, *27*, 1571–1593; Mitscher, L.A., *Chem. Rev.* 2005, *105*, 559–592). On the other hand the presence of a styryl group attached to a chromone core also seems to enhance the biological activity (Gomes, A., *et al. Mini Rev. Med. Chem.* 2010, *10*, 1–7). Taking these important aspects into consideration we have developed new and efficient routes towards the synthesis of 2-aryl-3-styryl-4*H*-chromen-4-ones and 1-methyl-2-aryl-3-styrylquinolin-4(1*H*)-ones **5** (Scheme). These routes include efficient one-pot methods and the use of Wittig and Heck type reactions (Rocha, D. H. A., *et al. Synlett* 2012, 559–564; Rocha, D.H.A., *et al. Synlett* 2013, 2683–2686; Rocha, D.H.A., *et al.* *Tetrahedron* 2015, *71*, 7717–7721).

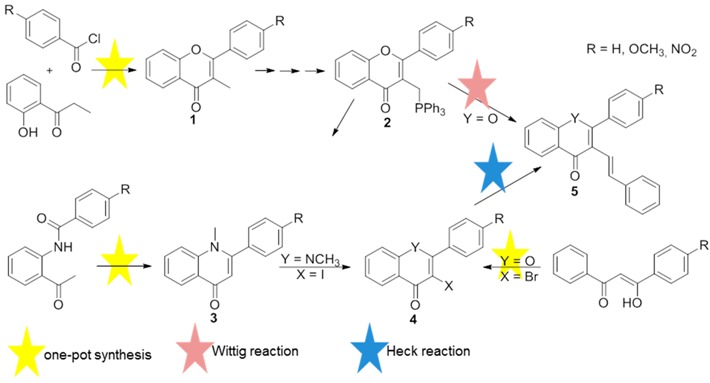


**Acknowledgments:** We would like to thank University of Aveiro and FCT/MEC for the financial support to the QOPNA research Unit (FCT UID/QUI/00062/2013), through national founds and where applicable co-financed by the FEDER, within the PT2020 Partnership Agreement, and also to the Portuguese NMR Network. D.H.A.R. also thanks FCT for her Ph.D. grant (SFRH/BD/68991/2010).

### 3.25. 3-Bromochromones as Building Blocks of Novel Furan and Cyclopropane Derivatives (OC11)

Joana Sousa ^1,^*, Oualid Talhi ^1,3^, Filipe Paz ^2^ and Artur Silva ^1^

^1^ QOPNA, Department of Chemistry, University of Aveiro, 3810-193 Aveiro, Portugal

^2^ CICECO—Aveiro Institute of Materials, Department of Chemistry, University of Aveiro, 3810-193 Aveiro, Portugal

^3^ Centre de Recherche Scientifique et Technique en Analyses Physico-Chimiques CRAPC, BP384, Bou-Ismail, 42004 Tipaza, Algeria

* Correspondence: joanasousa@ua.pt

The furan and cyclopropane rings can be found in a variety of biologically active synthetic and natural compounds (Luo, Y., *et al. Bioorg. Med. Chem. Lett.* 2015, *25*, 2421–2424; Sampson, P.B., *et al. J. Med. Chem.* 2015, *58*, 130–146). Due to their importance as potential pharmaceutical agents, the synthesis of novel furan- and cyclopropane-containing compounds is a highly active research field. For this purpose, 3-bromochromones arise as desirable and versatile starting materials (Sousa, J.L.C., *et al. Synlett* 2015, *26*, 2724–2729). Exploring the singular chemical features of such chromone derivatives, herein we present the synthesis of two different families of oxygen-containing heterocycles—furan-based polyphenolics and fused chromanone-cyclopropanes—starting from the same 3-bromochromones and using one-pot base-catalyzed reactions. Specifically, our methodologies relay on tandem reactions of 3-bromochromones **1** with 1,3-dicarbonyl compounds **2** to afford a series of furan-based polyphenolic derivatives **3**, and ketone compounds **4** to prepare a library of fused chromanone-cyclopropane derivatives **5** in the presence of organic and inorganic bases, respectively.
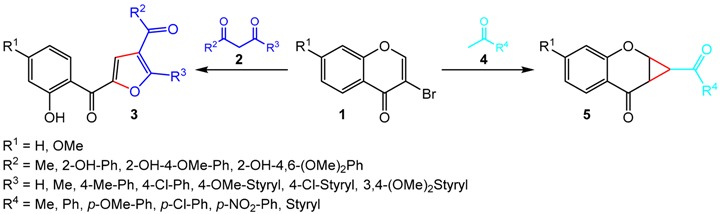


### 3.26. Synthesis of New Bis(indolyl)methanes With Anti-Cancer Activity (OC16)

Carla Grosso ^1,^*, Ana L. Cardoso ^1^, Américo Lemos ^2^ and Teresa M.V.D. Pinho e Melo ^1^

^1^ Department of Chemistry, University of Coimbra, 3004-535 Coimbra, Portugal

^2^ CIQA, University of Algarve, Campus Gambelas, 8005-139 Faro, Portugal

* Correspondence: carla_grosso@hotmail.com

We have recently disclosed an approach to novel bis(indolyl)methane oximes (BIM Oximes) *via* Hetero-Diels-Alder reactions of nitrosoalkenes with indoles. This class of compounds showed very interesting anti-cancer activity, in particular against leukaemia and lymphoma cell lines (Pinho e Melo, T., *et al. Eur. J. Med.* 2015, *93*, 9). In this communication, the tuning of the structure of scaffold **1** which led to the preparation of a range of new BIM oximes will be described.

Moreover, the one-pot synthetic strategy to BIMs was extended to novel bis(indolyl)methane hydrazones via bis-hetero-Diels-Alder reaction of azoalkenes with indoles. The biological evaluation of these new BIMs as anti-cancer agents will also be disclosed.

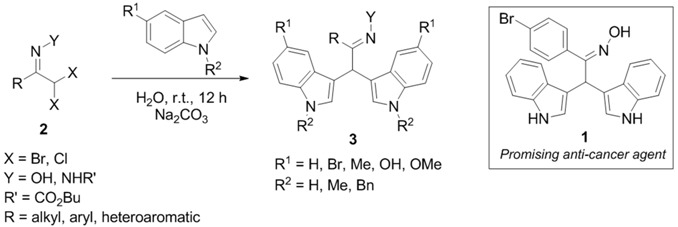


### 3.27. Unveiling the Chemistry of the Homemade Drug “Krokodil” (OC18)

João Neves ^1^, José Soares ^2^, Emanuele Alves ^3,5,^*, Sara Cravo ^1^, Artur Silva ^4^, Annibal Netto ^5^, Félix Carvalho ^6^, Ricardo Dinis-Oliveira ^3,6^ and Carlos Afonso ^1,7^

^1^ Department of Chemical Sciences, Laboratory of Organic and Pharmaceutical Chemistry, Faculty of Pharmacy, University of Porto, R. Jorge Viterbo Ferreira, 228, 4050-313 Porto, Portugal

^2^ LAQV-REQUIMTE, Department of Chemical Sciences, Laboratory of Applied Chemistry, Faculty of Pharmacy, University of Porto, R. Jorge Viterbo Ferreira, 228, 4050-313 Porto, Portugal

^3^ Department of Legal Medicine and Forensic Sciences, Faculty of Medicine, University of Porto, Al. Prof. Hernâni Monteiro, 4200 - 319 Porto, Portugal

^4^ Department of Chemistry and QOPNA, University of Aveiro, Campus Universitário de Santiago, 3810-193 Aveiro, Portugal

^5^ Department of Analytical Chemistry, Chemistry Institute, Fluminense Federal University, Niterói, RJ, 24220-900, Brazil

^6^ UCIBIO-REQUIMTE, Laboratory of Toxicology, Department of Biological Sciences, Faculty of Pharmacy, University of Porto, R. Jorge Viterbo Ferreira, 228, 4050-313 Porto, Portugal

^7^ Interdisciplinary Center of Marine and Environmental Investigation (CIIMAR/CIMAR), Rua dos Bragas 289, 4050-123 Porto, Portugal

* Correspondence: manuhpa@hotmail.com

“Krokodil” is a homemade substitute to heroin, which psychoactive substance is believed to be desomorphine **1**, an opioid ten times more potent than morphine. The users of this drug can develop severe skin ulcerations, and scale-like skin that resembles a crocodile, hence its street name. “Krokodil” is prepared at home with easily available materials and therefore its chemical composition is complex and still poorly understood (Alves, E.A., *et al. Forensic Sci. Int*. 2015, *249*, 207–213). Since its consume is spreading in Europe, it is important to elucidate and characterize “Krokodil” composition.

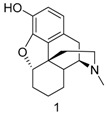


With the aim of studying the chemical composition of “Krokodil”, its street synthesis was mimicked resorting to the same materials and conditions used by street manufacturers. The chemical profile of “Krokodil” was outlined by chromatographic (HPLC and GC-MS) and spectroscopic techniques (IR, ^1^H-NMR, MS). With these data it was possible to establish the typical chromatographic and spectroscopic profiles of “Krokodil”.

**Acknowledgments:** This research was partially supported by the Strategic Funding UID/Multi/04423/2013 and PTDC/MAR-BIO/4694/2014 through national funds provided by FCT—Foundation for Science and Technology and European Regional Development Fund (ERDF), in the framework of the programme PT2020 and by Department of Chemical Sciences, Laboratory of Organic and Pharmaceutical Chemistry, Faculty of Pharmacy, University of Porto, Porto, Portugal; Conselho Nacional de Desenvolvimento Científico e Tecnológico (CNPq) (process 245844/2012-0) for research grants and scholarship.

### 3.28. UHPLC-QqQ-MS/MS Method for Phytoprostane Profiling in Macroalgae (OC20)

Mariana Barbosa ^1^, Fátima Fernandes ^1^, David M Pereira ^1^, Patrícia Valentão ^1^, Federico Ferreres ^2^, Ángel Gil-Izquierdo ^2^ and Paula B Andrade ^1,^*

^1^ REQUIMTE/LAQV, Laboratório de Farmacognosia, Departamento de Química, Faculdade de Farmácia, Universidade do Porto, Rua de Jorge Viterbo Ferreira, 228, 4050-313 Porto, Portugal

^2^ Research Group on Quality, Safety and Bioactivity of Plant Foods, Department of Food Science and Technology, CEBAS (CSIC), P.O. Box 164, Campus University Espinardo, 30100 Murcia, Spain

* Correspondence: pandrade@ff.up.pt

The analysis of phytoprostanes in natural matrices is extremely challenging, requiring highly sensitive and specific tools for their profiling and characterization. Moreover, the great diversity granted by the presence of racemic mixtures of phytoprostanes increases the complexity of those analyses (Barbosa, M., *et al. J. Agric. Food Chem.* 2015, *63*, 6466–6474).

Our work aimed at determining naturally occurring classes of free phytoprostanes in 24 macroalgae species belonging to Chlorophyta, Phaeophyta and Rhodophyta, collected along the western coast of Portugal and from integrated multi-trophic aquaculture (IMTA) systems. For this, a fast, selective, and robust ultrahigh-performance liquid chromatography coupled to triple-quadrupole mass spectrometry (UHPLC-QqQ-MS/MS) method was employed. Three classes of phytoprostanes were identified and quantitated for the first time in the analyzed species.

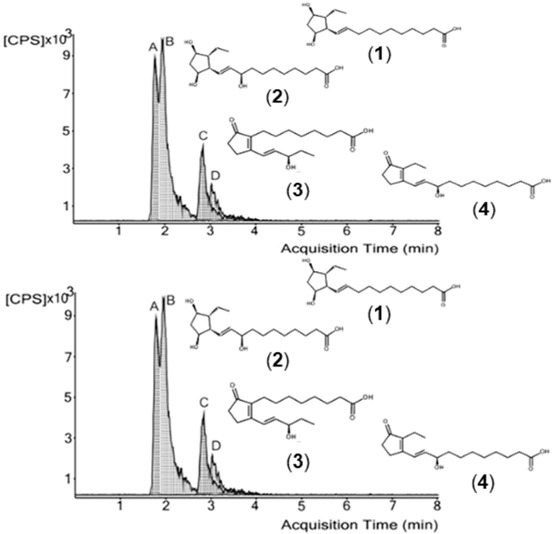

Representative UHPLC-QqQ-MS/MS chromatogram of detected phytoprostanes in *Codiumtomentosum* Stackhouse. (**1**) 9-F_1t_-phytoprostane, (**2**) 9-*epi*-9-F_1t_-phytoprostane, (**3**) 16-B_1_-phytoprostane, and (**4**) L_1_-phytoprostane.

The total phytoprostane content ranged between ca. 6 and 1381 ng/100 g of dry algae, F_1t_-phytoprostanes (**1** and **2**) and L_1_-phytoprostanes (**4**) constituting the major and minor classes, respectively.

Currently, the interest in phytoprostanes comprises two general areas: as biomarkers of oxidative stress in plant-derived foodstuffs and as bioactive mediators with potential benefits in human health. Therefore, the determination of phytoprostane levels in macroalgae is of extreme importance, encouraging the exploitation and characterization of new natural dietary sources of these compounds.

**Acknowledgments:** This work received financial support from the European Union (FEDER funds through COMPETE) and National Funds (FCT, Fundação para a Ciência e Tecnologia) through Project UID/QUI/50006/2013. The work also received financial support from Projects AGL2011-23690, AGL2013-45922-C2-1-R, and AGL2013-45922-C2-2-R (CICYT). We are greatly indebted to all financing sources. M.B. (SFRH/BD/95861/2013) and F.F. (SFRH/BPD/98732/2013) are indebted to FCT for their grants.

### 3.29. Change in Cognitive Effects Caused for Consumption of Caffeine—C_8_H_10_N_4_O_2_ (OC21)

Cristiany Barros Ludwig ^1,^* and Diego Mendes Ferreira ^2^

^1^ Departamento de Química da Escola Tecninca—Centro Paula Souza, Praça Adelaide B. Guedes, 01 Centro, Tatui, SP - CEP 18270-020, Brazil

^2^ LIM/37—Laboratório de Transplante e Cirurgia de Fígado da Faculdade de Medicina da Universidade de São Paulo, Avenida Doutor Arnaldo, 455 - Cerqueira César, São Paulo - SP, 01246-904, Brasil

* Correspondence: crisbarros.ludwig@outlook.com

There are times that humanity is daily consumption of substances ha basis of caffeine—C_8_H_10_N_4_O_2_ (teas, coffee and drugs) in their various forms of preparation. Studies indicate that daily consumption of caffeine causes improvements in memory capacity, however, ha indication of adverse consequences (Diukova, A, *et al. NeuroImage* 2012, *62*, 239–249), such as increased anxiety, among various other cognitive effects in humans and animals (Smit, H.J., *et al. Psychopharmacology* 2000, *152*, 167–173). After the analysis in two databases—Science Library Online (Scielo) and PubMed—using the criteria search “free full text” and “last five years”, we obtained the following data from the terms:
**Term****Full Articles****Articles Indicating Improvements****Articles Indicating Worsening****Articles That Don’t Point Difference**Memory131201Cognition13535Learning9342Anxiety11191Depression9081Stress5410

Although caffeine is consumed daily by a significant portion of the world’s population, yet there are few clinical studies that demonstrate the actual effects of it in humans and animals, which indicates a great need for experimental research in humans and animals based on consumption caffeine.

### 3.30. Bioactive Abietane Diterpenes from Plectranthus *spp.* Extracts and Its Encapsulation into a Novel Phytosomal Formulation (OC23)

Diogo Matias ^1,2^, Filipe Pereira ^1,2^, Marisa Nicolai ^1^, Maria Fátima Simões ^1^, Ana Maria Díaz-Lanza ^2^, Catarina Pinto Reis ^1,3^ and Patrícia Rijo ^1,4,^*

^1^ CBIOS, Universidade Lusófona de Humanidades e Tecnologias, Campo Grande 376, 1749-024 Lisbon, Portugal

^2^ Department of Biomedical Sciences, Faculty of Pharmacy, University of Alcalá, 28871 Alcalá de Henares, Spain

^3^ IBEB, Faculty of Sciences, University of Lisbon, 1749-016 Lisbon, Portugal

^4^ iMed.UL, Faculty of Pharmacy, University of Lisbon, Av. Gama Pinto, 1649-003 Lisbon, Portugal

* Correspondence: patricia.rijo@ulusofona.pt

*Plectranthus* plants (Lamiaceae) have diverse ethnopharmacological uses including gastro-intestinal, infections and skin conditions (Lukhoba C. W., *et al.* *J. Ethnopharmacol.* 2006, *103*, 1–24), which may be supported by the presence of bioactive abietane diterpenes (Burmistrova, O., *et al. Phytomedicine* 2013, *22*, 1009–1016). The intrinsic instability of these and their unfavorable partition coefficient, affects their bioavailability and therefore the bioactivity. New drug delivery systems as the Phytosome^®^, have proven to be effective in surpassing those limitations (Semalty, A., *et al.* *Fitoterapia* 2010, *81*, 306–314).

In this study, *P. madagascariensis* (PM), *P. neochilus* (PN) and *P. porcatus* (PP) extracts prepared using different methods and solvents, were screened for their bioactivities towards *Staphylococcus aureus* strains and MDA-MB-231 cells. PM extracts and their major components (HPLC-DAD) 7α,6β-dihydroxyroyleanone (**1**), 7α-acetoxy-6β-hydroxyroyleanone (**2**) and coleon U (**3**) showed antibacterial and cytotoxic activities (Scheme).

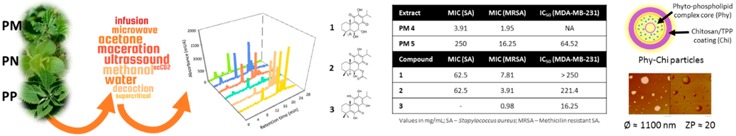


The most potent antibacterial extract (PM 4) was encapsulated into phytosomes microencapsulated with chitosan/TPP. The average size of the microspheres was 1082 ± 363 nm showing a low polydispersitivity index (0.224), zeta potential of 20.59 ± 12.02 mV and encapsulation efficiency of 57.70% ± 6.00%. Preliminary studies showed short-term stability, sustained release and antibacterial activity of the formulation. The results confirm the presence of bioactive compounds in *Plectranthus* extracts and usefulness of the encapsulation technology applied to improve the stability of bioactive extracts of PM.

### 3.31. ER Stress and Protein Quality Control Pathways: Exploring the Natural Products’ Chemical Space

David M. Pereira *, Patrícia Valentão and Paula B. Andrade

REQUIMTE/LAQV, Laboratório de Farmacognosia, Departamento de Ciências Químicas, Faculdade de Farmácia, Universidade do Porto, Rua de Jorge Viterbo Ferreira, 228, 4050-313 Porto, Portugal

* Author to whom correspondence should be addressed; dpereira@ff.up.pt

The endoplasmic reticulum (ER) is an organelle comprising a network of branching tubules and sacs that is present in all eukaryotic cells. ER stress has been identified as a hallmark, and sometimes trigger, of several pathologies, notably cancer, inflammation and neurodegenerative diseases such as Alzheimer’s and Parkinson’s.

One of the factors that can trigger ER stress is the presence of unfolded or misfolded proteins, which can be a consequence of the inhibition of the proteasome, a eukaryotic protein complex that is involved in proteolysis of undesired proteins.

Among the molecules described in literature known to affect ER and proteasome function, the majority are natural products, suggesting that natural molecules may constitute a significant arsenal of chemical entities for modulating this cellular target.

In this presentation, we will briefly outline current knowledge of ER biology and the hallmarks of ER stress, thus paving the way for presenting the natural products that have been described as being ER modulators, either stress inducers or ER protectors.

The chemistry, distribution and mechanism of action of these compounds will be presented and discussed, including examples both from the literature and from our laboratory.

## 4. Poster Presentations

### 4.1. CotA-Laccase as Biocatalyst in the “Green” Synthesis of Phenazine and Acridine Cores (P1)

Ana Catarina Sousa ^1,2^, Lígia O. Martins ^3^ and Maria Paula Robalo ^1,2,^*

^1^ Área Departamental de Engenharia Química, Instituto Superior de Engenharia de Lisboa, Rua Conselheiro Emídio Navarro, 1, 1959-007 Lisboa, Portugal

^2^ Centro de Química Estrutural, Complexo I, Instituto Superior Técnico, Av Rovisco Pais, 1049-001 Lisboa, Portugal

^3^ Instituto de Tecnologia Química e Biológica, Universidade Nova de Lisboa, Av. da República, 2780-15 Oeiras, Portugal

* Correspondence: mprobalo@deq.isel.ipl.pt

Phenazines and acridines are an important class of benzoheterocycle compounds which exhibit a broad spectrum of biological activities and a number of derivatives are widely used as antibacterial, antifungal, antiviral and anticancer drugs (Laursen, J.B., *et al.* *Chem. Rev.* 2004, *104*, 1663–1686; Lian, Y., *et al.* *J. Am. Chem. Soc.* 2013, *135*, 12548–12551). There are several chemical methods for the synthesis of these important cores starting from substituted *o*-aromatic amines that are also good substrates for laccases. Based on this property and seeking for a cleaner synthetic method, we present a “green” approach for the formation of symmetric and asymmetric phenazines and acridines using CotA-laccase, a multicopper oxidase, as biocatalyst. Laccase promote the oxidation of the precursor molecules to the *o*-quinone-diimine intermediates which undergo a Michael addition leading to the final heterocycle cores (Sousa, A.C., *et al.* *Green Chem.* 2014, *16*, 4127–4136). Reactions were performed in aqueous media and with mild reaction conditions of pH and temperature.

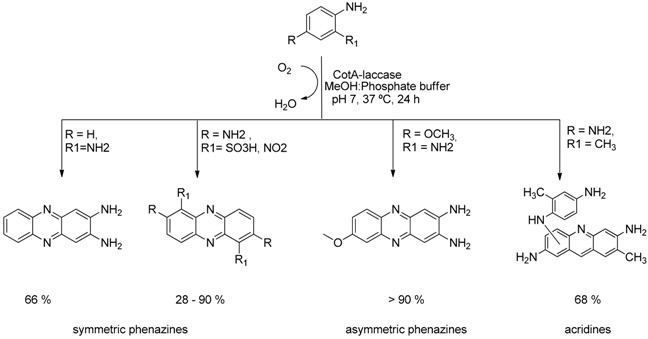


### 4.2. Impact of Citrus Pectin on Oenin Copigmentation Mechanisms (P2)

Ana Fernandes ^1,^*, Natércia Brás ^2^, Nuno Mateus ^1^ and Victor de Freitas ^1^

^1^ REQUIMTE\LAQV, Departamento de Química e Bioquímica, Rua do Campo Alegre, 4169-007 Porto, Portugal

^2^ REQUIMTE\UCIBIO, Departamento de Química e Bioquímica, Rua do Campo Alegre, 4169-007 Porto, Portugal

* Correspondence: ana.fernandes@fc.up.pt

Copigmentation is regarded as one mechanism for anthocyanin’s color stabilization (Boulton, R. *Am. J. Enol. Vitic*. 2001, *52*, 67–87). Also, interaction with polysaccharides is an important topic to be considered on anthocyanin’s stabilization, as anthocyanins are synthetized in a polysaccharide environment where extensive contact may occur (Padayachee, A., *et al.* *Food Chem*. 2012, *134*, 155–161). These associations may affect pigment’s stability and color. This work aimed to evaluate the impact of pectin on anthocyanin’s copigmentation mechanisms. Oenin copigmentation was observed in the presence of (+)-catechin, as indicated by an increase in the absorbance. However, the copigmentation binding constants (*K*_CP_) determined for the interaction of catechin with oenin in the presence of a pectic polysaccharide showed that this polysaccharide induced a decrease on the copigmentation binding constants. These results probably suggest the occurrence of competition equilibrium in which the presence of this pectic polysaccharide limited the association between catechin and oenin.

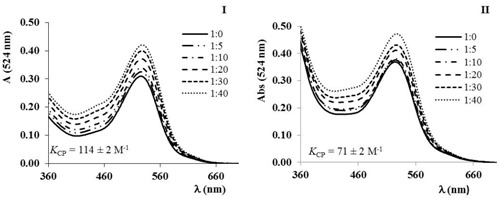


### 4.3. Morella Faya (Aiton) Wilbur Leaves and Bark: Bioactivities and Isolated Compounds (P3)

Bruno J. C. Silva ^1^, Maria Carmo Barreto ^1^, Artur M. S. Silva ^2^ and Ana M. L. Seca ^1,2,^*

^1^ DCTD, University of Azores, 9501-801 Ponta Delgada, Portugal

^2^ Department of Chemistry & QOPNA, University of Aveiro, 3810-193Aveiro, Portugal

* Correspondence: anaseca@ua.pt

*Morella faya* (Aiton) Wilbur (syn. *Myrica faya* (Aiton)), from Myricaceae family, is an evergreen nitrogen-fixing subdecious shrub or small tree native to Macaronesia (Lutzow-Felling, C.J., *et al.* Technical Report University of Hawaii (http://manoa.hawaii.edu/hpicesu/techr/094.pdf), 1995, *94*, 1–25). *Morella* species have traditional medicine uses and metabolites that exhibit promising bioactivities (Silva, J., *et al.* *Int. J. Mol. Sci.* 2015, *16*, 17160–17180). Here we present the biological activity of bark and leaves extracts from *M. faya* and some compounds isolated from these extracts.

The bark acetone extract presented a strong anti-acetylcholinesterase activity, comparable to the commercial drug galanthamine. Moreover, the inhibition was also reversible, which means that it will be less toxic than an irreversible inhibitor. Both acetone extracts presented comparable inhibition values against xanthine oxidase, with only approximately 4 to 5-fold the IC_50_ for standard drug allopurinol. Considering that these extracts were reversible inhibitors, it means that they have the potential to be less toxic than allopurinol, which is approved for medical purposes.

From the most active extracts were isolated fatty alcohol (**1**–**2**), pentacyclic triterpenes with lupane and oleanane skeleton (**3**–**6**), cyclic diarylheptanoid (**7**) and a phthalate (**8**). All these compounds are reported in *M. faya* for the first time and belong to organic families well known by the broad spectrum of biological activities exhibited by its members. These results show the potential of *M. faya* as medicinal plant and source of pharmacologically active compounds.

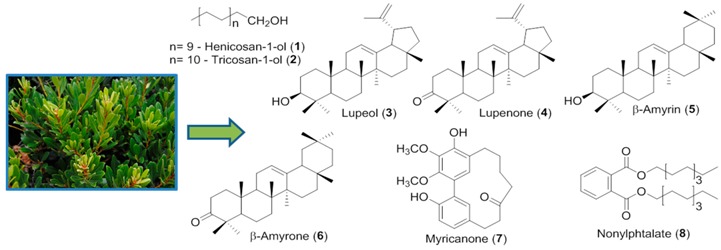


**Acknowledgments:** We would like to thank University of Aveiro and FCT/MEC for the financial support to the QOPNA research Unit (FCT UID/QUI/00062/2013), through national founds and where applicable co-financed by the FEDER, within the PT2020 Partnership Agreement, and the University of Azores.

### 4.4. Natural Products: Tools for Inflammation Management (P5)

Andreia P. Oliveira, Ivone Sá, Clara Grosso, David M. Pereira, Patrícia Valentão and Paula B. Andrade *

REQUIMTE/LAQV, Laboratório de Farmacognosia, Departamento de Química, Faculdade de Farmácia, Universidade do Porto, R. Jorge Viterbo Ferreira, 228, 4050-313 Porto, Portugal

* Correspondence: pandrade@ff.up.pt

The recurrent use of anti-inflammatory drugs and their side effects led to a growing demand for viable and safer alternatives. In this context, natural products arise, playing an important role in the treatment of this pathology. Amongst natural compounds with anti-inflammatory properties, flavonoids can be highlighted. In this work, the ability of *Grindelia robusta* Nutt aqueous extract and of some of its flavonoids, to reduce nitric oxide (NO) levels in RAW 264.7 cells was assessed. Results revealed that the extract of *G. robusta* reduced cells’ viability.

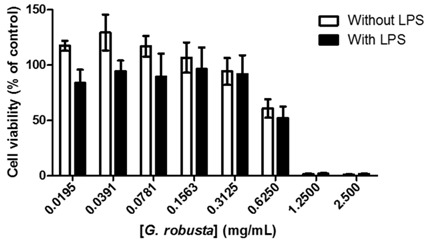


Cell viability of RAW 264.7 cells pre-treated for 2 h with *G. robusta* aqueous extract, followed by 22h co-treatment with LPS (1 μg/mL) with LPS or vehicle (culture medium). Results represent the mean ± standard deviation of four independent experiments performed in triplicate.

A tendency to reduce NO levels, in a dose-dependent way, was also observed. All flavonoids were able to decrease NO levels in a concentration-dependent manner, quercetin being the most effective one (IC_50_ values of 7.47 μM). The presence of quercetin, apigenin and luteolin derivatives in the extract of *G. robusta* can partially explain its capacity to decrease NO levels. In a general way, aglycones revealed to be more active than the respective glycosides. Furthermore, the catechol group on ring B and the hydroxyl group in C3 seem to be essential for the anti-inflammatory activity of these compounds.

**Acknowledgments:** This work received financial support from the European Union (FEDER funds through COMPETE) and National Funds (FCT, Fundação para a Ciência e Tecnologia) through project UID/QUI/50006/2013. To the financing sources, the authors are greatly indebted. Andreia P. Oliveira (SFRH/BPD/96819/2013) and C. Grosso (IF/01332/2014) are indebted to FCT for the grant and FCT Investigator, respectively.

### 4.5. Antitumor Activities of Invasive Alien Species from the Azores (P6)

Ana Bettencourt ^1^, Joana Micael ^2^, Ana Cristina Costa ^2^, Ana M. L. Seca ^1,3^ and Maria do Carmo Barreto ^1,^*

^1^ DCTD, Azores University, 9501-801 Ponta Delgada, Portugal

^2^ InBio, CIBIO-Açores / DB, Azores University, 9501-801 Ponta Delgada, Portugal

^3^ QOPNA, Universidade de Aveiro, 3810-193 Aveiro, Portugal

* Correspondence: maria.cr.barreto@uac.pt

*Asparagopsis armata* Harvey, *Asparagopsis taxiformis* (Delile) Trévisan Saint-Léon, *Codium fragile* subsp. *fragile* (Suringar) Hariot, *Microcosmus squamiger* Michaelsen, 1927, *Bugula neritina* (Linnaeus, 1758), *Tricellaria inopinata* d’Hondt & Occhipinti Ambrogi, 1985 and *Zoobotryon verticillatum* (Delle Chiaje, 1822) were collected in marinas from Santa Maria (SMA) and of S. Miguel (SMI) islands (Azores). Dichloromethane and methanol extracts of these organisms were assessed against HeLa (cervix tumor), A549 (lung), MCF-7 (breast) and Vero (control) cell lines using the MTT method (Barreto, M.C., *et al.* in *Determination of Biological Activities. A Laboratory Manual*, 2012. ISBN 978-972-8612-82-5).

None of the extracts was active against A549 cell line or against any cell line in log phase up to 200 µg/mL. In lag phase, the dichloromethane extract from *Z. verticillatum* presented the best activity against MCF-7 cells and was the only extract active against HeLa cells (with selectivity Index of 1.94 and 4.64, respectively).The results obtained show the potential of *M*. *squamiger*, *B. neritina* and *Z. verticillatum* as sources of antitumor agents.

**Acknowledgments:** FRC/Azores ASMAS-M2.1.2/F/032/2011 and PREN.B/M3.1.5/I/116/2012/0000010. FCT/MEC for the financial support to the QOPNA research Unit (FCT UID/QUI/00062/2013), through national funds and where applicable co-financed by the FEDER, within the PT2020 Partnership Agreement.

### 4.6. Following the Removal of Fluoroquinolones on the Environment: An HPLC-FD Method (P7)

Carlos Afonso ^1,2,^*, Vanessa Ferreira ^1^, Catarina Amorim ^3^, Sara Cravo ^1^, Elizabeth Tiritan ^1,2,4^ and Paula Castro ^3^

^1^ Laboratório de Química Orgânica e Farmacêutica, Dep. Ciências Químicas, Faculdade de Farmácia da Universidade do Porto, Rua Jorge Viterbo Ferreira 228, 4050-313 Porto, Portugal

^2^ CIIMAR: Centro Interdisciplinar de Investigação Marinha e Ambiental da Universidade do Porto, Rua dos Bragas, 289, 4050-123 Porto, Portugal

^3^ CBQF—Centro de Biotecnologia e Química Fina—Laboratório Associado, Escola Superior de Biotecnologia, Universidade Católica Portuguesa/Porto, Rua Dt. António Bernardino Almeida, 4200-072 Porto, Portugal

^4^ CESPU, Instituto de Investigação e Formação Avançada em Ciências e Tecnologias da Saúde (IINFACTS), Rua Central de Gandra 1317, 4585-116 Gandra, Portugal

* Correspondence: cafonso@ff.up.pt

Antibiotic residues have been detected in various environmental matrices, such as surface and drinking waters. Although present at low levels (μg/L, ng/L), many antibiotics are bioaccumulative, pseudo-persistent and can promote resistance/alterations in bacterial populations (Doorslaer, V.X., *et al. Sci. Total Environ.* 2014, *500*, 250–269). In this work we present the biosorption of three widely used fluoroquinolones (FQ)—ofloxacin (OFL), norfloxacin (NOR) and ciprofloxacin (CPF)—to activated sludge (AS) and aerobic granular sludge (AGS). A HPLC-FD method was validated and optimized to follow the biosorption of the targeted FQ (Maia, A.S., *et al. J. Chrom. A* 2014, *1333*, 87–98). The validated method demonstrated good selectivity, linearity (r^2^ > 0.999), intra-day and inter-day precisions (RSD < 3%) and accuracy. LOD and LOQ were, respectively, 0.7 ng/mL and 1 ng/mL for the three FQ. Several parameters that can affect biossorption kinetics, namely, contact time, pH, and biosortion mass were also studied. At pH 7 AS showed better performance to biosorb OFL, NOR and CPF. The equilibrium data for AS showed a better fit to the Langmuir model, while AGS showed a better fit to the Freundlich model. The FQ could be desorbed from AGS at pH 3, 8 and 9, whereas at pH 4 the biosorption process was promoted.

### 4.7. The Antioxidant Activity of Novel Polyhydroxylated 3-Hydroxy-2-Styrylchromones and 3-Hydroxyflavones (P8)

Carina Proença ^1^, Marisa Freitas ^1^, Daniela Ribeiro ^1^, Joana L. C. Sousa ^2^, Artur M. S. Silva ^2^ and Eduarda Fernandes ^1,^*

^1^ UCBIO-REQUIMTE, Department of Chemical Sciences, Laboratory of Applied Chemistry, Faculty of Pharmacy, University of Porto, Rua de Jorge Viterbo Ferreira, 228, 4050-313 Porto, Portugal

^2^ Department of Chemistry & QOPNA, University of Aveiro, 3810-193 Aveiro, Portugal

* Correspondence: egracas@ff.up.pt

Flavonoids are chemically based on a fifteen-carbon skeleton consisting of two benzene rings (ring A and ring B) joined by a linear three carbon chain (C_6_-C_3_-C_6_) forming an oxygenated heterocycle pyran ring (ring C). 2-styrylchromones (2-SC), a small group of compounds characterized by the attachment of a styryl group to the 2-position of the chromone skeleton, have structural similarities with flavonoids, particularly those belonging to the class of flavones. Flavonoids possess many biological activities, from which the antioxidant properties are the best described. Considering the structural similarities of 2-SC and the fact that their styryl moiety may greatly contribute to their molecular stabilization under redox challenges, some of its biological activities are likely to be similar or even enhanced in comparison to flavonoids, although it needs to be experimentally confirmed. Thus, the purpose of the present study was to evaluate and compare the putative scavenging of reactive oxygen (ROS) and nitrogen (RNS) species by synthetic 3-hydroxyflavones and 3-hydroxy-2-styrylchromones, using *in vitro* non-cellular systems. The obtained results show that both groups of compounds have high capacity to scavenge ROS and RNS. Interestingly, the efficacy of 3-hydroxy-2-styrylchromones and 3-hydroxyflavones vary among the tested reactive species, constituting a good option as antioxidant agents.
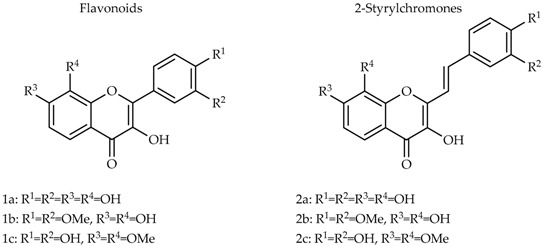


**Acknowledgments:** We would like to thank FCT (Fundação para a Ciência e Tecnologia, Portugal) for the financial support to UCIBIO-REQUIMTE through the project UID/Multi/04378/2013. M. Freitas and JLC Sousa acknowledge the FCT financial support for the Post-doctoral (SFRH/BPD/76909/2011) and Ph.D. (SFRH/BD/76407/2011) grants in the ambit of “POPH—QREN—Tipologia 4.1—Formação Avançada” co-sponsored by FSE and national funds of MCTES. We also would like to thank University of Aveiro and FCT/MEC for the financial support to the QOPNA research Unit (FCT UID/QUI/00062/2013), through national founds and where applicable co-financed by the FEDER, within the PT2020 Partnership Agreement.

### 4.8. An Example of the Key Role of HPLC in Medicinal Chemistry: Determination of Lipophilicity of a Xanthone Derivatives Library (P10)

Carla Fernandes ^1,2,^*, Álvaro Santos ^1^, José Soares ^1^, Saro Cravo ^1^, MariaElizabeth Tiritan ^1,2,3^, Carlos Afonso ^1,2^ and Madalena Pinto ^1,2^

^1^ Laboratório de Química Orgânica e Farmacêutica, Departamento de Ciências Químicas, Faculdade de Farmácia, Universidade do Porto, Rua Jorge Viterbo Ferreira 228, 4050-313 Porto, Portugal

^2^ Centro Interdisciplinar de Investigação Marinha e Ambiental (CIIMAR/CIMAR), Universidade do Porto, Rua dos Bragas 289, 4050-123 Porto, Portugal

^3^ CESPU, Instituto de Investigação e Formação Avançada em Ciências e Tecnologias da Saúde, Rua Central de Gandra, 1317, 4585-116 Gandra PRD, Portugal

* Correspondence: cfernandes@ff.up.pt

For the last several years, searching of new xanthone derivatives (XD) with potential pharmacological properties has remained in the area of interest of our group. In addition, the assessment of the drug-likeness of the bioactive XD synthesized is crucial in an early stage of the drug discovery pipeline considering that problems related to physicochemical properties are one of the main reasons of failure during pre-clinical trials.

Lipophilicity, commonly expressed by the logarithm of the partition coefficient (log P), is one of the most important physicochemical properties having a great impact both in pharmacokinetics (absorption, distribution, metabolism, excretion, and toxicity—ADMET) and pharmacodynamic processes.

In this work, we describe the lipophilicity of a series of XD, previously synthesized in our group (Fernandes, C., *et al.* *Bioorgan. Med. Chem.* 2014, *22*, 1049–1062), using two different methods: RP-HPLC (Ayouni, L., *et al.* *Chromatographia* 2005, *62*, 251–255) and vortex-assisted liquid-liquid microextraction coupled with liquid chromatography (VALLME-HPLC) (Roman, I.P. *et al.* *J Chromatogr.* *A* 2014, *1330*, 1–5). Both methods were validated accordingly with OCDE guidelines.

In the RP-HPLC method retention factors (log k) were determined and correlated to log P. The analyses were carried out under optimized isocratic conditions, using two different hydrophobic silica-based stationary phases (C8 and C18) and different water/methanol ratios as mobile phases. Linear correlations were found between log k values and the volume fraction of methanol in the mobile phase (R^2^ higher than 0.99) for both stationary phases. The log k values were extrapolated for pure aqueous eluent to correlate with the values obtained using VALLME method. Low sample consumption, low sensitivity to impurities, good accuracy and excellent reproducibility were observed.

The VALLME-HPLC method demonstrated to be a successful technique for log P evaluation. Linear correlations were obtained, with R^2^ ˃ 0.99 in all cases and, in most cases, low variation coefficients (%) were observed for all the XD tested.

HPLC technique was crucial for the determination of log P values, showing its importance in the evaluation of physicochemical parameters that can be useful for pharmacokinetics prevision.

**Acknowledgments:** This research was partially supported by the Strategic Funding UID/Multi/04423/2013 through national funds provided by FCT—Foundation for Science and Technology and European Regional Development Fund (ERDF), in the framework of the programme PT2020.

### 4.9. Investigation of the Interaction of Vancomycin with Synthetic Bacterial Muropeptides (P11)

Cátia Santos ^1^, Sérgio Filipe ^2^ and Maria Marques ^1,^*

^1^ LAQV@REQUIMTE, Departamento de Química, Faculdade de Ciências e Tecnologia, Universidade Nova de Lisboa, Campus de Caparica, 2829-516 Caparica, Portugal

^2^ Instituto de Tecnologia Química e Biológica, Universidade Nova de Lisboa, Av. Da República (EAN), 2780-157 Oeiras, Portugal

* Correspondence: mmbmarques@fct.unl.pt

In order to find new agents to combat bacteria resistance it is important to understand the details of the mechanism of action of glycopeptides antibiotics as vancomycin (Vorpagel, E., *et al.* *J. Am. Chem. Soc.* 2008, *130*, 13013–13022). Those antibiotics prevent the formation of peptidoglycan (PGN), the major component of the bacterial cell wall which is constituted by a glycan chain of alternating β(1 → 4)-linked *N*-acetylglucosamine (GlcNAc) and *N*-acetylmuramic acid (MurNAc), cross-linked by short peptide bridges (Höltje, J.V., *et al.* *Microbiol. Mol. Biol. Rev*. 1998, *62*, 181–203).

It is well established that the replacement of the last amino acid in the peptide chain linked to the MurNAc moiety changes the interaction with the glycopeptides antibiotics. However the interactions studies have been limited to the use of *N*-protected dipeptides and tripeptides (Kim, C., *et al.* *Bmb Rep.* 2008, *41*, 93–101; Jimenez-Barbero, J., *et al.* *Chem. Eur.* 2014, *20*, 7363–7372). In order to clarify how the different compositions of the bacterial fragments affect the recognition by vancomycin we have developed a study involving synthesis and screening of small cell wall bacterial fragments.

Herein we present our preliminary studies on the interaction between vancomycin and the synthesized compounds.

### 4.10. Identification and Characterization of Small Molecule Interactions with Transthyretin Amyloid Fibrils (P13)

Elisabete Ferreira ^1^, Pedro F. Cruz ^1^, Zaida L. Almeida ^1,2^, Marta S. Sousa ^2^ and Rui M. M. Brito ^1,2,^*

^1^ Coimbra Chemistry Centre, Chemistry Department, University of Coimbra, 3004-535 Coimbra, Portugal

^2^ Centre for Neuroscience and Cell Biology, University of Coimbra, 3004-517 Coimbra, Portugal

* Correspondence: brito@ci.uc.pt

Transthyretin (TTR) is an amyloidogenic protein involved in diseases characterized by progressive neuropathy and/or cardiomyopathy (Brito, R.M.M., *et al.* *Curr. Med. Chem. Immun. Endoc. Metab. Agents* 2003, *3*, 349–360). Currently, there is no fully effective treatment for TTR amyloidosis and several therapeutic strategies targeting different steps of the fibril formation pathway are under development (Ueda, M., *et al.* *Transl. Neurodegener.* 2014, *3*, 19–29). The disruption of amyloid aggregates and fibrils is one of those strategies. To properly select compounds for this purpose a specific screening protocol that can identify and characterize the interaction of the compounds with the fibrils must be developed. With this purpose in mind three wild type-TTR fibril formation protocols were studied by dynamic light scattering, circular dichroism, turbidimetry, transmission electron microscopy and fluorescence spectroscopy to select and characterize the most appropriate fibril formation protocol. The results show that the heat-induced fibril formation protocol at neutral pH has advantages over other fibril formation protocols but requires further characterization. Additionally, saturation transfer difference nuclear magnetic resonance was used to study the interaction of doxycycline with WT-TTR fibrils, and a DLS assay was developed to characterize the effect of this compound on fibril disruption.

### 4.11. Xanthone as a Valuable Scaffold to Identify New Promising Antitumor Agents via Inhibition of p53-MDM2 Interaction (P14)

Emília Sousa ^1,2,^*, Agostinho Lemos ^1^, Ana Sara Gomes ^1,3^, Andreia Palmeira ^1^, Lucília Saraiva ^3^ and Madalena Pinto ^1,2^

^1^ Laboratory of Organic and Medicinal Chemistry, Department of Chemical Sciences, Faculty of Pharmacy, University of Porto, R. Jorge Viterbo Ferreira, 228, 4050-313 Porto, Portugal

^2^ CIIMAR—Interdisciplinary Center of Marine and Environmental Research, Rua dos Bragas 289, 4050-123 Porto, Portugal

^3^ UCIBIO/REQUIMTE, Laboratory of Microbiology, Department of Biological Sciences, Faculty of Pharmacy, University of Porto, R. Jorge Viterbo Ferreira, 228, 4050-313 Porto,Portugal

* Correspondence: esousa@ff.up.pt

The activity of p53 tumor suppressor is regulated by MDM2 and MDMX, which are overexpressed in about half of human tumors. Disruption of p53-MDM2/MDMX interaction may represent an attractive strategy for cancer therapy. Xanthone derivatives have been reported as promising antitumor agents via inhibition of p53-MDM2 interaction, particularly gambogic acid (**1**), α-mangostin (**2**), and synthetic derivative pyranoxanthone LEM1 (**3**) (Leão, M., *et al. Biochem. Pharmacol.* 2013*, 85,* 1234–1245).

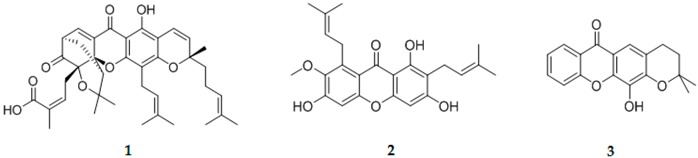


Based on the substitution pattern of xanthone LEM2, discovered as a potent antitumor agent, and on amine moiety of p53-MDM2 inhibitors, new potential disruptors with drug-like properties were obtained. By reductive amination, eleven LEM2-aminated derivatives were successfully synthesized. Their binding affinity was predicted by docking studies towards MDM2. Using yeast-based assays, their inhibitory activity on p53-MDM2 was investigated. These studies will provide the most favorable requirements for the construction of new antitumor agents.

**Acknowledgments:** Strategic Funding UID/Multi/04423/2013 through FCT and ERDF, REQUIMTE-Pest-C/EQB/LA0006/2013, PTDC/DTP-FTO/1981/2014.

### 4.12. Antimicrobial Activity and Biofilm Formation Inhibition of Alginate Microspheres of Dehydroabietic Acid (P18)

Íris Neto ^1,2^, Diogo Matias ^1^, Catarina Pinto Reis ^1^, Célia Faustino ^1,2,^* and Patrícia Rijo ^1,2,^*

^1^ CBIOS, Universidade Lusófona de Humanidades e Tecnologias, Campo Grande 376, 1749-024 Lisboa, Portugal

^2^ iMed.UL, Faculdade de Farmácia da Universidade de Lisboa, Av. Gama Pinto, 1649-003 Lisboa, Portugal

* Correspondence: patricia.rijo@ulusofona.pt (C.F.); cfaustino@ff.ulisboa.pt (P.R.)

A wide spectrum of biological activities including antitumor, anti-inflammatory and antimicrobial are displayed by the abietane dehydroabietic acid (DHA) (González, M.A., *et al. Eur. J. Med. Chem*. 2010, *45*, 811–816). Recently, DHA was shown to prevent *Staphylococcus aureus* bacterial colonization and also inhibiting biofilm formation (Fallarero, A., *et al.* *Int. J. Mol. Sci*. 2013, *14*, 12054–72). Considering these promising results, the aim of this study was to investigate the efficacy of DHA for inhibiting the biofilm formation as well as its efficiency against standard and isolate strains. Minimum Inhibitory Concentration (MIC) and Minimum Biofilm Inhibitory Concentration (MBIC) were determined by two-fold serial broth microdilution assay, as well as crystal violet staining methods (Stepanović, S., *et al.* *Apmis* 2007, *115*, 891–899). DHA showed MIC values between 15.63–500 µg/mL and a range of 32%–90% of biofilm inhibition, therefore demonstrating efficacy against resistant bacteria and their biofilms.

Future studies will focus on a synergistic effect between an antimicrobial synthetic molecule and an encapsulation polymer, namely a new abietane cationic amphiphile (ACA) derived from dehydroabietic acid.

### 4.13. Identification and Quantification of Blackberry Anthocyanin Metabolites in Human Plasma and Urine (P19)

Iva Fernandes ^1,^*, Ana Faria ^1,2,3^, Cláudia Marques ^2^, Victor de Freitas ^1^, Conceição Calhau ^2,4^ and Nuno Mateus ^1^

^1^ REQUIMTE, Departamento de Química e Bioquímica, Faculdade de Ciências, Universidade do Porto, Rua do Campo Alegre s/n, 4169-007 Porto, Portugal

^2^ Department of Biochemistry (U38-FCT), Faculty of Medicine, University of Porto, 4200-319 Porto, Portugal

^3^ Faculty of Nutrition and Food Sciences, University of Porto, 4200-465 Porto, Portugal

^4^ CINTESIS Center for Research in Health Technologies and Information Systems, Faculty of Medicine, Porto University, Rua Plácido da Costa, 4200-450 Porto, Portugal

* Correspondence: iva.fernandes@fc.up.pt

The aim of this work was to perform a human crossover trial in which volunteers ingested a blackberry fruit juice as an acute dose, containing mainly cyanidin-3-glucoside (Cy3glc) with or without 12% ethanol. A through screening analysis for anthocyanins and/or metabolites was performed in plasma and urine samples by mass spectrometry. Cy3glc in its parent form was detected in plasma and urine samples. The identification and quantification of anthocyanin conjugates was also performed yielding several sulphate, glucuronyl and methyl conjugates of Cy3glc and Cy.

Anthocyanins can be further metabolized by colon microbiota and excreted in the urine, yielding several phenolic acid and their metabolized forms. Some protocatechuic, vanilic, benzoic and hippuric acid conjugates were identified in urine samples, mainly with sulfate groups.

So far, it has been quite difficult to clearly assess both native and metabolized forms and also catabolites *in vivo* and to distinguish their different biological roles.

**Acknowledgments:** Financial support from Fundação para a Ciência e Tecnologia (FCT)—Fundo Social Europeu, Programa Operacional Potencial Humano da EU (POPH), PTDC/AGR-TEC/2227/2012, SFRH/BPD/75294/2010 and SFRH/BPD/86173/2012 is gratefully acknowledged.

### 4.14. Reactivity between Cork Extracts and Major Wine Components (P20)

Joana Azevedo ^1^, Paulo Lopes ^2^, Isabel Roseira ^2^, Miguel Cabral ^2^, Nuno Mateus ^1^ and Vítor Freitas ^1,^*

^1^ REQUIMTE/LAQV- Laboratório Associado Química Verde, Faculdade de Ciências da Universidade do Porto, Rua do Campo Alegre, 687, 4169-007 Porto, Portugal

^2^ Amorim e Irmãos S.A. Rua da Corticeira 66, 4535-173 Mozelos VFR, Portugal

* Correspondence: vfreitas@fc.up.pt

Different phenolics were found to migrate from different cork stoppers into bottled wine model solutions (Azevedo, J., *et al.* *Eur. Food Res. Technol.* 2014, *239*, 951–960), including phenolic acids aldehydes and trace amounts of complex polyphenols such as mongolicain A/B, castalagin/vescalagin, HHDP-galloyl-glucose were also present.

These compounds interfere in wine color and taste features (Glabasnia, A., *et al. J. Agric. Food Chem*. 2006, *54*, 3380–3390). Also, some compounds participate in polymerization reactions with some wine components, changing their sensorial properties (Quideau, S. *et al. Chem. Eur. J.* 2005, *11*, 6503–6513). In addition, it is acknowledged that phenolic acids and aldehydes can react resulting in complex structures found in aged wines (Pissarra, J., *et al.* *J. Food Sci*. 2003, *68*, 476–481).

Bearing this, the aim of this study is to evaluate the reactivity between cork extracts and major wine components and understand the impact of this reactivity in wine properties.

### 4.15. Synthesis of Dipeptides with β,β-Disubstituted Dehydroalanine and C^α,α^-Dimethylglycine Residues (P21)

Joana Lopes, Luís S. Monteiro * and Sílvia Pereira-Lima

Department of Chemistry, University of Minho, Gualtar, 4710-057 Braga, Portugal

* Correspondence: monteiro@quimica.uminho.pt

Non-proteinogenic amino acids are an important class of organic compounds, either because they have an intrinsic biological activity, or as part of peptides with antiviral, anti-tumor, anti-inflammatory or immunosuppressive activities (Fosgerau, K., *et al.* *Drug Discov. Today* 2015, *20*, 122–128). The incorporation of non-proteinogenic amino acids into peptides can change their structural, chemical and pharmacological properties (Albericio, F., *et al.* *Future* *Med. Chem.* 2012, *4*, 1527–1531).

Dipeptides containing *N*-(*tert*-butyloxycarbonyl)-*C^α,α^*-dimethylglycine as amine terminal residue and the methyl ester of β-hydroxyamino acids as carboxyl residue were prepared. These *N*-(*tert*-butyloxycarbonyl)-dipeptide derivatives were subject to dehydration using a *tert*-butylpyrocarbonate [(Boc)_2_O]/4-dimethylaminopyridine (Dmap) procedure (Ferreira, P.M.T., *et al.* *Eur. J. Org.* *Chem.* 2007, 5934–5949) to give *N*-(*tert*-butyloxycarbonyl)-dehydrodipeptide derivatives. These were treated with *N*-bromossuccinimide yielding a *N*-(*tert*-butyloxycarbonyl)-β,β-dibromodehydrodipeptide and *N*-(*tert*-butyloxycarbonyl)-β-bromo-β-substituted dehydrodipeptide derivatives. The brominated dehydrodipeptide derivatives were used in Suzuki-Miyaura cross-couplings reactions.

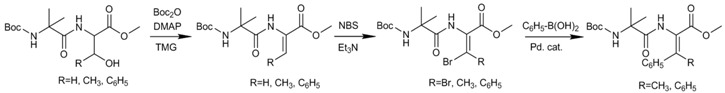


The synthesis of a dehydrodipeptide derivative containing β-brominated dehydroalanine as amine terminal residue and *C^α,α^*-dimethylglycine as carboxyl residue was also carried out.

### 4.16. Synthesis of 3-Oxo-β-Sultam Activity-based Probes for Biomarker Discovery in Chronic Obstructive Pulmonary Disease (P23)

Luís Carvalho * Susana Lucas and Rui Moreira

Research Institute for Medicines (iMed.ULisboa), Faculty of Pharmacy, Universidade de Lisboa, Av. Prof. Gama Pinto, 1649-003 Lisboa, Portugal

* Correspondence: luis.carvalho@ff.ulisboa.pt

Chronic Obstructive Pulmonary Disease (COPD) refers to a condition of inflammation with progressive weakening of the structure of the lung and irreversible narrowing of the airways (Lucas, S.D. *et al. Med. Res. Rev*. 2013, *33* (Suppl. S1), e73–e101). Novel biomarkers for this disease are urgently needed. COPD induced inflammation modulates proteolytic enzymes in the lungs, like Human Neutrophil Elastase (HNE), which are clinically relevant for the diagnosis of COPD. Adequately targeted probes can be designed to quantify the active catalytic state of these enzymes (Carvalho, L.A.R., *et al.* *MedChemComm* 2015, *6*, 536). Following the extensive work of our group with HNE inhibitor synthesis (Mulchande, J., *et al. J. Med. Chem.* 2008, *51*, 1783) we developed a library of HNE inhibitors and activity-based probes (ABPs) based on the 3-oxo-β-sultam scaffold (Tsang, W.Y., *et al. Org. Biomol. Chem*. 2007, *5*, 3993).

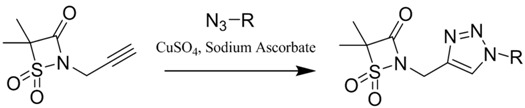


These ABPs will be used to covalently tag the active enzyme, providing protein activity quantification by proteomics-based analysis. We envisage the study and validation of HNE as a potential biomarker in COPD.

**Acknowledgments:** Fundação para a Ciência e a Tecnologia (Portugal) (PEst-OE/SAU/UI4013/2014).

### 4.17. Chemical Acylation vs. Enzymatic Esterification of Malvidin-3-Glucoside-Rich Wine Extract with Oleic Acid (P24)

Luis Cruz *, Marta Guimarães, Victor de Freitas and Nuno Mateus

REQUIMTE/LAQV, Departamento de Química e Bioquímica, Faculdade de Ciências, Universidade do Porto, Rua do Campo Alegre, 687, 4169-007 Porto, Portugal

* Correspondence: luis.cruz@fc.up.pt

The structural modification of anthocyanins (water soluble pigments) into more lipophilic compounds is very important to expand their application in the food, medicinal and cosmetic industries (Cruz, L., *et al.* *Food Chem.* 2015, *174*, 480–486; Ardhaoui, M., *et al.* *Biocatal. Biotransform.* 2004, *22*, 253–259). In this work, the synthesis of anthocyanin oleic acid ester derivatives was achieved by chemical and enzymatic approaches.

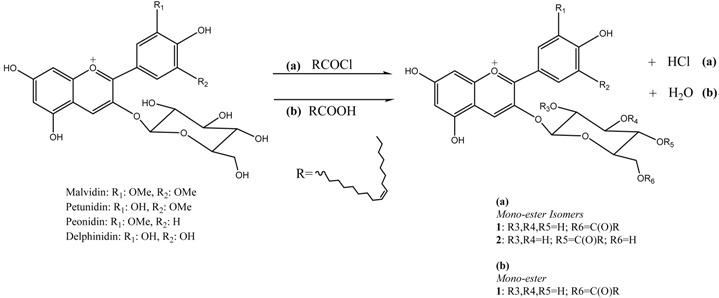


Enzymatic approach allowed to synthesize mv3glc-oleic acid ester which was structurally characterized by mass spectrometry and for the first time by NMR spectroscopy. Enzymatic reaction revealed to be more efficient and regioselective in the conversion of native anthocyanins into their ester product rather than the chemical reaction. Antioxidant features of the obtained products by means of DPPH and FRAP assays confirms that their antioxidant potential was not compromised, which is an important insight for future technological applications.

### 4.18. Synthesis of Innovative Anticoagulant Hybrids (P26)

Marta Correia-da-Silva ^1,2,^*, Catarina Carvalho ^1^, Emília Sousa ^1,2^ and Madalena Pinto ^1,2^

^1^ Laboratório de Química Orgânica e Farmacêutica, Departamento de Ciências Químicas, Faculdade de Farmácia da Universidade do Porto, Rua Jorge Viterbo Ferreira 228, 4050-313 Porto, Portugal

^2^ Centro Interdisciplinar de Investigação Marinha e Ambiental, Universidade do Porto, Rua dos Bragas 289, 4050-123 Porto, Portugal

* Correspondence: m_correiadasilva@ff.up.pt

In this work, a hybridization strategy was planned in order to develop new anticoagulants joining a coumarin scaffold with a heparin-like sugar sulfated moiety. With this approach we expected to mimetize the sulfated polysaccharide anticoagulants, while adding some hydrophobic character to the resulting molecule. Due to the evidence that 6-substituted coumarins with heteroaromatic rings can act as FXa inhibitors (Amin, K.M., *et al.* *Bioorg. Chem.* 2014, *52*, 31–43; Frédérick, R., *et al.* *J. Med. Chem.* 2005, *48*, 7592–7603), the 6-hydroxycoumarin (**1**) was selected for molecular modification and triazole was used as a linker between the coumarin scaffold and the glucosidic moiety (compounds **5**–**7**). For structure-activity relationships purposes, the syntheses of 7-triazole-linked coumarin glucosides (compounds **13**, **14**, and **15**) as well as of sulfates of non-triazole linked 6- and 7- coumarin glucosides (compounds **10**, **18**, and **19**) were performed.

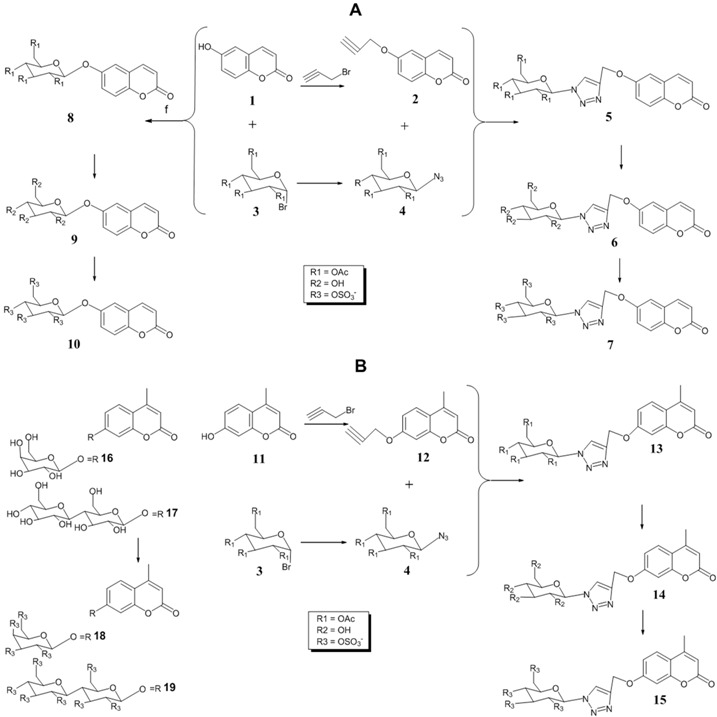


In conclusion, thirteen new coumarin derivatives were obtained, *i.e.*, two propynyl, three acetyl glucosides, three glucosides, and five sulfated derivatives, and the structure elucidation of the synthesized compounds was stablished by IR, NMR, and HRMS for the first time. This small library of compounds will allow the study of the effect of the presence of the triazole moiety on the anticoagulant activity and mode of action of these new anticoagulant hybrids.

### 4.19. Identification of Complexes Formed Between Salivary Proteins and Procyanidin B3 by LC-MS. Effect of Concentration and Saliva Profile (P27)

Maria Rosa Perez Gregorio *, Ricardo Dias, Nuno Mateus and Victor de Freitas

LAQV/REQUIMTE, Departamento de Química e Bioquímica, Faculdade de Ciências da Universidade do Porto, Rua do Campo Alegre 687, 4169-007 Porto, Portugal

* Correspondence: maria.gregorio@fc.up.pt

Astringency is an important factor in food quality. Different theories about astringency mechanism have been attributed but the most established process is the interaction between food tannins and human salivary proteins (Kallithraka, S., *et al. J. Sens. Stud.* 1998, *13*, 29–43). Several factors could influence the tannin-protein interaction such as the human salivary protein profile, the tannin tested and the tannin/protein ratio (Soares, S., *et al. Food Res. Int.* 2012, *49*, 807–813). Highlight the astringency mechanism through the study of tannin-protein interactions became relevant. The goal of this study aims to study the effect of different salivas (A, B and C) and different tannin concentration (0.5 and 1 mg/mL) in the interaction process. Human salivary proteins are divided into different groups mainly histatins (His), statherins (Stah), proline rich proteins (PRPs) and cystatins (Huq, N.L., *et al. Int. J. Pep. Res. Ther.* 2007, *13*, 547–564). This study is is focused in the identification of new procyanidin B3-salivary protein complexes complexes created between a common food tannin, the procyanidin B3 (B3), and the Stath, His and PRPs originating from saliva with different protein profiles.

### 4.20. Determination and Comparison of the Chemical Composition of Calendula L. species Growing in Continental Portugal (P28)

Maria Faustino ^1,^*, Ana M. L. Seca ^3,4^, Paulo Silveira ^1,2^ and Diana C. G. A. Pinto ^3^

^1^ Department of Biology, University of Aveiro, 3810-193 Aveiro, Portugal

^2^ CESAM, University of Aveiro, 3810-193 Aveiro, Portugal

^3^ Department of Chemistry & QOPNA, University of Aveiro, 3810-193 Aveiro, Portugal

^4^ DCTD, University of Azores, Rua Mãe de Deus, 9501-801 Ponta Delgada, Portugal

* Correspondence: maria.vf9@ua.pt

The flora of Continental Portugal includes three species of *Calendula* L. (*Calendula officinalis* L., *C. arvensis* L., *C. suffruticosa* Vahl), one of which comprises three subspecies (*C. suffruticosa* subsp. *algarbiensis* (Boiss.) Nyman, *C. suffruticosa* subsp. *lusitanica* (Boiss.) Ohle and *C. suffruticosa* subsp. *cinerea* (Ohle) P.Silveira & A.C.Gonç.). *C. officinalis* is recognised for its medical properties and its chemical composition has been widely studied (Muley, B. P., *et al. J. Pharm. Res.* 2009, *8*, 455; Safdar, W., *et al. Int. J. Cell Mol. Biol.* 2010, *1*, 108). Nevertheless, little is known about the chemical composition of *C. arvensis* and even less regarding the different subspecies of *C. suffruticosa*. Therefore, the present study aims the elucidation of these plants’ chemical composition and to compare and identify differences and/or similarities among them.

To accomplish this, one sample of each *taxon*, *C arvensis*, *C. officinalis*, and *C suffruticosa* subsp. *lusitanica*, and two samples, from two different populations, of *C. suffruticosa* subsp. *algarbiensis* were collected in the field, washed with running water, and dried in a woven at 40 °C until stabilization of weight was reached. The hexane extract of each *taxon* was obtained from dried and powdered plant and completely characterized by GC-MS after silylation, which allowed the identification and quantification of their constituents.

The achieved data showed the presence of mono- and disaccharides, terpenoids, fatty acids, sterols, alkanes, long chain alcohols and some amino acids. It was found that the monosaccharides and fatty acids are the most abundant families in *C. officinalis* being the palmitic acid and α-linoleic acid the most abundant compounds. The last one was also found in higher quantities in *C. arvensis*. Fatty acids like α-linoleic acid, palmitic acid and linoleic acid are also the most abundant in *C. suffruticosa* subsp. *lusitanica*. Lastly, the two samples of *C. suffruticosa* subsp. *algarbiensis* showed in major quantities a branched alkane and one compound from the ursano family. Some carbohydrates as well as lignoceric acid and linoleic acid were described for the first time in the *Calendula* L. genus.

Through the accomplished findings, including a preliminary Principal Component Analysis (PCA), a taxonomic differentiation among the *taxa* can be made. Irrelevant variations were also found in the two samples of the subsp. *algarbiensis*. The compounds detected for the first time improved our knowledge of the chemical profile of this genus. Additionally, some of the reported compounds have a major importance on a nutritional level.

**Acknowledgments:** We thank the Instituto da Conservação da Natureza e das Florestas for allowing the collection of the samples of *C. suffruticosa* subsp. *lusitanica*. We would like to thank University of Aveiro and FCT/MEC for the financial support to the QOPNA Research Unit (FCT UID/QUI/00062/2013), and to the CESAM RU (UID/AMB/50017), through national founds and where applicable co-financed by the FEDER, within the PT2020 Partnership Agreement.

### 4.21. Development of Antitumoral Monastrol Analogues: Synthesis, Cytotoxicity Evaluation and SAR Studies (P30)

Mariana Matias ^1,2,^*, Samuel Silvestre ^1,2^, Amílcar Falcão ^2,3^ and Gilberto Alves ^1,2^

^1^ CICS-UBI—Health Sciences Research Centre, University of Beira Interior, Av. Infante D. Henrique, 6200-506 Covilhã, Portugal

^2^ CNC—Center for Neuroscience and Cell Biology, University of Coimbra, 3004-517 Coimbra, Portugal

^3^ Pharmacology Department, Faculty of Pharmacy, University of Coimbra, Pólo das Ciências da Saúde, Azinhaga de Santa Comba, 3000-548 Coimbra, Portugal

* Correspondence: mariana.r.matias@gmail.com

Cancer persists as one of the major global public health concerns due to its large incidence and mortality. Searching for novel anticancer agents with higher potency and lower toxicity, our work aims the development of monastrol analogues. For this, forty five dihydropyrimidin(thi)ones were synthesized *via* the Biginelli reaction by condensation of an aldehyde, a β-ketoester and urea/thiourea. The screening of the antiproliferative effects of the compounds was evaluated at 30 µM on MCF-7, T47D, LNCaP, HepaRG, Caco-2 and NHDF cell lines by the MTT assay. The concentration inducing 50% inhibition of cell growth (IC_50_) was assessed for the most toxic compounds using different concentrations (0.01, 0.1, 1, 10, 50 and 100 µM). The results revealed that the compounds did not show significant toxicity neither in normal dermal cells (NHDF) nor in prostatic and breast (T47D) cancer cell lines; however, the chloro-containing compounds of the urea series showed selective toxicity for HepaRG cells (5.28 µM ≤ IC_50_ ≤ 15.9 µM) whereas their thiourea analogs evidenced lower selectivity, being significantly toxic for hepatic, colon and breast (MCF-7) cancer cell lines (0.749 µM ≤ IC_50_ ≤ 31.9 µM for HepaRG; 5.51 µM ≤ IC_50_ ≤ 13.7 µM for Caco-2; and 2.95 µM ≤ IC_50_ ≤ 10.9 µM for MCF-7). Thus, it was found that the molecules containing chloro atoms in their structure, particularly those belonging to urea series, demonstrated selective toxicity for hepatic cancer cells. Additional studies are ongoing to understand what mechanisms of action are involved in the toxicity of these molecules as well as the existence of differences between the two series.

**Acknowledgments:** We thank the Fundação para a Ciência e Tecnologia (SFHR/BD/85279/2012) for financial support as well as POPH-QREN which is co-funded by FSE and MEC.

### 4.22. d-Penicillamine- and L-Cysteine-Derived Thiazolidine Catalysts: An Efficient Approach to Both Enantiomers of Secondary Alcohols (P32)

M. Elisa Silva Serra *, Dora Costa, Dina Murtinho and Teresa M. V. D. Pinho e Melo

Centro de Química and Department of Chemistry, University of Coimbra, 3004-535 Coimbra, Portugal

* Correspondence: melisa@ci.uc.pt

The enantioselective alkylation of aromatic aldehydes with diethylzinc in the presence of chiral ligands is a valuable method for synthesizing chiral secondary alcohols. Chiral 1,3-thiazolidine-4-carboxylates derived from l-cysteine have been sparingly used as catalysts in this reaction, giving secondary alcohols with good to excellent enantiomeric excesses (Meng, Q., *et al.* *Tetrahedron Asymmetry* 2000, *11*, 4255–4261; Jin, M.J., *et al.* *Bull. Korean Chem. Soc.* 2002, *23*, 509–510). To the best of our knowledge, structurally identical D-penicillamine-derived thiazolidines **2** have not been used in these reactions. These two types of thiazolidines, easily obtained by a simple synthetic process could bring singular advantages to many catalytic processes. Because l-cysteine has an (*S*) chiral center, while D-penicillamine’s chiral center is (*R*), the alkylation of several aromatic aldehydes which we carried out using **1** and **2** as catalysts, gave (*S*) and (*R*) alcohols, respectively, leading to a myriad of useful chiral products with opposite absolute configurations in excellent yields and ee up to >99%.

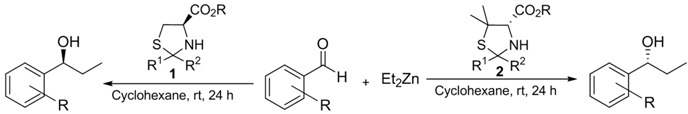


### 4.23. Structure-Based Pharmacophore Modeling and Virtual Screening of Metallo-Beta-Lactamase Inhibitors (P34)

Miguel Ângelo Costa ^1,^*, Gabriela Jorge da Silva ^1,2^, Maria Luisa Sá e Melo ^1,2^ and Maria Manuel C. Silva ^1,2^

^1^ Faculdade de Farmácia, Universidade de Coimbra, Pólo das Ciências da Saúde, 3000-548 Coimbra, Portugal

^2^ Center for Neuroscience and Cell Biology, University of Coimbra, Largo Marquês de Pombal, 3004-517 Coimbra, Portugal

* Correspondence: miguelangelocosta@gmail.com

Hydrolysis by β-lactamases (BL) is one of the major mechanisms that drive resistance to β-lactam antibiotics. The major strategy to overcome their activity is the use of inhibitors that have little to none antibiotic activity, but bind with greater affinity to BLs, allowing an effective antibiotic therapy.

The metallo-BL (MBL), are a structurally distinct BL class that hydrolyze almost all classes of β-lactams and are not inhibited by any marketed inhibitor. They are increasingly produced by clinical bacteria and their prevalence will keep growing as some types of MBL are encoded in mobile genetic elements (Arakawa, Y., *et al.* *Antimicrob. Agents Chemother.* 1995, *39*, 1612–1615; Da Silva, G.J., *et al. FEMS Microbiol. Lett.* 2002, *215*, 33–39). It is thus, evident, the need for solutions that allow β-lactam antibiotics to keep their effectiveness (Da Silva, G. J., *et al. FEMS Microbiol. Lett.* 2002, 215, 33–39; Klingler, F.M., *et al. J. Med. Chem.* 2015, *58*, 3626–3630).

The study of inhibitors for MBLs has highlighted some classes of compounds potentially useful for the design of a successful inhibitor in the future. However, none have yet entered further development stages (Klingler, F.M., *et al. J. Med. Chem.* 2015, *58*, 3626–3630; Fast, W., *et al.* *Biochim. Biophys. Acta* 2013, *1834*, 1648–1659).

In this study, we have developed a structure-based pharmacophore model to screen large compound databases (NCI, ZINC and DrugBank) for candidate ligands to IMP-1 MBL, followed by docking of the successful results.

### 4.24. Synthesis and Structure−Activity Relationships of Novel Pyrroloquinolone-based analogues as Potent Antimalarials (P35)

Moni Sharma ^1^, Miguel Prudêncio ^2^, Paul O’Neill ^3^ and Rui Moreira ^1^

^1^ Research Institute for Medicines and Pharmaceutical Sciences (iMed.UL), Faculty of Pharmacy, University of Lisbon, Av. Prof. Gama Pinto, 1649-019 Lisbon, Portugal

^2^ Instituto de Medicina Molecular, Faculty of Medicine, University of Lisbon, Av. Egas Moniz, 1649-028 Lisbon, Portugal

^3^ Department of Chemistry, University of Liverpool, Liverpool, L69 3BX, UK

* Correspondence: moni@ff.ul.pt

The most virulent human malaria parasite, *Plasmodium falciparum*, is responsible for 198 million cases of malaria worldwide and its burden is heaviest in the Sub-Sharan region (Factsheet on the World Malaria Report 2014). The emergence of drug-resistant pathogens is a major threat to human health, and *P. falciparum* exhibits high propensity to develop resistance to drug that has been deployed against it on a large scale (Pelleaua, S., *et al.* *Proc. Natl. Acad. Sci*. *USA* 2015, *112,* 11672; Muregi, F.W., *et al.* *Curr. Pharm. Des*. 2012, *18*, 3505). Therefore, there is an urgent need to identify new antimalarial agents to combat emerging resistant strains with a new mode of action. Pyrroloquinolones are an extremely important class of heterocycles with wide applications in the areas of medicinal chemistry (Howard, B.L., *et al.* *ACS Chem. Biol*. 2015, *10*, 1145; Lanter, J.C., *et al.* *J. Med. Chem.* 2004, *47*, 656–662; Sui, Z., *et al.* *J. Med. Chem*. 2002, *45*, 4094–4096; Ukita, T., *et al.* *J. Med. Chem.* 2001, *44*, 2204–2218). Pyrroloquinolones are potent and selective inhibitor of 3′,5′-cyclic nucleotide phosphodiesterases (PDEs). PDEs have been well studied as potential targets in various eukaryotic organisms. Recently, Deprez *et al.*, reported the Plasmodium phosphodiesterase activity of tadalafil (Beghyn, T.B., *et al.* *J. Med. Chem*. 2012, *55*, 1274−1286). On the otherside, The Liver stage of Plasmodium obligatorily precedes erythrocytic stages and therefore offers a potential drug target Rodrigues (Prudêncio, T., *et al.* *J. Med. Chem.* 2012, *55*, 995–1012).

On the basis of these, we have designed and synthesized a series of highly substituted pyrroloquinolones, which has shown a potent activity against both the erythrocytic and exoerythrocytic forms of Plasmodium parasites. Our activity results represent a new structural lead for further optimization as dual-stage antimalarials.
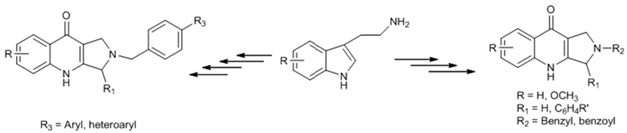


### 4.25. Enrichment of Glycidamide Adducts of Human Serum Albumin (P36)

João P. Nunes, Catarina Charneira, Inês L. Martins, Cristina C. Jacob, M. Matilde Marques and Alexandra M.M. Antunes *

CQE-IST, Centro de Química Estrutural, Instituto Superior Técnico, Universidade de Lisboa, 1049-001 Lisboa, Portugal

* Correspondence: alexandra.antunes@tecnico.ulisboa.pt

Glycidamide (GA) is considered the toxic metabolite of the food carcinogen acrylamide, as it interacts covalently with DNA bases (Gamboa da Costa, G., *et al.* *Chem. Res. Toxicol*. 2003, *16*, 1328–1337) and induces a tumor spectrum similar to acrylamide in rodent bioassays (Beland, F.A., *et al.* *Food Chem. Toxicol*. 2015, *86*, 104–115). Besides DNA bases, GA also binds with hemoglobin to form protein adducts, which are useful biological markers of acrylamide exposure (Tareke, E., *et al.* *Toxicol. Appl. Pharmacol.* 2006, *217*, 63–75). However, these adducts are usually detected by gas chromatography coupled with mass spectrometry (GC-MS) following a derivatization procedure, which raises some questions about the automation of the methodology due to the complexity of sample preparation. Methodologies based on liquid chromatography—mass spectrometry analysis of protein digests provide better alternatives, involving conditions that result in low thermal input and mild ionization. Moreover, these methodologies offer a wider applicability, enabling the analysis of covalent adducts formed with polar electrophiles. Nonetheless, levels of protein adducts are small compared to the unmodified proteins, and therefore they are difficult to detect and identify without prior enrichment. To increase analytical sensitivity for detecting less-abundant protein adducts, we report herein a methodology to enrich GA adducts of Human Serum Albumin (HSA) prior to nano-electrospray ionization mass spectrometry analysis. Following trypsin digestion of HSA modified *in vitro* with GA, the GA-modified peptides were enriched through solid-phase extraction and then analyzed by nano liquid chromatography coupled to quadrupole-time-of-flight mass spectrometry (Q-ToF) using a data-dependent auto-MS/MS method. This analytical method provides a straightforward approach for detecting GA-protein adducts and, therefore, stimulating the common use of HSA adducts as biomarkers of acrylamide exposure.

**Acknowledgments:** This work was supported in part by Fundação para a Ciência e a Tecnologia (FCT), Portugal (RECI/QEQ-MED/0330/2012, UID/QUI/00100/2013 and IF/01091/2013/CP1163/CT0001). ILM and CC thank FCT for doctoral fellowships (SFRH/BD/75426/2010 and SFRH/BD/102846/2014, respectively). AMM also acknowledges Programa Operacional Potencial Humano from FCT and the European Social Fund (IF/01091/2013), and the LRI Innovative Science Award. We thank the Portuguese MS network (IST node) for providing access to the facility.

### 4.26. Application of Molecular Dynamics and Alchemical Free Energy Calculations to Study the Interaction of β-2 Microglobulin with Thioflavin-T (P40)

Pedro M. P. Fernandes ^1,^*, Bruno L. Victor ^1,2^, Cândida G. Silva ^1,3^ and Rui M.M. Brito ^1,3^

^1^ Coimbra Chemistry Centre, Department of Chemistry, University of Coimbra, 3004-535 Coimbra, Portugal

^2^ BSIM^2^—Biomolecular Simulations, Biocant Park, 3060-197 Cantanhede, Portugal

^3^ Center for Neuroscience and Cell Biology, University of Coimbra, 3004-504 Coimbra, Portugal

* Correspondence: pmpf@student.uc.pt; brito@ci.uc.pt

The development of new and better molecular probes for the early diagnosis of amyloid diseases such as Alzheimer’s, Parkinson’s and type II diabetes are of critical essence (Buell, A.K., *et al, Phys. Chem. Chem. Phys.* 2011, *13*, 20044–20052). We employed molecular dynamics (MD) simulations to characterize the interaction of the protein β-2 microglobulin (β2m) with the well-known amyloid probe thioflavin-T (ThT). The results obtained are in agreement with reports found in the literature, where residues Q8 and Y10 of β2m were shown to be crucial for the interaction with ThT (Wolfe, L.S., *et al.* *Proc. Natl. Acad. Sci*. *USA* 2010, *107*, 16863–16868). To better characterize the topological constraints imposed by these residues, we have constructed *in silico* the mutants Q8A, Y10A and Y10F, and performed several MD simulations. The results show that the aromatic ring of Y10 is crucial for the interaction of ThT with β2m. Additionally, the energetic contribution of these residues to the stability of the β2m-ThT complex was quantified using alchemical free energy calculations (AFEC) which also confirmed the importance of this interaction.

### 4.27. Activation of Cytotoxic Responses of NK Cells (P41)

Pedro F. Pinheiro *, Gonçalo C. Justino and M. Matilde Marques

Centro de Química Estrutural, Instituto Superior Técnico, Universidade de Lisboa, 1049-001 Lisboa, Portugal

* Correspondence: pedro.pinheiro@tecnico.ulisboa.pt

Natural killer (NK) cells are a type of cytotoxic lymphocyte critical to the innate immune system. NK cells provide rapid responses to viral-infected cells, and play a role in tumor immunosurveillance by directly inducing the death of tumor cells. Instead of acting via antigen-specific receptors, lysis of tumor cells by NK cells is mediated by alternative receptors, including NKG2D, NKp44, NKp46 and NKp30 (Terunuma, H., *et al. Int. Rev. Immunol*. 2008, *27*, 93–110).

In recent developments, B7-H6, a surface protein present on a broad panel of tumor cells including lymphoma, melanoma, and carcinoma, was identified as a ligand for the NKp30 receptor. The structure of the NKp30-B7H6 complex has also been resolved (Li, Y., *et al. J. Exp. Med*. 2011, *208*, 703–714). The comparison between the 3D structures of unbound and B7-H6-bound NKp30 demonstrated marked conformational changes that may be a key-factor for the NK-response activation role of B7-H6.

Our current work aims at designing a family of small organic molecules (SOMs) capable of mimicking the effect of B7-H6 on the NKp30 receptor. A combination of computational docking and molecular dynamics tools was extensively used to scan several ligand libraries, yielding core-structures as possible ligands for the receptor. These were further optimized to generate lead structures that will be screened as NKp30 ligands through mass spectrometry tools.

The main goal is to obtain an SOM capable of inducing the activation of an NK response, through binding of the NKp30 receptor, and structurally amenable to derivatization with specific tumor-targeting molecular units to produce a specific immune response against cancer cells.

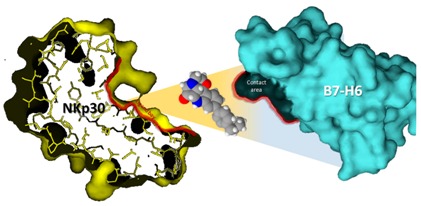


**Acknowledgments:** This work has been carried out with financial support from FCT-funded projects (RECI/QEQ-MED/0330/2012, RECI/QEQ-QIN/0189/2012 and UID/QUI/00100/2013) and grants (SFRH/BD/110945/2015 to PFP and SFRH/BPD/108258/2015 to GCJ) and from Liga Portuguesa Contra o Cancro (LPCC/NRS-Terry Fox grant 2015).

### 4.28. Production of Surface Carboxymethylated Cellulose Filters in Aqueous Medium (P46)

Ricardo Chagas ^1,^*, Sara Monteiro ^2^, Ricardo B. Ferreira ^2^ and Luísa M. Ferreira ^1^

^1^ LAQV, REQUIMTE, Departamento de Química, Faculdade de Ciências e Tecnologia, Universidade Nova de Lisboa, Campus da Caparica, Quinta da Torre, 2829 -516 Caparica, Portugal

^2^ LEAF, Instituto Superior de Agronomia, Universidade de Lisboa, Portugal

* Correspondence: r.chagas@campus.fct.unl.pt

Cellulose is considered one of the most promising polymeric resource as alternative for petroleum-based polymers (Pang, J., *et al.* *Materials* 2013, *6*, 1270–1284.). It has many advantages such as low cost, biocompatibility and biodegradability (Fink, D.K., *et al.* *Angew. Chem. Int*. 2005, *44*, 3358–3393). Carboxymethyl cellulose (CMC) is the most important ionic cellulose ether widely used due to its thickening, suspending and film forming properties (Ramos, L. A., *et al.* *Carbohydr. Polym*. 2005, *60*, 259–267). With the aim of producing cellulose filters able to adsorb positive proteins at low pH, we have produced surface carboxymethylated cellulose filters using commercial filters has starting material.

Commercial filters were carboxymethylated using sodium hydroxide (NaOH) and monochloroacetic acid (MCA) in aqueous medium under heterogeneous conditions. The produced CMCs were characterized by FTIR and protein adsorption capacity was evaluated by static method using lysozyme. The CMC surface modified filters had a DS between 0.139 and 0.179 and an adsorption capacity between 2.5 to 10.4 mg of lysozyme per gram of cellulose at pH 5. Hence, it is possible to produce water insoluble carboxymethylated cellulose filters for the adsorption of positive proteins at low pH in aqueous medium.

### 4.29. Synthesis and in Vitro Activity in HCT116 Human Colon Cancer Cells of New Phenol-Pyrimido[5,4-d]pyrimidine Conjugates (P47)

Ricardo Nunes ^1^, Fernanda Proença ^1^, Cristina Pereira-Wilson ^2^ and Maria Alice Carvalho ^1^

^1^ CQUM—Centre of Chemistry, Department of Chemistry, University of Minho, Campus de Gualtar, 4710-057 Braga, Portugal

^2^ CITAB—Centre for the Research and Technology of Agro-Environment and Biological Sciences, Department of Biology, University of Minho, Campus de Gualtar, 4710-057 Braga, Portugal

* Correspondence: ricardo_nunes000@hotmail.com

Colon cancer is the third cause of death worldwide and it is more frequent in industrialized countries. In the USA it is the second cause of death and in Europe approximately 25,000 cases are reported every year. The existent treatments are based in the combination of 5-Fluorouracil (5-FU) and LV or 5-FU alone. However, these treatments are not effective in all patients due to multidrug resistance developed by tumour cells and the lack of selectivity (Levin, B., *et al. J.* *Gastroenterology* 2008, *134*, 1570–1595; Labianca, R*., et al. J. Crit. Rev. Oncol. Hematol.* 2010, *74*, 106–133).

The pyrimido[5,4-*d*]pyrimidine ring system **1** has been attracted enormous attention of the scientific community mainly due to its activity as anti-tumour (Sanghvi, Y.S., *et al.* *J. Med. Chem.* 1989, *32*, 629–637), antiviral (Tenser, R.B., *et al.* *Antimicrob. Agents Chemother.* 2001, *45*, 3657–3659., antioxidant (De La Cruz, J.P., *et al.* *Lipids* 1992, *194*, 192–194; Vartale, V.P., *et al.* *Int. J. Res. Pharm. Chem.* 2015, *5*, 208–214), antifungal (Sharma, P., *et al.* *Bioorg. Med. Chem. Lett.* 2004, *14*, 4185–4190) and hepatoprotective (El-Moghazy, S.M., *et al.* *Sci. Pharm.* 2011, *79*, 429–447) properties. In our research group, pyrimido[5,4-*d*]pyrimidines were identified as highly active against colon cancer cells (HCT116 p53-wt), with IC_50_ lower than that of 5-FU.
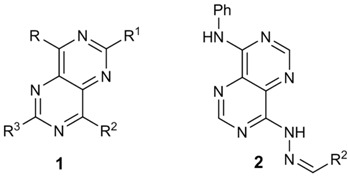


Recently, we synthesized new derivatives of pyrimido[5,4-*d*]pyrimidines, **2**, combining pyrimido[5,4-*d*]pyrimidine core with phenolic aldehydes. The *in vitro* activity of the new compounds was assessed in HCT116 human cancer cells. The new compounds showed high activity that depends on the R^2^ group. The synthetic approach and the biological results will be presented.

### 4.30. Synthesis and Biological Evaluation of Bile Acid Polyamine Amides (P50)

Sandrina Maçãs ^1,^*, Elisabete Alves ^1^, João Martins ^1^, Adriana Santos ^1^, Carla Cruz ^1^ and Samuel Silvestre ^2^

^1^ CICS-UBI—Health Sciences Research Centre, University of Beira Interior, R. Marquês de Ávila e Bolama, 6201-001 Covilhã, Portugal

^2^ Centre for Neuroscience and Cell Biology, University of Coimbra, 3004-517 Coimbra, Portugal

* Correspondence: sandyna7@gmail.com

Bile acids have been widely applied in synthetic chemistry mainly due to their enantiomeric purity, high stability of the steroid nucleus, reactivity of the side chain groups, low cost and ready availability, as well as anticancer properties (Davis, A., *Molecules* 2007, *12*, 2106–2122). This work aims to develop potential new antitumor agents by combining the bile acid core with different polyamines.

The synthesis started with the acylation of lithocholic or deoxycholic acid with succinic or phthalic anhydride, followed by the coupling with different polyamines. Then their antiproliferative effects were evaluated by the 3-(4,5-dimethylthiazol-2-yl)-2,5-diphenyltetrazolium bromide (MTT) assay in LNCaP, MCF-7, T47D, U87 and NHDF cell lines and by flow cytometry (MCF-7 cells).

Of these, the compound **1** evidenced a relevant cytotoxic effect on hormone-dependent cells, and flow cytometry studies suggest that it can promote cytotoxicity with 13% of cells death at 50 µM. The compound **2** showed an antiproliferative effect only on LNCaP cells.

In conclusion, it was possible to synthetize novel aminated steroids some of which show promising biological activity and can constitute interesting hit compounds for the development of potential new anticancer agents.

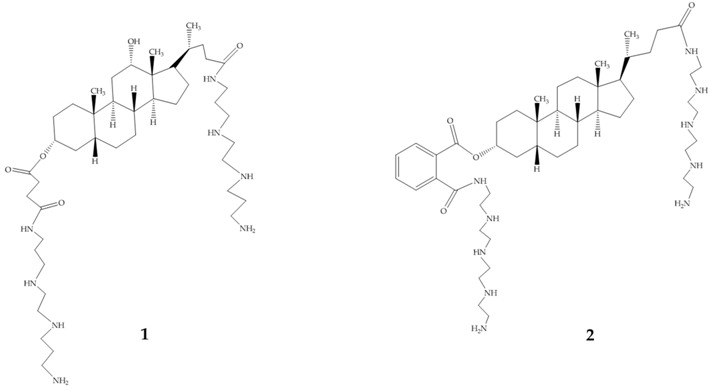


**Acknowledgments:** This work was supported by FCOMP-01-0124-FEDER-041068—EXPL/QEQ-MED/1068/2013.

### 4.31. NMR Spectral Assignments of Anacardic Acids Obtained from Cashew Nut Shell Liquid and Effect of Different Unsaturation on their Biological Activities (P51)

Selene Maia de Morais ^1^^,^*, Katherine Alves Silva ^1^, Halisson Araujo ^1^, Ícaro Gusmão Pinto Vieira ^1^ and Artur Manuel Soares Silva ^2^

^1^ Curso de Química, Universidade Estadual do Ceará Av. Silas Munguba 1700, 60714-903 Fortaleza, Brasil

^2^ Departamento de Química & QOPNA, Universidade de Aveiro, Campus Universitário de Santiago 3810-193 Aveiro, Portugal

* Correspondence: selenemaiademorais@gmail.com

Cashew nut shell liquid (CNSL) is obtained in the cashew nut processing industries as a by-product of little commercial value but with high technological potential due to its phenolic constitution and its various biological properties such as antimicrobial, anti-inflammatory, anti-tumor, antioxidant, anti-acetylcholinesterase, etc (Kubo, I*., et al.* *Food Chem*. 2006, *99*, 555–562; Kubo, I., *et al. J. Agric. Food Chem.* 1993, *41*, 1012–1015; Oliveira, M.S.C., *et al.* *Acta Trop.* 2010, *117*, 165–170). The anacardic acid, the major constituent of CNSL, is a mixture of three derivatives of salicylic acid with side chains of fifteen carbon atoms presenting different degree of unsaturation. In this study, the extraction of natural CNSL from cashew shell was made with hexane by maceration for obtaining anacardic acid (Paramashivappa, P., *et al.* *J. Agric. Food Chem.* 2001, *49,* 2548–2551). The major components of anacardic acid (15:1, 15:2 and 15:3) were isolated by means of column chromatography impregnated with silver nitrate, its structures were characterized by nuclear magnetic resonance, making a complete assignment of proton and carbon atoms in the spectra. It was evaluated the effect of unsaturation of the side chain in the biological activities such as antioxidant, anticholinesterasic and toxicity against *Artemia salina*. Antioxidant activity was assessed by inhibition of the free radical DPPH (1,1-diphenyl-2-picryl-hydrazyl): monoene anacardic acid presents an IC_50_ = 2.06 ± 0.28 mg/mL, similar to the diene with IC_50_ = 1.78 ± 0.01 mg/mL, the triene was more active with IC_50_ = 0.13 ± 0.81 mg/mL, and the standard BHT showed an IC_50_ = 0.266 ± 0.005 mg/mL. The cytotoxicity against the *A. salina* was higher for anacardic acid triene with IC_50_ of 109.71 mg/mL, and even lower, a cytotoxic effect was also observed for other constituents which present LC_50_ <1000 g/mL (Meyer, B.N. *et al.* *Planta Med*. 1982, *45*, 31–34). Regarding antiacetilcolinesterase activity, the triene anacardic acid showed the highest inhibition zone (1.0 cm), when compared to standard physostigmine (0.9 cm). Thus, among anacardic acids, triene showed better biological activities, followed by the diene and monoene. Therefore the greater the number of unsaturations higher will be its action against free radicals, enzymes and organisms tested.

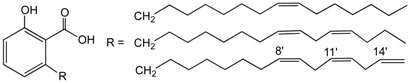


**Acknowledgments:** We thank the Conselho Nacional do Desenvolvimento Científico e Tecnológico (CNPq) for financial support.

### 4.32. First Evidence of Efavirenz Metabolism to a Catechol—A Plausible Role in Toxicity (P52)

Shrika G. Harjivan ^1,^*, Riccardo Wanke ^1^, Erica Torres ^1^, Kellie Woodling ^2^, Gonçalo Gamboa da Costa ^2^, Frederick A. Beland ^2^, Alexandra M. M. Antunes ^1^ and Maria Matilde Marques ^1^

^1^ Centro de Química Estrutural, Instituto Superior Técnico, Universidade de Lisboa, P1049-001 Lisboa, Portugal

^2^ National Center for Toxicological Research, Jefferson, AR 72079, USA

* Correspondence: shrika.harjivan@tecnico.ulisboa.pt

Efavirenz (EFV, **1**) is a non-nucleoside reverse transcriptase inhibitor administered as first-line treatment against HIV and prescribed as part of combination therapy. EFV is extensively metabolized by cytochrome P450, undergoing primary oxidation on the aromatic ring to the phenolic products 7-OH-EFV (minor, **2**) and 8-OH-EFV (major, **3**), and secondary oxidation on the cyclopropane ring (at C14) to 8,14-diOH-EFV (**4**). Despite the drug’s efficacy, clinically restrictive neurotoxic and hepatotoxic events are a major limitation of EFV administration. Bioactivation to reactive electrophiles (e.g., to catechol metabolites and their quinoid derivatives) capable of reacting with biomacromolecules is likely to be involved.

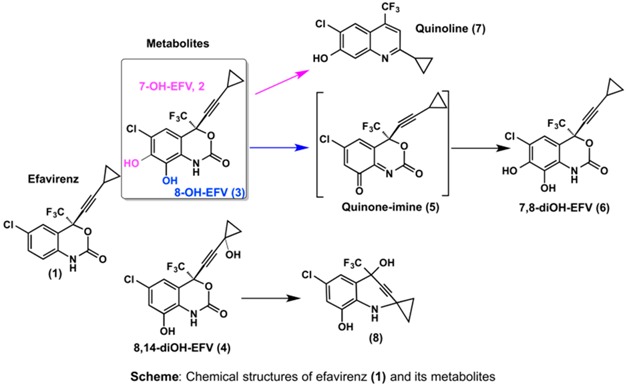


We obtained and fully characterized a new catechol (**6**) from direct oxidation of 8-OH-EFV with Frémy´s salt; its quinone-imine precursor (**5**) was also detected. Incubation of EFV and its mono-hydroxy metabolites **2** and **3** with rat and human liver microsomes, followed by LC-ESI-MS characterization, led to the first-time evidence for the formation of catechol **6** in metabolically competent systems. By contrast, direct oxidation of **2** afforded a stable quinoline derivative (**7**) (Harjivan, S.G., *et al.* *Eur. J. Med. Chem*. 2014, *74*, 7–11) but no evidence of the catechol or its quinone-imine precursor was obtained. In addition, 8,14-diOH-EFV (**4**) afforded product (**8**) under bio-mimetic conditions. Given the propensity of quinone-imines to undergo nucleophilic addition, our results support a role for bioactivation of 8-OH-EFV at the onset of EFV-mediated toxicity.

**Acknowledgments:** We thank the Portuguese NMR and MS Networks (IST-UL nodes) for providing access to the facilities. This work was supported in part by Fundação para a Ciência e a Tecnologia (FCT), Portugal, through grants UID/QUI/00100/2013, RECI/QEQ-QIN/0189/2012 and RECI/QEQ-MED/0330/2012, and fellowships to RW (SFRH/BPD/70953/2010) and SGH (SFRH/BD/80690/2011). AMMA also acknowledges FCT, “Programa Operacional Potencial Humano” and the European Social Fund (Program IF/01091/2013).

### 4.33. STD-NMR and Fluorescence Quenching Towards Understanding Astringency: a Molecular Study on Procyanidins/Salivary Protein (mucin) Interaction (P54)

Susana Soares *, Elsa Brandão, Nuno Mateus and Victor de Freitas

REQUIMTE/LAQV, Departamento de Química e Bioquímica, Faculdade de Ciências da Universidade do Porto, Rua do Campo Alegre 689, 4169-007 Porto, Portugal

* Correspondence: Susana.soares@fc.up.pt

Procyanidins (PC) are polyphenols (polymeric flavan-3-ols) that enter the human diet through fruits and vegetable-based beverages like red wine. These compounds have the ability to interact with salivary proteins. This interaction is related to astringency sensation defined as dryness, tightening and puckering sensations perceived in the oral cavity during the ingestion of food/beverages.

Most of the works done in astringency research do not characterize this interaction at molecular level. So, herein was intended to study the interaction between mucin (salivary) protein and PC by fluorescence quenching and Saturation Transference Difference-NMR. Thus, we focused in synthesize/isolate PC commonly present in red wine (dimers B3/B4, tetramer and oligomeric PC) and characterize their interaction with mucin.

The results showed that the values of binding constants obtained by fluorescence extinction increase with PC polymerization degree. Hydrophobic and hydrogen bonds were identified as the major driving forces for the interaction.

### 4.34. Synthesis and Spectroscopic Characterization of a New Dithiosquarylium Cyanine Dye (P55)

Tânia Fernandes ^1,2^, Tiago Martins ^1,2^, Renato Boto ^3^, Paulo Almeida ^3^, José Fernandes ^4^, Amélia Silva ^2^ and Lucinda Reis ^1,^*

^1^ CQ-VR and Department of Chemistry, UTAD, Quinta de Prados, 5000-801 Vila Real, Portugal

^2^ CITAB and Department of Biology and Environment, UTAD, Quinta de Prados, 5000-801 Vila Real, Portugal

^3^ CICS-UBI—Health Sciences Research Centre, University of Beira Interior, Av. Infante D. Henrique, 6200-506 Covilhã, Portugal

^4^ INESC-TEC Porto and Department of Physics, UTAD, Quinta de Prados, 5000-801 Vila Real, Portugal

* Correspondence: lucinda.reis@utad.pt

The discovery of new and better photosensitizers for alternative therapies, such as Photodynamic Therapy (PDT), has aroused the interest of the scientific community. The squarylium cyanines dyes possess some of the essential properties for a good PDT photosensitizer, including a strong absorption in the Visible (Vis) or NIR region and the significant production of reactive oxygen species (Santos, P., *et al.* *J. Photochem. Photobiol. A* 2003, *160*, 159–161). However, some modifications in their structure may improve these properties enhancing its biological application. Previous studies suggest that the replacement of the oxygen atoms bonded at central ring by sulfur atoms increase the singlet oxygen production (Webster, S., *et al.*, *J. Phys. Chem.* *Lett.* 2010, *1*, 2354–2360).

In this study a new symmetric dithiosquarylium cyanine dye **4** was synthesized with a moderated yield and its structural characterization was elucidated by, melting point, IR, Vis, ^1^H-NMR, ^13^C-NMR and HRMS-ESI-TOF. The citotoxicity in dark and under by LEDs irradiation, with an appropriate wavelength, was also evaluated in the cell lines HepG2 and Caco-2.

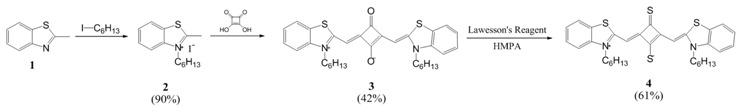


### 4.35. Synthesis of a New Squarylium Cyanine Dye and Cytotoxicity Evaluation in HepG2 and Caco-2 Cells (P56)

Tiago Martins ^1,2^, Tânia Fernandes ^1,2^, Renato Boto ^2^, Paulo Almeida ^2^, José Fernandes ^3^, Amélia Silva ^4^ and Lucinda Reis ^1,^*

^1^ CQ-VR and Department of Chemistry, UTAD, Quinta de Prados, 5000-801 Vila Real, Portugal

^2^ CICS-UBI – Health Sciences Research Centre, University of Beira Interior, Av. Infante D. Henrique, 6200-506 Covilhã, Portugal

^3^ INESC-TEC Porto and Department of Phisics, UTAD, Quinta de Prados, 5000-801 Vila Real, Portugal

^4^ CITAB-UTAD and Department of Biology and Environment, UTAD, Quinta de Prados, 5000-801 Vila Real, Portugal

* Correspondence: lucinda.reis@utad.pt

The continuous study and development of organic chemistry has enabled the discovery of compounds with different applications. Among them, the discovery of new synthetic dyes, such as squarylium cyanine dyes which have shown great interest in many areas and applications (Chang, J., *et al.* *J. Power Sources* 2013, *240*, 779–785). The squarylium cyanine dyes are a class of compounds, discovered in 1965 by Treibs (Treibs, A., *et al.* *Angew. Chem. Int. Ed.* 1965, *4*, 694), whose photochemical and photophysical properties showed ideal behaviour for many biotechnological applications, such as photodynamic therapy (Avirah, R. R., *et al.* *Org. Biomol. Chem.* 2012, *10*, 911–920).

In this work a new squarylium cyanine dye **7** was synthesized and fully characterized by melting point, IR, Vis, ^1^H-NMR, ^13^C-NMR and HRMS-ESI-TOF spectra. This dye was obtained, according to methods adapted from literature (Tatarets, A., *et al.* *Dyes Pigment.* 2005, *64*, 125–134), with moderate yield. Later, its cytotoxic effect was evaluated on Caco-2 and HepG2 cells, in the dark and after LEDs irradiation with an appropriate wavelength.

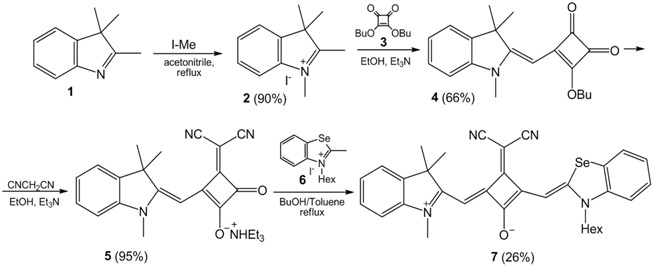


### 4.36. Synthesis of Biflorin-Based Nitrogen Derivatives and Their Antibacterial Activity (P57)

Luciana G. da S. Souza ^1^, Macia C. S. Almeida ^1^, Telma L. G. Lemos ^1,^*, Artur M. S. Silva ^2^, Vera L. M. Silva ^2^, Paulo R. V. Ribeiro ^3^, José G. M. Costa ^4^ and Raimundo Braz-Filho ^5^

^1^ Departamento de Química Orgânica e Inorgânica, Universidade Federal do Ceará, Campus do Pici, 60451-970 Fortaleza, Brazil

^2^ Departamento de Química & QOPNA, Universidade de Aveiro, 3810-193 Aveiro, Portugal

^3^ Embrapa Agroindustria Tropical, R Dra Sara Mesquita, 2270, 60511-110 Fortaleza, Brazil

^4^ Laboratório de Pesquisa de Produtos Naturais, Universidade Regional do Cariri, 63105-000 Crato, Brazil

^5^ Universidade Estadual do Norte Fluminense, Av. Alberto Lamego 2000, 28013-603 Campos dos Goytacazes, Brazil

* Correspondence: tlemos@dqoi.ufc.br

The quinones represent a wide and varied family of secondary metabolites of natural occurrence. The interest in these substances has intensified in recent years due to their pharmacological importance and great structural variety. Several natural and synthetic quinones possess potent and diverse pharmacological effects such as antitumor (Ferreira, V.F., *et al.* *Quim. Nova* 2003, *26*, 407–416; Asche, C., *Mini Rev. Med. Chem.* 2005, *5*, 449–467; Costa-Lotufo, L.V., *et al.* *Biol. Pharm. Bull.* 2007*, 30,* 1416–1421), anti-inflammatory (Almeida, E.R., *et al.* *J. Ethnopharmacol.* 1990, *29,* 239–241; Acosta, S.L., *et al. Acta Farm. Bonaer.* 2003, *22*, 53–55), analgesic (Acosta, S.L., *et al.* *Fitoterapia* 2003, *74*, 686–688), antifungal (Nianga, M., *et al. Phytochemistry* 1996, *42*, 1315–1320; Tandon, V.K., *et al.* *Eur. J. Med. Chem.* 2009, *44*, 3130–3137) and trypanocidal (Ferreira, V.F., *et al.* *Bioorg. Med. Chem.* 2006, *14*, 5459–5466; Menna-Barreto, R.F.S., *et al. J. Proteom.* 2010, *73*, 2306–2315). Biflorin **1** is a quinone, biologically active, isolated from *Capraria biflora* L.. Five new biflorin-based nitrogen derivatives were synthesized; two biflorin derived hydrazones **2a**,**b** and three biflorin derived oximes **3a**–**c**. In the case of the hydrazones **2a**,**b** only the *Z* isomers were obtained, while the oximes **3b**,**c** exhibit mixtures of *E* and *Z* isomers. The compounds were characterized by 1D and 2D NMR spectroscopy and mass spectrometry. Their antibacterial activity was also investigated using the microdilution method for determining the MIC against six bacterial strains. Tests have shown that these derivatives have potential against all bacterial strains.

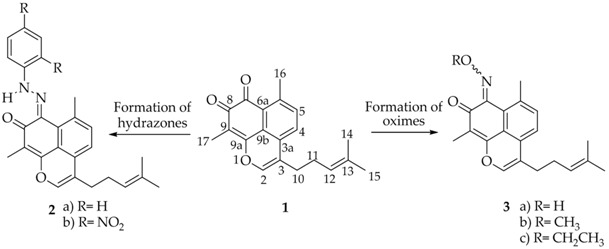


### 4.37. Enantiopure Tryptophanol-Derived Small Molecules: Synthesis and Cytotoxicity Evaluation (P59)

Valentina Barcherini ^1^, Margarida Espadinha ^1^, Lucília Saraiva ^2^, Lidia Gonçalves ^1^ and Maria M.M. Santos ^1^^,^*

^1^ Research Institute for Medicines (iMed.ULisboa), Faculty of Pharmacy, Universidade de Lisboa, Av. Gama Pinto, 1649-003 Lisboa, Portugal

^2^ UCIBIO/REQUIMTE, Departamento de Ciências Biológicas, Faculdade de Farmácia da Universidade do Porto, Rua de Jorge Viterbo Ferreira, 4050-313 Porto, Portugal

* Correspondence: mariasantos@ff.ulisboa.pt

1,2-Aminoalcohols containing a stereogenic centre can be used as chiral auxiliaries/inductors to control the stereochemical course of a diastereoselective reaction (Ager, D.J., *et al.* *Chem. Rev.* 1996, *96*, 835–876). Using this strategy, our research group has recently developed several biologically active small molecules starting from enantiopure aminoalcohol tryptophanol (Pereira, N.A.L., *et al.* *ChemMedChem* 2015, *10*, 2080–2089; Pereira, N.A.L., *et al.* *Bioorg. Med. Chem. Lett.* 2014, *24*, 3333–3336; Soares, J., *et al. Pharmacol. Res*. 2015, *95–96*, 42–52; Soares, J., *et al. Oncotarget* 2015, doi:10.18632/oncotarget.6775). Herein, we will present our most recent results on the synthesis of (*S*) and (*R*)-tryptophanol-derived compounds, which were synthesized by cyclocondensation reaction of enantiopure forms of tryptophanol and several γ-ketoacids. The chiral inductor (tryptophanol) besides being responsible for the stereo-outcome of the final product, it is also part of the main skeleton of biologically active molecules. For those reasons, this asymmetric reaction is highly efficient/atom economic. This specific one-step synthesis approach allows an asymmetric strategic construction of a new chiral centre. The new family of compounds synthesized was screened for cytotoxicity activity. The results show that are compounds are promising small molecules for the development of novel anticancer agents.

### 4.38. A New Curcumin Derivative with More Potent Antitumor and Anti-P-glycoprotein Activity Than Curcumin (P62)

Vanessa Lopes-Rodrigues ^1,2,3^, Ana Oliveira ^4^, Marta Correia-da-Silva ^4,5^, Madalena Pinto ^4,5^, Raquel T.Lima ^1,2,6^, Emília Sousa ^4,5^ and M. Helena Vasconcelos ^1,2,7,^*

^1^ i3S—Instituto de Investigação e Inovação em Saúde, Universidade do Porto, R. Alfredo Allen, 4200-135 Porto, Portugal

^2^ Cancer Drug Resistance Group, IPATIMUP—Institute of Molecular Pathology and Immunology of the University of Porto, IPATIMUP, 4200-465 Porto, Portugal

^3^ ICBAS-UP—Institute of Biomedical Sciences Abel Salazar, University of Porto, ICBAS-UP, 4099-003 Porto, Portugal

^4^ Laboratório de Química Orgânica e Farmacêutica, Departamento de Ciências Químicas, Faculdade de Farmácia da Universidade do Porto, Rua Jorge Viterbo Ferreira 228, 4050-313 Porto, Portugal

^5^ CIIMAR, Centro Interdisciplinar de Investigação Marinha e Ambiental (CIIMAR/CIMAR), Universidade do Porto, Rua dos Bragas 289, 4050-123 Porto, Portugal

^6^ Department of Pathology and Oncology, FMUP—Faculty of Medicine of the University of Porto, Porto, Portugal, Alameda Hernâni Monteiro, 4200-319 Porto, Portugal

^7^ Department of Biological Sciences, FFUP—Faculty of Pharmacy, University of Porto, 4050-313 Porto, Portugal

* Correspondence: hvasconcelos@ipatimup.pt

Cancer multidrug resistance (MDR) is a major limitation to the success of chemotherapy and may be highly associated with the overexpression of drug efflux pumps such as P-glycoprotein (P-gp) (Lopes-Rodrigues, V., *et al.* *Int. J. Cancer* 2014, *135*, 253–263). Curcumin is a secondary metabolite with antitumor activity and MDR modulatory activity. However, curcumin is very unstable and has poor bioavailability (Limtrakul, P., *et al.* *Adv. Exp. Med. Bio.* 2007, *595*, 269–300).

This work aimed to synthesizing new curcumin derivatives and analogues more stable than curcumin itself and presented dual activity: antitumor and P-gp inhibitor. Stability and photostability studies were conducted, by comparing curcumin with two synthesized building blocks. To analyse the biological effect of the compounds, cell viability was investigated in a pair of MDR and drug sensitive cell lines (chronic myeloid leukemia) and drug-efflux activity of P-gp was analyzed. One of the curcumin derivatives showed more potent antitumor and anti-P-gp activity than curcumin itself.

### 4.39. Enantioselective Synthesis of Enantiopure Bicyclic Lactams and Evaluation as NMDA Receptor Antagonists (P63)

Margarida Espadinha ^1,^*, Rocio-Lajarin Cuesta ^2^, Cristóbal de los Ríos ^2^ and Maria M. M. Santos ^1,^*

^1^ Research Institute for Medicines (iMed.ULisboa), Faculty of Pharmacy, Universidade de Lisboa, Av. Prof. Gama Pinto, 1649-003 Lisboa, Portugal

^2^ Facultad de Medicina, Universidad Autonoma de Madrid, 28029 Madrid, Spain

* Correspondence: mespadinha@ff.ul.pt; mariasantos@ff.ulisboa.pt

An overactivation of *N*-Methyl-d-Aspartate (NMDA) receptors is associated with major degenerative disorders, including Parkinson’s and Alzheimer’s diseases. So, the design of NMDAR antagonists that can modulate the excitotoxicity phenomena without disturbing the normal NMDAR physiologic functioning in the regulation of synaptic plasticity seems to be a promising therapeutic approach. Unfortunately, the majority of NMDAR antagonists synthesized so far exhibit poor pharmacokinetic profiles, low selectivity towards to other receptors and also side effects (Olivares, D., *et al. Curr. Alzheimer Res.* 2012, *9*, 746–758).

In the last years, our research group has been working in the development of novel small molecules that act as NMDA receptor antagonists (Pereira, N.A.L., *et al.* *Monatsh. Chem.* 2013, *144*, 473–477). Herein, we will present our most recent results on the hit-to-lead optimization of a novel NMDA receptor antagonist recently identified by our group (Pereira, N.A.L., *et al.* *Bioorg. Med. Chem. Lett.* 2014, *24*, 3333–3336). The hit compound revealed to be 1.5 more active than amantadine (a NMDA receptor antagonist used in the clinic). Twenty six derivatives were synthesized by cyclocondensation reaction of the appropriate enantiopure amino alcohols and several keto-acids, with good to excellent yields. These cyclocondensation reactions allow the stereoselective formation of a new chiral center. The compounds were evaluated as NMDA receptor antagonists. The most promising compound revealed to be 5.5 times more active than amantadine.

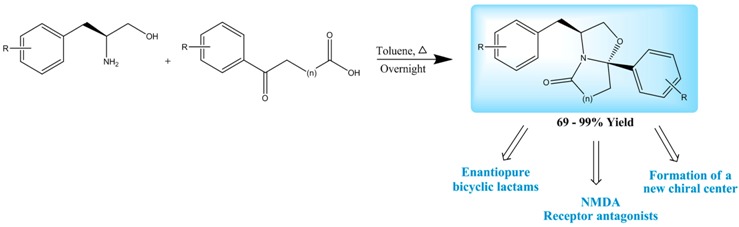


### 4.40. Therapeutic Bioconjugates: Evaluation of Iminoboronates as Payload Delivery System for Cancer (P64)

Ricardo M.R.M. Lopes*, Pedro M.S.D Cal. and Pedro M.M Gois*.

Research Institute for Medicines (iMed.ULisboa), Faculty of Pharmacy, Universidade de Lisboa, Lisboa, Portugal, Av. Gama Pinto, 1649-003 Lisboa, Portugal

* Correspondence: rmr.lopes@campus.fct.unl.pt (L.R.M.R.M.); pedrogois@ff.ul.pt (G.P.M.M.)

Chemotherapy uses small potent molecules with high activity towards specific tumour targets. However, with such high activity comes high off target toxicity and severe side effects. Fortunately, chemotherapy can be now targeted thanks to powerful linkers that connect a ligand molecule with affinity to interesting biological receptors and a cytotoxic drug. This linkers must have very specific properties, such as high stability in plasma, no toxicity, no interference with ligand affinity nor drug potency, and at the same time, be able to self-lyse once inside the target cell. Bipolar environments as seen between tumoural extracellular and intracellular medias are usually exploited by this linkers in order to release the therapeutic warhead.

This work explores a new model for the same task, specific cancer drug delivery (Gois, P.M.M., *et al.* *Chem. Commun.* 2014, *50*, 5261–5263). Iminoboronates were studied due to its remarkable selective stability towards a wide pH range and endogenous molecules (Gois, P.M.M., *et al.* *J. Am. Chem. Soc.* 2012, *134*, 10299–10305). Bioconjugates were design to prove this iminoboronate linker’s effectiveness. The ability to be uptaken by a cancer cell through endocytosis process and delivery of specific payload are two features expected for this construct.

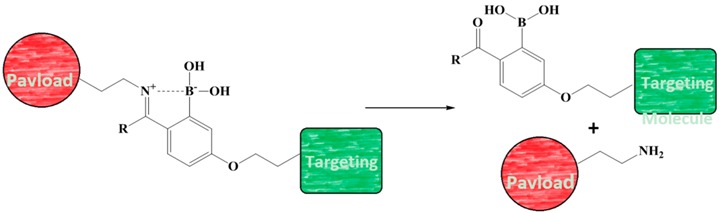


### 4.41. Laminariales and Fucales Algae of Portuguese Coast for Neurodegenerative Disorders Management (P68)

Fátima Fernandes ^1^, Mariana Barbosa ^1^, David M Pereira ^1^, Patrícia Valentão ^1^, Isabel Sousa-Pinto ^2,3^ and Paula B Andrade ^1,^*

^1^ REQUIMTE/LAQV, Laboratório de Farmacognosia, Departamento de Química, Faculdade de Farmácia, Universidade do Porto, Rua de Jorge Viterbo Ferreira n.º 228, 4050-313 Porto, Portugal

^2^ Interdisciplinary Centre for Marine and Environmental Research (CIIMAR/CIMAR), Rua dos Bragas, n.º 289, 4050-123 Porto Portugal

^3^ Faculty of Sciences, University of Porto, Rua do Campo Alegre, n.º 1021/1055, 4169-007 Porto, Portugal

* Correspondence: pandrade@ff.up.pt

Macroalgae have been widely acknowledged as an important group of organisms for marine drug development. Among the great chemical diversity, algal pigments such as carotenoids and chlorophylls are highlighted. This work aimed at determining the pigment profile of algae species found in Portuguese waters, namely three kelps (*Laminaria ochroleuca* Bachelot de la Pylaie, *Saccharina latissima* (L.) C.E. Lane, C. Mayes, Druehl & G.W. Saunders, and *Saccorhiza polyschides* (Lightfoot) Batters), and other three belonging to the order Fucales (*Fucus guiryi* G.I. Zardi, K.R. Nicastro, E.S. Serrão & G.A. Pearson, *Fucus serratus* L. and *Fucus spiralis* L.). In addition, their effect against cholinesterases and lipoxygenase, as well as their neuroprotective potential in SH-SY5Y cells, were tested.

Fucoxanthin (**1**) was the most representative carotenoid in all samples. In general, algae acetone extracts showed a reduced inhibitory effect over cholinesterases and a potent inhibition of lipoxygenase. At non-toxic concentrations, both extracts and fucoxanthin revealed protective effect in SH-SY5Y cells insulted with glutamate, *L. ochroleuca* being the most promising one. These results point to the potential interest of the exploitation and application of these marine natural matrices in pharmaceutical and nutraceutical preparations with health benefits.

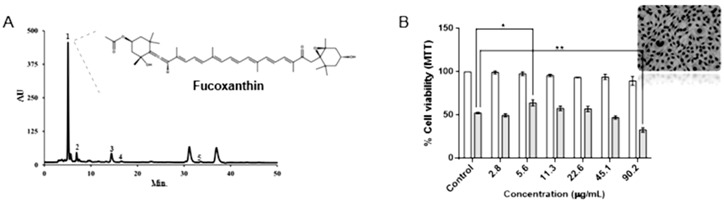

(**A**) HPLC-DAD chromatogram of the acetone extract of *L. ochroleuca*. Detection at 450 nm. (**1**) Fucoxanthin; (**2**) Violaxanthin; (**3**) Chlorophyll *a*; (**4**) Chlorophyll *a* derivative; (**5**) β-carotene. (**B**) Effect of *L. ochroleuca* acetonic extract on the viability of SH-SY5Y cells. Values represent mean ± SEM (*n* = 4). The average values were significantly different compared to control (* *p* < 0.05; ** *p* < 0.01).

**Acknowledgments:** This work received financial support from the European Union (FEDER funds through COMPETE) and National Funds (FCT, Fundação para a Ciência e Tecnologia) through project UID/QUI/50006/2013. Fátima Fernandes (SFRH/BPD/98732/2013) and Mariana Barbosa (SFRH/BD/95861/2013) thank FCT for the grants.

### 4.42. Exploring the Chemistry of Furans: Synthesis of Functionalized Bis(furan-2-yl)methanes and 1,6-Dihydropyridazines (P70)

Américo J. S. Alves *, Susana M. M. Lopes and Teresa M. V. D. Pinho e Melo

Centro de Química de Coimbra e Departamento de Química, Universidade de Coimbra, 3004-535 Coimbra, Portugal

* Correspondence: americo_jsa@hotmail.com

Furan is one of the biomass-derived platform molecules, which is easily produced by decarbonylation of furfural. Furthermore, the rich chemistry of this heterocyclic compound makes it a particularly interesting scaffold in organic synthesis, leading to a wide range of compounds some of which finding application in natural product synthesis and medicinal chemistry. Herein, bringing together our interest in the chemistry of furans and conjugated nitroso- and azoalkenes the study of the reactivity of bis(furan-2-yl)methanes towards these reactive intermediates is described (Gallezot, P., *Chem. Soc. Rev.* 2012, *41*, 1538–1558; Butin, A.V., *et al.* *Eur. J. Org.* *Chem*. 2015, 2999–3016).

We have recently described the reactivity of 2,2-(difuran-2-yl)propane towards nitroso- and azoalkenes (Pinho e Melo, T., *et al.*, *Eur. J. Org. Chem.* 2015, 6146–6151). The expected hetero-Diels-Alder adducts, were obtained in good yields. The 4a,7a-dihydro-4*H*-furo[2,3-*e*][1,2]oxazines were easily converted into the corresponding bis(furan-2-yl)methanes bearing an open-chain oxime. On the other hand, 4a,7a-dihydrofuro[3,2-*c*]pyridazines undergo an acid or base-catalyzed reaction to give 6-(2-oxobutyl)-1,6-dihydropyridazines in a process involving furan ring-opening reactions. The synthesis of 4a,7a-dihydrofuro[3,2-*c*]pyridazines from 2-methylfuran and the subsequent acid-catalyzed rearrangement to the corresponding 6-(2-oxopropyl)-1,6-dihydropyridazines was also achieved.

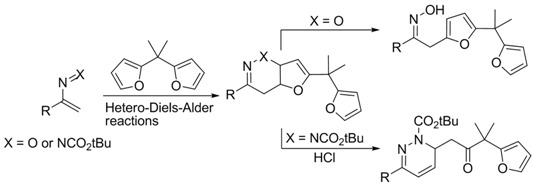


## 5. Conclusions

During the meeting, the following prizes were awarded:

- Portuguese Award for Best Portuguese Young Organic Chemist. This prize is awarded to a young scientist (young doctor) with an outstanding curriculum vitae. The prize in 2015 goes to Maria Manuel Santos from Medicinal Chemistry Group, Faculty of Phamacy, Univeristy of Lisbon, Portugal.
- Portuguese Award for Best Ph.D. Thesis in Organic Chemistry 2015: This prize was awarded to Jaime Marques Coelho for the Ph.D. Thesis entitled: “New synthethic methodologies for the transformation of biomass derived intermediates to valuable molecules”. Ph.D. in Phamacy, Faculty of Phamacy, Univeristy of Lisbon, Portugal.
- Portuguese Award for Best Master Thesis in Organic Chemistry 2015: This prize was awarded to Emanuel Pinto de Sousa for the Master’s Thesis entitled: “Synthesis of hexacyclic chiral steroids with potential anticancer activity”. Master in Medicinal Chemistry, Chemistry Department, University of Coimbra, Portugal.
- Prize for the best poster in chromatography: This prize was awarded to João P. Nunes, Catarina Charneira, Inês L. Martins, Cristina C. Jacob, M. Matilde Marques, Alexandra M.M. Antunes for the poster entitled: “Enrichment of Glycidamide Adducts of Human Serum Albumin”, CQE-IST, Centro de Química Estrutural, Instituto Superior Técnico, Universidade de Lisboa, Portugal.
- Prize for the best poster in mass spectrometry: This prize was awarded to M. R. Pérez-Gregorio, R. Días, N. Mateus, V. de Freitas for the poster entitled: “Identification of complexes formed between salivary proteins and procyanidin B3 by mass spectrometry. Effect of profile saliva and tannin concentration”, LAQV-REQUIMTE, Departamento de Quimica e Bioquimica. Faculdade de Ciências, Universidade do Porto, Porto, Portugal.
- Prize for the best poster in Medicinal Chemistry: This prize was awarded to Elisabete Ferreira, Pedro F. Cruz, Zaida L. Almeida, Marta S. Sousa, Rui M. M. Brito for the poster entitled: “Identification and characterization of small molecule interactions with transthyretin amyloid fibrils”, Coimbra Chemistry Centre, Chemistry Department, University of Coimbra, Portugal.

In 2017 the 12^th^ National Meeting of Organic Chemistry will be held in Coimbra.

